# Approaching Angstrom-Scale Resolution in Lithography Using Low-Molecular-Mass Resists (<500 Da)

**DOI:** 10.1021/acsnano.4c03939

**Published:** 2024-08-20

**Authors:** Mohammad S.M. Saifullah, Anil Kumar Rajak, Kevin A. Hofhuis, Nikhil Tiwale, Zackaria Mahfoud, Andrea Testino, Prajith Karadan, Michaela Vockenhuber, Dimitrios Kazazis, Suresh Valiyaveettil, Yasin Ekinci

**Affiliations:** † Paul Scherrer Institut, Forschungsstrasse 111, Villigen PSI 5232, Switzerland; ‡ PiBond Oy, Kutojantie 2B, Espoo 02630, Finland; § Center for Functional Nanomaterials, Brookhaven National Laboratory, Upton, New York 11973-5000, United States of America; ∥ Institute of Materials Research and Engineering, A*STAR (Agency for Science, Technology, and Research), 2 Fusionopolis Way, #08-03 Innovis, Singapore 138634, Republic of Singapore; ⊥ École Polytechnique Fédérale de Lausanne, STI SMX-GE, Lausanne CH 1015, Switzerland; # Department of Chemistry, National University of Singapore, 3 Science Drive 3, Singapore 117543, Republic of Singapore

**Keywords:** low-molecular-mass resists, extreme ultraviolet lithography, electron beam lithography, metal oximate, radical initiator, nanofabrication

## Abstract

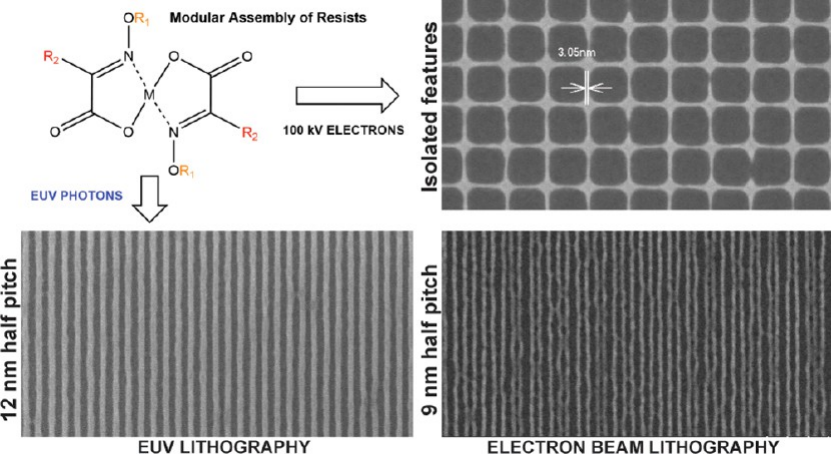

Resists that enable high-throughput and high-resolution patterning are essential in driving the semiconductor technology forward. The ultimate patterning performance of a resist in lithography is limited because of the trade-off between resolution, line-width roughness, and sensitivity; improving one or two of these parameters typically leads to a loss in the third. As the patterned feature sizes approach angstrom scale, the trade-off between these three metrics becomes increasingly hard to resolve and calls for a fundamental rethinking of the resist chemistry. Low-molecular-mass monodispersed metal-containing resists of high atom economy can provide not only very high resolution but also very low line-width roughness without sacrificing sensitivity. Here we describe a modular metal-containing resist platform (molecular mass <500 Da) where a molecular resist consists of just two components: a metal and a radical initiator bonded to it. This simple system not only is amenable to high-resolution electron beam lithography (EBL) and extreme ultraviolet lithography (EUVL) but also unites them mechanistically, giving a consolidated perspective of molecular and chemical processes happening during exposure. Irradiation of the resist leads to the production of secondary electrons that generate radicals in the initiator bonded to metal. This brings about an intramolecular rearrangement and causes solubility switch in the exposed resist. We demonstrate record 1.9–2.0 nm isolated patterns and 7 nm half-pitch dense line-space features over a large area using EBL. With EUVL, 12 nm half-pitch line-space features are shown at a dose of 68 mJ/cm^2^. In both of these patterning techniques, the line-width roughness was found to be ≤2 nm, a record low value for any resist platform, also leading to a low-performance trade-off metric, *Z* factor, of 0.6 × 10^–8^ mJ·nm^3^. With the ultimate resolution limited by instrumental factors, potential patterning at the level of a unit cell can be envisaged, making low-molecular-mass resists best poised for angstrom-scale lithography.

## 1 Introduction

Lithography has had a world-changing impact in enabling over five decades of relentless progress in semiconductor technology. The process used to print electronic device components and circuits with accuracy is the epitome of ultraprecision manufacturing. The latest of the embodiments, extreme ultraviolet lithography (EUVL), is quintessential for producing the fastest and most powerful computer chips to support the revolution in artificial intelligence.^1−3^ Although the state-of-the-art chips already feature sub-10 nm structures, the bottleneck in throughput and reliability in patterning these nanostructures has recently been the resist materials. Pushing forward into achieving angstrom-scale resolution material patterning can unlock a plethora of opportunities as the microelectronic industry delves into the quantum computing era.

Lithography techniques are based on projecting and recording an optical image onto a resist. The quality of the recorded image determines the quality of electronic components fabricated using subsequent processing steps on the chip. In the semiconductor industry, organic polymers make up the majority of the basic components of conventional resists. Chemically amplified resists (CARs) currently form the backbone of the lithography infrastructure. They consist of a polymer matrix with protected groups, photoacid generators (PAGs), and quenchers.^4^ When exposed to deep ultraviolet (DUV) or EUV radiation, PAGs release protons. The protons diffuse and change the solubility of functional groups during the postexposure bake step. This leads to the transformation of hydrophobic protected groups to hydrophilic deprotected groups, and during development, the latter get dissolved, leaving behind the unexposed region that has its characteristic undulations, which is referred to as line-width roughness (LWR).

There are three critical metrics that define a successful resist: resolution, *R*; LWR; and sensitivity (i.e., dose-to-size), *S*. It is very difficult to improve these three metrics simultaneously. This trilemma is termed the RLS trade-off or the RLS “triangle of death”. These metrics gained a considerable amount of importance when the feature dimensions in lithography started to dip into the sub-50 nm range, especially with the advent of EUVL. There was a rising concern that the resist composition, polymer size, and associated LWR could be a limiting factor to create structures of sufficient integrity.^5−7^ Moreover, with the minimum feature requirement in lithography approaching molecular size in resists, that is, the intrinsic resolution limit of the material, the idea of using small molecules as potential polymer replacements as the primary building blocks of lithographic materials began to garner attention. Specifically, the anticipation that using smaller components in the lithographic materials could potentially result in higher resolution and smoother line edges of the patterned structures has been the primary driving force behind the consideration of small molecules rather than polymers.

The molecular size of the resist material is one of the parameters that determines the resolution limit in lithography. As an illustration, when the polymer chain in a resist is characterized as a sphere, the radius of gyration of the sphere of an organic polymer with a molecular weight of 10^5^ is computed to be ≈10 nm.^8^ This suggests that a 100 nm wide lithographic pattern would fluctuate by at least 10%. In other words, the smaller the size of a molecular resist is, the smaller the pixel that can be written is. Combining this with synthetic control leading to monodispersity and control of stereochemistry would potentially result not only in achieving very high patterning resolution but also in much reduced LWR. Further advantages include high molecular chemical contrast during development and reduced swelling of the resist. The former leads to increased chemical gradient and reduced chemical inhomogeneity at the image boundary, which also contribute to the reduction of LWR. Despite these advantages, low-molecular-weight materials are notoriously difficult to translate into resists for lithography as they tend to crystallize rather than to form smooth films and show limited solubility in organic solvents.^9^ Other potential problems include poor photochemical reactivity and insufficient mechanical integrity of the patterns after development.

In the field of electron beam lithography (EBL), the benefits of low molecular and formula mass resists have long been recognized. Forty years ago, Isaacson and Muray showed 1.5 nm wide lines at 4.5 nm pitch and 2.0 nm diameter holes in NaCl as a self-developing resist in a VG HB5 scanning transmission electron microscope (STEM).^10^ A similar patterning behavior was also illustrated in many other films of inorganic materials using energetic electrons.^11,12^ Interesting as they are, these low-molecular-mass inorganic materials needed an exceptionally high dose to expose and were unsuitable for pattern transfer into other materials. Circumventing these problems, in 1999, Saifullah et al. demonstrated a highly sensitive spin-coatable metal-containing low-molecular-mass resist capable of achieving sub-10 nm resolution, with the patterned resist also serving as a durable etch mask.^13−15^ In 2001, Shirota’s group that specialized in amorphous films of molecular glasses proposed the use of low-molecular-weight organic resists for sub-100 nm lithography.^9^ Subsequently, molecular organic resists also started to appear in EUV lithography in both positive- and negative-tone formats.^16−30^ The silicon oxo-cluster based inorganic hydrogen silsesquioxane (HSQ) resist has persisted as a popular choice for sub-10 nm EBL patterning.^31^ Eventually, various negative-tone inorganic/hybrid metal oxo-cluster EUV sensitive resists were developed.^32−39^ Tin oxo-cluster based formulation, commercialized by Inpria Corp., has seen much success over the years,^33^ popularizing the metal oxo-cluster resist platform. Increasing numbers of resists containing oxo-clusters of metal such as Zr, Hf, and Zn have since been developed and studied.^36,38,39^ A recent EBL study on Ni oxo-cluster has reported 9 nm resolution isolated patterns with LWR of 2.9 nm.^40^ The metal oxide resist remains to be one of the best performing EUV platform according to state-of-the-art results.^41^ The molecular mass of almost all the molecular resists studied in the literature exceeded 1000 Da. A quick review of the extant literature suggests that, barring a couple of examples,^30,33^ the promise and hype surrounding the purported abilities of molecular resists when it came to resolution and LWR did not correspond with the actual empirical achievements. This strongly suggests that the molecular resists are not immune to the RLS trade-off and are subjected to the same rules as other resists.

In this work, we adopt a different approach by paring the resist down to its bare essentials. Here, the resist molecule is composed of only two components: a metal and a radical initiator attached to it. Each of the two components can be independently changed or modified to achieve desired properties in the resist. The radical initiator is based on oximate chemistry. This platform offers plenty of flexibility to change the organic environment around a central metal, thus potentially making it a universal scheme to pattern any metal-containing resist. We present a metal-containing molecular resist platform of low molecular mass (MW < 500 Da) amenable to high-resolution EBL and EUVL.^42^ This molecular resist platform is characterized by *high atom economy*. The atom economy of a negative-tone molecular resist can be defined as the conversion efficiency in an exposure process in terms of all atoms involved in the resist molecule and the atoms that are left behind to generate a pattern after exposure. Resists with high atom economy are preferable as they leave behind sufficient patterned material that can be used for further lithographic processing such as etching. For lithography approaching the angstrom scale, a resist with high atom economy is highly desired as it cuts down the resist thickness to not only avoid pattern collapse but also act as an etch mask.

This exceptionally simple resist platform mechanistically unites EBL and EUVL not only across developers but also with respect to the central metal atom, thus giving an integrated approach to molecular and chemical processes occurring during exposure to the two ionizing radiations. Taking the example of low-molecular-mass zinc oximate complex, we demonstrate 1.9–2.0 nm isolated patterns and 7 nm half-pitch dense line-space features using EBL. With EUVL, we show 12 nm half-pitch features at a dose of 68 mJ/cm^2^, the resolution of the resist being limited by the factors related to the instrument. Both of these patterning techniques showed substantially low LWR ≤ 2 nm compared to widely reported values. Consequently, the *Z*-factor value of 0.6 × 10^–8^ mJ·nm^3^ was obtained with EUVL.

## 2 Results and Discussion

### 2.1 ΨMORE^2^ Platform: Assembling Metal-Containing Molecular Resists Like LEGO Bricks

**Figure 1:**
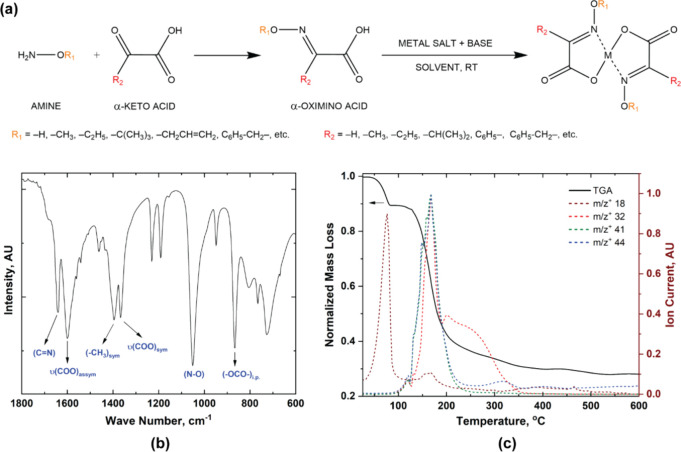
(a) Reaction steps for the synthesis of a metal-containing molecular resist. Here α-keto acid is reacted with an amine to form α-oximino acid, which in turn when reacted with a bivalent metal salt gives metal oximate. (b) FTIR spectrum and (c) TGA-MS of the ZnMIP_2_ resist.

The chemistry of oxime/oximato metal complexes has been a subject of active investigation for more than a century.^43^ One of the convenient ways to synthesize oxime/oximato metal complexes is to first prepare α-oximino acid (or α-keto acid oxime, **R**_**2**_–C(=N–O**R**_**1**_)COOH) by reacting α-keto acid with an amine.^44^ Metal salt is then reacted with α-oximino acid to give the corresponding metal oximate (Figure 1a).^45−47^ Because α-oximino acid or α-keto acid oxime has two donor atoms (the carboxyl oxygen and the oxime nitrogen), it can act as a mono- or bidentate ligand in a metal complex.

Although the skeletal structure of the α-oximino acid molecule, **R**_**2**_–C(=N–O**R**_**1**_)COOH, is very simple, the groups **R**_**1**_ and **R**_**2**_ (from amine and α-keto acid, respectively) here can be changed independently and thus provide the means to assemble metal-containing molecular resists just like the LEGO bricks. For example, with commercially available amines such as hydroxylamine, *O*-methoxyamine, *O*-ethylhydroxylamine, *O*-*tert*-butylhydroxylamine, *O*-allylhydroxylamine, *O*-benzylhydroxylamine, and α-keto acids (or their salts) such as glyoxylic acid, pyruvic acid, phenylglyoxylic acid, phenylpyruvic acid, α-ketobutyric acid, and α-ketoisovaleric acid, 30 different combinations of α-oximino acids can be synthesized. When reacted with a metal salt, these α-oximino acids will give 30 different types of metal-containing molecular complexes that can be potentially employed as resists. Furthermore, the central metal atom can also be replaced with another one, thus making it a universal scheme to assemble metal-containing molecular resists. We have called this resist platform as **ΨMORE**^**2**^ (**PSI M**etal **O**rganic **R**esists for **E**UV and **E**lectron beam lithography). Because the oximate chemistry has been extensively studied, wherever possible, the ΨMORE^2^ resist platform repurposes the metal oximates reported in the literature to perform lithography. The existence of synthesized metal oximates in the literature is incidental.

Using this synthetic route, the degree of flexibility and versatility achieved when it comes to the molecular assembly of a metal-containing resist is unmatched. Such versatility in the molecular assembly of resists also provides further advantages in resist processing. First, by changing the groups **R**_**1**_ and **R**_**2**_, the solubility of the metal complex in an organic solvent can be tailored. Second, the film formability of the complex can also be improved or modified by this method. Third, the sensitivity of a resist to either electrons or EUV photons can be altered by manipulating **R**_**1**_ and **R**_**2**_, especially for the former group. Fourth, the patterned resist when mildly heated results in the formation of functional material, i.e., a metal oxide, thus eliminating the steps of deposition and lift-off to achieve the same.

Any discussion on the versatility and flexibility of such a molecular assembly of a metal-containing resist must be balanced with the requirements of a resist with the lowest possible molecular mass to achieve potentially the highest possible resolution and lowest LWR without sacrificing film formability and sensitivity. Furthermore, molecules with lower mass provide higher atom economy of the resist and, at the same time, leave very little organic residue after exposure to electrons or photons. Another benefit of high atom economy is that thinner resists can be used for high-resolution patterning and their subsequent use as an etch mask for pattern transfer. Bulkier organic groups at **R**_**1**_ and **R**_**2**_ must be avoided as they leave organic residue during development that can lead to scum close to the patterned features.

Using the principles stated above, we systematically synthesized and tested metal-containing oximate complexes (Zn, Ni, In, Al, Mg, and Sn) starting from the least bulky group, i.e., −H, at **R**_**1**_ and **R**_**2**_ and slowly increased the molecular mass of the groups using permutations and combinations. Their solubility and film formability were also evaluated. It was observed that bivalent metal oximate complexes, with both **R**_**1**_ and **R**_**2**_ as −CH_3_ groups, showed good solubility in solvents such as ethylene glycol monomethyl ether (EGME) and propylene glycol monomethyl ether (PGME). On the other hand, trivalent metal oximate complexes demonstrated good solubility in these solvents when **R**_**1**_ and **R**_**2**_ were −H and −CH_3_ groups, respectively, possibly due to the slightly higher organic content. In this study, we will focus only on zinc-based complexes and occasionally make references to complexes of the above-listed metals when necessary.

Zinc-based oximate complexes were prepared via the aqueous route. When both **R**_**1**_ and **R**_**2**_ are −H, poor solubility of the zinc complex was observed in the EGME and PGME solvents. However, when either **R**_**1**_ or **R**_**2**_ was replaced with −CH_3_ group, and keeping the other as −H group, a slight improvement in solubility was observed but still not sufficient to form a film. With both **R**_**1**_ and **R**_**2**_ as −CH_3_ groups, the zinc oximate complex (diaquabis[2-(methoxyimino)propanoato]zinc(II), Zn(CH_3_ONCCH_3_COO)_2_·2H_2_O, hereafter referred to as ZnMIP_2_, where MIP stands for methoxyiminopropanoate group) showed high solubility in water, methanol, ethanol, isopropyl alcohol, EGME, and PGME. Crucially, smooth spin-coated films were obtained when EGME or PGME was used as the base solvent for ZnMIP_2_. Similarly, zinc-based oximate complexes with bulkier organic groups at **R**_**1**_ and **R**_**2**_ were prepared as shown in Figure 1a. They all showed good solubility in organic solvents and had good film formability. Considering the lower atom economy of these resists, they were not considered for further testing. The anhydrous version of ZnMIP_2_ has a molecular mass of 297.58 Da and 28 atoms. On the other hand, the as-prepared hydrated version of this complex has a molecular mass of 333.61 Da and 34 atoms.

The ZnMIP_2_ resist was characterized using Fourier-transform infrared spectroscopy (FTIR) and thermogravimetry evolved gas analysis (TG-EGA) performed via mass spectrometry (TG-EGA-MS or simply TGA-MS). The FTIR spectrum of the ZnMIP_2_ resist shows characteristic absorption bands between 2000 and 600 cm^–1^ (Figure 1b). The absorption bands at 1641 cm^–1^ (C=N), 1599 cm^–1^ [asymmetric vibrations of ν(COO)], 1395 cm^–1^ (symmetric bend of −CH_3_), 1366 cm^–1^ [symmetric vibrations of ν(COO)], 1050 cm^–1^ (N–O), and 866 cm^–1^ (−OCO– in plane) could be assigned unambiguously.^47^ The presence of symmetric and asymmetric ν(COO) bands suggests the possibility of π-delocalization of the C–O bond.

TGA-MS of ZnMIP_2_ in a helium atmosphere shows an initial mass loss of 10.7% (theoretical loss: 10.8%) between 50 and 82 °C that can be attributed to the loss of two water molecules (H_2_O, *m*/*z*^+^ 18) from the compound (Figure 1c). Further heating results in the molecular rearrangement via second-order type Beckmann decomposition reaction,^48^ resulting in a constant experimental mass of 24.4% that corresponds very well with the theoretical ceramic yield of ZnO (28%). Mass spectrometry reveals that between 120 and 250 °C, there is an almost simultaneous evolution of compounds such as acetonitrile (CH_3_CN, *m*/*z*^+^ 41), methanol (CH_3_OH, *m*/*z*^+^ 32), carbon dioxide (CO_2_, *m*/*z*^+^ 44), and water (H_2_O, *m*/*z*^+^ 18). Their yields reach a peak at 167 °C. Our TGA-MS results are consistent with what has been observed before with this compound.^49^ Furthermore, these results from ZnMIP_2_ tally well with the fact that various α-oximino acid molecules, **R**_**2**_–C(=N–O**R**_**1**_)COOH, decompose into **R**_**2**_CN, **R**_**1**_OH, and CO_2_ at temperatures close to 150 °C,^44^ again via second-order Beckmann fragmentation or Beckmann fission.^50,51^ Their low thermal stability is due to the presence of reactive N–O bonds, as well as the repulsion between the lone-pair electrons of its adjacent N and O atoms that leads to the easy fracture of the N–O bond.

### 2.2 Mechanistic Unity in Resist Exposure Mechanism in the ΨMORE^2^ Platform for EBL and EUVL

**Figure 2:**
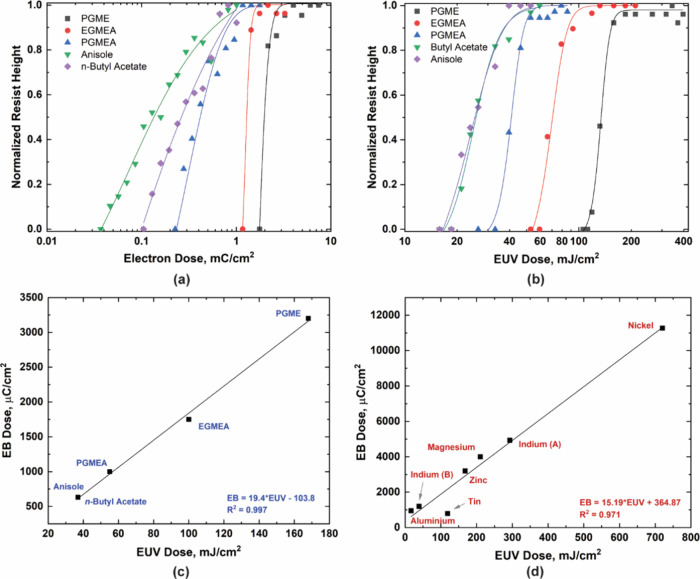
Contrast curves of the ZnMIP_2_ resist derived from (a) EBL and (b) EUVL for different developers. The original thickness of the resist for dose test measurements was 48–50 nm. (c) For the ZnMIP_2_ resist, the relationship between EB and EUV doses (at 100% normalized thickness) for different developers. (d) Relationship between EB and EUV doses (at 100% normalized thickness) for different metal-containing resists when PGME was used as a developer. Akin to ZnMIP_2_, the central metal atom in these resists has the same or similar chemical environment (as in indium (A)) around it. The tin-containing resist is an outlier here due to its chemical instability leading to uncertainty in postexposure thickness measurement.

The sensitivity and contrast of the ZnMIP_2_ molecular resist were evaluated using EBL (100 kV) and EUVL. Five different solvents were used as developers. The heights of the patterned resist exposed at different doses were measured using a profilometer. The normalized resist height versus dose was plotted to evaluate the sensitivity and contrast of the resist. The sensitivity of a resist is the exposure dose that provides a thickness of the remaining film equal to 50% of the original value. Because ZnMIP_2_ exhibits a negative tone behavior, its contrast is defined as γ = |log(*D*_100_/*D*_0_)|^–1^, where *D*_0_ and *D*_100_ correspond to electron or EUV doses at 0 and 100% of the remaining film thickness, respectively. Figure 2a,b shows the contrast curves (also called dose-to-gel curves) of the ZnMIP_2_ resist obtained using EBL and EUVL, respectively. The sensitivity and contrast values obtained using EBL and EUVL are given in Table Ia,b, respectively. Although PGME, ethylene glycol monomethyl ether acetate (EGMEA), and propylene glycol monomethyl ether acetate (PGMEA) all show high contrast in EUVL, PGMEA showed poor contrast in EBL. On the other hand, both anisole and *n*-butyl acetate developers showed comparable behavior in both EBL and EUVL.

**Table I:** Sensitivity and Contrast Values for Different Developers Obtained from (a) EBL and (b) EUVL of the ZnMIP_2_ Resist

**(a) developer**	**sensitivity (*D***_**50**_**),mC/cm**^**2**^	***D***_**100**_**,mC/cm**^**2**^	contrast, γ
PGME	1.98	3.2	9.0
EGMEA	1.29	1.74	10.5
PGMEA	0.42	1.06	1.9
*n*-butyl acetate	0.27	0.68	1.2
anisole	0.14	0.675	0.9

**(b) developer**	**sensitivity (*D***_**50**_**),**mJ/cm^2^	***D***_**100**_**,**mJ/cm^2^	contrast, γ
PGME	134	168	8.8
EGMEA	69	100	5.3
PGMEA	41	55	6.2
*n*-butyl acetate	25	37	3.2
anisole	25	37	2.7

Although the EBL and EUVL contrast curves for ZnMIP_2_ resist seem to bear no apparent relationship, the dose required to achieve 100% normalized resist thickness (i.e., *D*_100_, see Table I) for different developers using EUVL and EBL shows a linear correlation (Figure 2c and Figure S5d in the Supporting Information for other developers). Such a correlation is also seen for other metal-containing resists belonging to the ΨMORE^2^ platform (Figures S6d, S7d, S8d, and S9d in the Supporting Information). This implies that the developers are encountering the same or similar type of chemical product in an exposed ZnMIP_2_ resist induced by two different ionizing sources. In other words, this means that both EBL and EUV exposures lead to the same or similar chemical changes in the resist that lead to the solubility switch in the developers. Broadly speaking, the dose required to achieve 100% normalized resist thickness for different developers using EUVL and EBL seems to follow the trend in polarity and hydrogen bonding in the solvents as indicated by their respective Hansen solubility parameters (see Supplementary Discussion 8 and Figure S13a,b).^52^ Interestingly, such a linear correlation between EUV and EBL doses for different developers also provides an effective way to estimate the EUV sensitivity of a metal-containing resist from the ΨMORE^2^ platform for a given developer if its electron beam sensitivity is known. Crucially, the linear correlation for different developers using EUVL and EBL was also found to be valid for other metal oximate complexes.

What happens when we zoom out and take a global view of the relationship between EBL and EUVL for various metal-containing resists belonging to the ΨMORE^2^ resist platform? In Figure 2d, we see that there exists a linear correlation between EB and EUV doses for different metal-containing resists at 100% normalized thickness when PGME is used as a developer. The chemical environment around the central metal atom in these resists is the same (MIP groups) or similar (hydroxyiminopropanoate, HIP, groups as in indium (A)). Because different metal-containing resists show a linear relationship between EBL and EUVL for PGME developer, this suggests that PGME is encountering almost identical chemical change after exposure irrespective of the central metal atom. This implies that the exposure mechanisms in these resists must be similar as well. The parsimonious conclusion is that different central metal atoms are responsible for differences in sensitivities of the resists given that the chemical environment around each of the resists is the same or similar. Unsurprisingly, the linear correlation between EB and EUV doses is observed with other developers as well (see Supplementary Discussion 7 and Figure S12 in the Supporting Information). There are several observations that can be made from this result.

First, the EUV exposure dose needed to reach *D*_100_ for a metal-containing resist has no correlation with the theoretical EUV absorption cross section of the metal (Figure S14 in the Supporting Information). For example, zinc and nickel have very similar EUV absorption cross sections, but their resists sit widely apart when it comes to the dose required to pattern. Interestingly, the electron dose follows the trend observed in EUV dose. On the other hand, both aluminum and magnesium have similar EUV absorption cross sections, which are smaller than those of zinc and nickel. Yet they show better or comparable sensitivity with that of zinc. We speculate that the exposure mechanism of metal-containing resist and its sensitivity in the ΨMORE^2^ platform are dependent on the number of secondary electrons generated after the absorption of a EUV photon and perhaps their corresponding energies.

Second, the EB and EUV sensitivity of a metal-containing resist is highly dependent on the chemical environment around it. Indium-containing resists provide the best demonstration of this principle (Figure 2d). Indium (B) has both **R**_**1**_ and **R**_**2**_ as −CH_3_ groups whereas indium (A) has **R**_**1**_ as −H and **R**_**2**_ as −CH_3_ group. In other words, the sensitivity of a metal-containing oximate complex to electrons or EUV photons can be modulated by varying the O substituent of the amine.

Third, it provides an elegant way to estimate the EUV sensitivity of an untested metal-containing resist from the ΨMORE^2^ platform if its EB sensitivity is known.

In the scientific literature, attempts to derive EUV sensitivity of a metal-containing resist using the data from EBL are nonexistent. For organic resists, Oyama et al. demonstrated that for main-chain scission-type positive tone nonchemically amplified (PMMA, ZEP520A and ZEP7000) and chemically amplified (OEBR-CAP112) resists, it is possible to predict the EUV sensitivity of the resist using the sensitivity data from conventional EBL.^53^ They used different solvents as developers for each of these resists and assumed that the chemical reactions induced by the two ionizing sources are the same. Our work convincingly demonstrates that there exists a mechanistic unity between EBL and EUVL for the metal-containing resists based on the ΨMORE^2^ platform. The existence of a linear relationship between EB and EUV doses for a single metal-containing resist with different developers (Figure 2c) and different metal-containing resists with a single developer (Figure 2d) enables us to predict the EUV dose required for exposure of a metal-containing resist using an arbitrary developer or an arbitrary metal-containing resist using a developer if the corresponding EB dose is known.

### 2.3 Oximate Ligand as the Radical Initiator in EUVL and EBL

**Figure 3:**
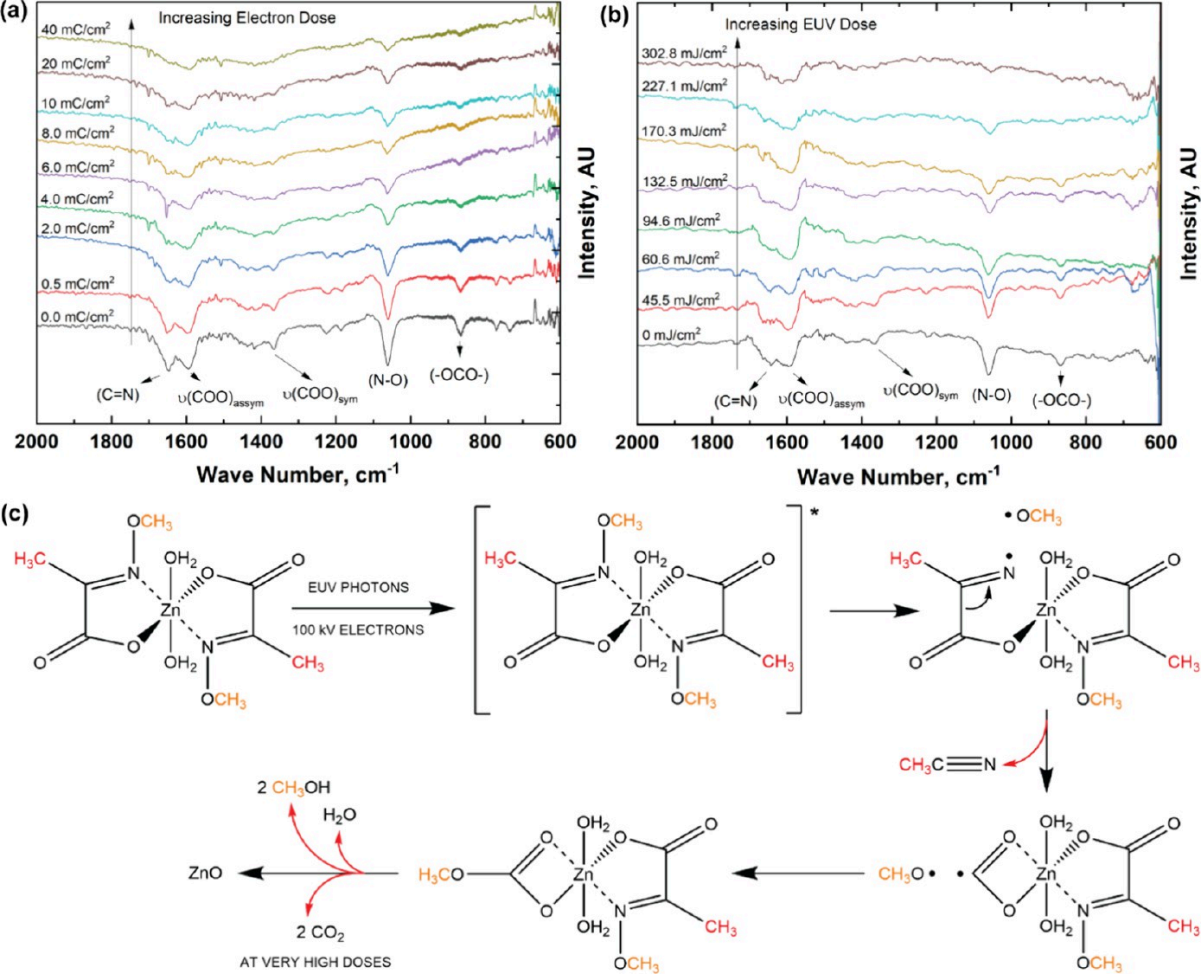
Micro-FTIR spectra of dose-dependent changes in the ZnMIP_2_ resist when exposed to (a) a 100 kV electron beam and (b) ≅92 eV EUV photons. (c) Tentative exposure mechanism of the ZnMIP_2_ resist when exposed to a beam of energetic electrons or EUV photons. Here only a single oximate ligand is shown to undergo fragmentation, as the solubility switch requires loss of only a fraction of all ligands. Understandably, at very high doses, both of the ligands could fragment.

Earlier, we found a direct correlation between the amount of EB and EUV dose needed to fully develop the resist for different developers. Furthermore, it was implied that the developers must be encountering the same or similar type of chemical product in an exposed ZnMIP_2_ resist generated by two different ionizing sources. Micro-FTIR was used to understand the dose-dependent chemical changes happening in the ZnMIP_2_ resist when exposed to a beam of electrons and EUV photons (Figure 3a,b). In both cases, the micro-FTIR spectra show that the bands associated with (C=N) and (N–O) bonds undergo a reduction in intensity with increasing dose, suggesting the radiolysis of the ZnMIP_2_ molecule. Given the striking similarities between the micro-FTIR spectra of EB- and EUV-exposed samples, we strongly speculate that both forms of ionizing irradiations of the resist ultimately result in similar chemical changes leading to similar end-products. In other words, a developer sees almost the same chemical environment in the exposed resist irrespective of whether EB or EUV is used for patterning. This is not surprising as, in both EB and EUV, it is the secondary electrons generated from primary electrons of EB and EUV photons, respectively, that are responsible for inducing the chemical changes in the resist via the loss of organic moieties, leading to its solubility switch. Furthermore, on a fundamental level, it tacitly suggests that the mechanism of initiation of the radiolysis process in EBL and EUVL must be the same. To understand this, let us turn our attention to how the oximate ligand acts as the radical initiator in both these lithographies.

Oximes and oxime esters have been extensively studied as photoinitiators for the last two decades.^54−59^ Irradiation of these molecules leads to the homolytic cleavage of the N–O bond, generating iminyl and acyloxy radicals.^54,57^ The acyloxy radical undergoes a decarboxylation process, which results in the formation of CO_2_ and an active radical. These radicals are then used to initiate photopolymerization in resins. Likewise, in our case, the oximate ligand, **CH**_**3**_–C(=N–O**CH**_**3**_)COO^–^, attached to zinc plays the crucial role of radical initiator. The difference here is that the production of active radicals does not initiate polymerization; instead, they lead to fragmentation of the oximate molecule. Exposure of the ZnMIP_2_ resist to EB and EUV photons generates secondary electrons that lead to the cleavage of N–O bond in oximate, thus producing N- and O-centered radicals. Based on the micro-FTIR (Figure 3a,b) and TGA-MS data (Figure 1c), we tentatively propose a second-order Beckmann fragmentation mechanism as the pathway of chemical changes happening in the ZnMIP_2_ resist during irradiation (Figure 3c). Semiquantitative data from micro-FTIR (Figure 3a,b) show that the intensity of the (C=N) band reduces faster than the one associated with asymmetric vibrations of ν(COO). This suggests that the formation of N- and O-centered radicals ensues in an intramolecular rearrangement *via* β-scission leading to the formation of a volatile nitrile (CH_3_CN) that is quickly eliminated via the vacuum system. Concurrently, the formation of a metastable zinc complex with monomethylcarbonate ligands takes place.^60^ Previous experimental studies and *ab initio* calculations for the thermal stability of dialkylcarbonates suggest the formation of corresponding alcohols and olefins, but the formation of ethers is energetically not favorable.^49,61,62^ Thus, with a further increase in dose, the metastable zinc complex with monomethylcarbonate ligands decomposes with the evolution of alcohol and CO_2_.

Although our proposed model of exposure mechanism mimics the well-established oxime and oxime ester photochemistry,^54^ it deviates from it on a crucial point: the radicals that are generated initiate fragmentation of the oximate molecule itself. Radicals initiating depolymerization or fragmentation of a resist are not unprecedented. More than 25 years ago, Willson et al. demonstrated that radicals generated during DUV exposure can be used to depolymerize a resist instead of cross-linking it.^63^ In our case, the N-centered radical, i.e., the −C=N• radical (Figure 3c), initiates its own fragmentation via intramolecular rearrangement to release a nitrile. Once that happens, the resist molecule irreversibly collapses, leading to its solubility switch in developers.

Comparing doses needed to pattern the resist in Figure 2a,b with the micro-FTIR in Figure 3a,b, it is interesting to note that only a few resist molecules need to undergo radiolysis to enable the solubility switch in all of the developers studied here. Probabilistically, it is possible that in the bulk of the film, some individual complexes lose only a single ligand, some lose both ligands, and some do not fragment at all during the exposure, and their collective effect leads to the solubility switch. This is reflected in the fact that, during the exposure, the resist loses ∼45% of the original thickness, leading to a plateau in the contrast curves at and above *D*_100_. This thickness loss corresponds to the use of PGME, a strong and aggressive developer. Because the spin-coated resist was not baked before exposure, the loss of ∼45% of resist thickness also includes the collapse of the empty space in the resist. Baking the spin-coated resist at 80 and 90 °C for 60 s leads to thickness shrinkage by ∼7.5 and ∼9%, respectively. In other words, the actual thickness loss of resist due to the loss of organic moieties during exposure is smaller than ∼45%, somewhat closer to ∼35% depending upon the baking temperature. So, the retained film thickness after exposure is ∼65% of the original value. As for weaker developers such as anisole and *n*-butyl acetate, slightly higher film thicknesses are retained, which are just over ∼70% of the original value. For EUVL, this is significant as it suggests high atom economy of the resist. A further increase in dose leads to a further reduction in thickness due to the continued radiolysis of organic moieties (Figures S5–S11 in the Supporting Information).

The juxtaposition of metal and radical initiator provides the shortest pathway for chemical changes in the resist molecule during exposure to energetic electrons or EUV photons. Secondary electrons generated by the metal center after absorbing EUV photons drive the fragmentation of the radical initiator attached to the metal atom. On the other hand, the metal atom participates in the intramolecular rearrangement to temporarily “stabilize” whatever is left of the molecule. Any further increase in photon dose leads to the production of more secondary electrons by the metal atom, which in turn results in further breakdown of the organic moiety, ideally ending up as metal oxide. In other words, the metal atom attached to the radical initiator plays a paradoxical role in the photochemical progression of the resist.

We are now left with the question of how the sensitivity of a metal-containing oximate complex to energetic electrons or EUV photons can be modulated by varying the O substituent of the amine. To answer this question, let us consider indium (A) oximate and indium (B) oximate resists (Figure 2d). Although the skeletal structure of both of the indium oximate complexes is the same, replacing a single group leads to profound changes in the sensitivity and solubility of the resist. Because an oximate ion, **R**_**2**_–C(=N–O**R**_**1**_)COO^–^, undergoes the proposed second-order Beckmann fragmentation to give a nitrile, the strength of the N–O bond in **R**_**2**_–C(=N–O**R**_**1**_)COO^–^ determines how easily the nitrile can be released for this reaction to proceed. The N–O bonds in oximes and oximates are usually weak.^64,65^ The N–O bond dissociation energies are 61.3 and 55.7 kcal/mol in hydroxylamine and *O*-methoxyamine, respectively;^66,67^ 48–50 kcal/mol in oxime esters;^58^ and only 33–37 kcal/mol in *O*-phenyl oxime ethers.^68^ Because hydroxylamine and *O*-methoxyamine form part of the backbone in indium (A) oximate and indium (B) oximate, the energy needed to break each N–O bond in these compounds is 2.65 and 2.41 eV, respectively, assuming that the N–O bond dissociation energies remain more or less constant in their respective oximate complexes. This is reflected in a larger dose needed to expose indium (A) oximate resist as compared to indium (B) oximate using either an EB or EUV photon (Figure 2d). Homolytic scission of N–O bonds can be effectuated either by thermal means (Figure 1c) or, as in our case, by secondary electrons generated from primary electrons of EB/EUV photon exposure (Figure 3a,b). This results in the formation of an N-centered radical with a simultaneous generation of one equivalent of its O-centered counterpart. Generally, factors that increase the resonance stabilization of these two radicals formed on dissociation of the N–O bond will lead to a decrease in the bond dissociation energy and hence weakening of the N–O bond. Because the **•**OH radical is less stable than **•**OCH_3_ radical,^69^ it is expected that the N–O bond in **CH**_**3**_–C(=N–O**H**)COO^–^ in indium (A) oximate is stronger than that in **CH**_**3**_–C(=N–O**CH**_**3**_)COO^–^ in indium (B) oximate. Thus, the stability of radicals is directly related to the N–O bond dissociation energy.

### 2.4 Electron Beam Lithography of the ZnMIP_2_ Resist: Approaching Angstrom-Scale Resolution

**Figure 4:**
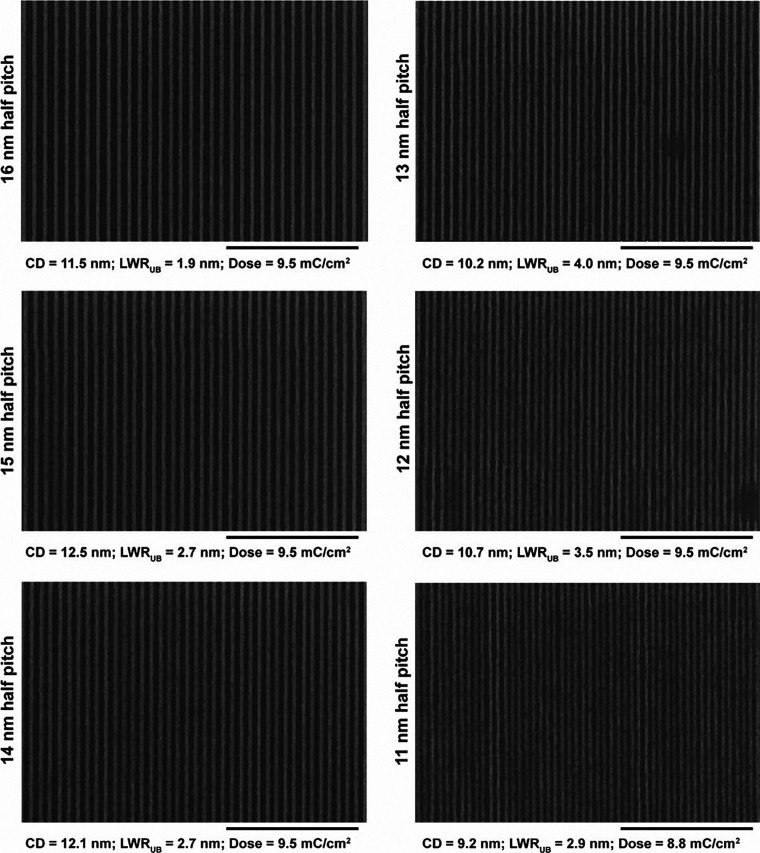
Composite SEM image of EBL of the ZnMIP_2_ resist with a designed pattern duty cycle of 1:1 at different half-pitches from 16 to 11 nm. PGME was used as the developer. Notice the slow appearance of scum between the lines from 14 nm half-pitch onward due to the proximity effect. Scumming progressively worsens with the reduction of pitch, and at 11 nm half-pitch, the scum almost envelopes the patterns. The height of patterns is 13 nm. Scale bar indicates 400 nm.

High-resolution patterning of ZnMIP_2_ was carried out using a Vistec EBPG 5000Plus 100 kV electron beam writer operating with a probe current of 1 nA and a beam step size of 1 nm. To assess the resolution achievable with equal line and spaces (i.e., 1:1 duty cycle), an approximately 9 × 9 μm area containing lines from 16 to 3 nm half-pitch, at 1 nm increments, were patterned using EBL (Figure S15 in the Supporting Information). The reason for the large area patterning was to check on the reproducibility and the onset of the proximity effect and to identify defects. Exposed samples were developed in PGME, PGMEA, anisole, and *n*-butyl acetate. Samples developed in low-contrast developers (Figure 2a; Table Ia) such as anisole and *n*-butyl acetate showed extensive scum at 16 nm half-pitch (Figure S16 in the Supporting Information). A low-contrast and high-sensitivity developer starts seeing solubility changes for only a small degradation of organic moieties after the exposure of the resist. Here, the areas affected by the proximity effect manifest themselves as scum. With PGMEA, a developer with slightly better contrast, the scum was greatly reduced (Figure S17 in the Supporting Information). The best results were obtained with PGME, a low-sensitivity yet high-contrast developer. With PGME, a higher dose is needed to degrade enough organic moieties in the resist, leading to high contrast (as areas with small chemical changes are still dissolved). Figure 4 shows well-resolved lines of 1:1 duty cycle from 16 to 11 nm half-pitch. Because of the increased proximity effect, with decreasing pitch, the amount of scum gradually increases and almost envelopes the patterns at 11 nm half-pitch. This is also reflected in the unbiased line-width roughness (LWR_UB_) value that is the lowest for 16 nm half-pitch (LWR_UB_ = 1.9 nm) that shows no proximity effect. It gradually worsens as the pitches get shorter.

**Figure 5:**
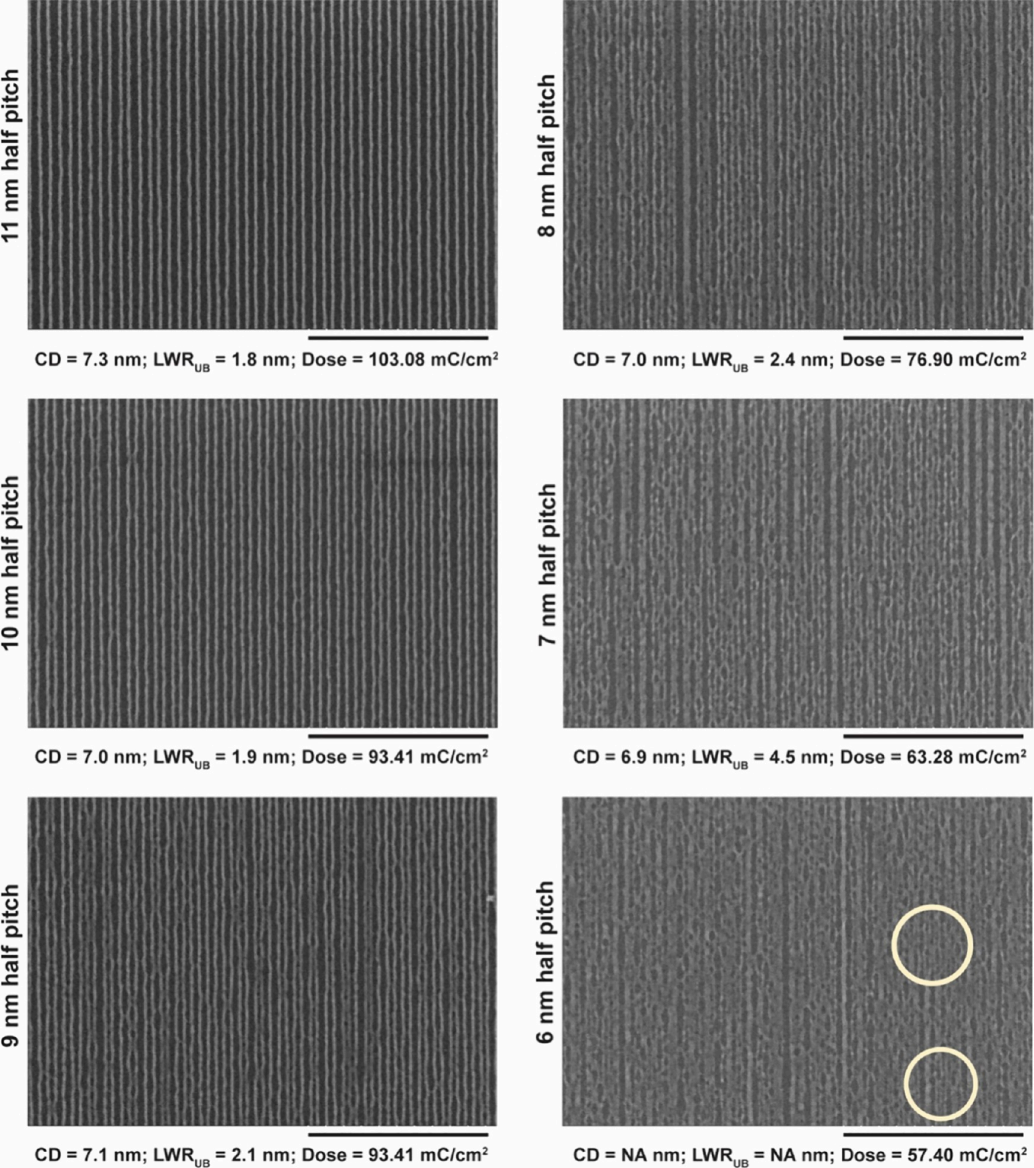
Composite SEM image of EBL of single pass lines using the ZnMIP_2_ resist. Because of reduced pattern density, the proximity effect is nearly absent, and that enables patterning of lines down to 7 nm half-pitch. Below this pitch, there are increased merging of patterns and pattern collapse. For the 6 nm half-pitch, the circled areas show the presence of lines that have not collapsed. The height of patterns is 10–11 nm. PGME was used as the developer. Scale bar indicates 400 nm.

When the duty cycle is reduced by patterning single pass lines from 11 nm half-pitch onward (Figure 5), the LWR_UB_ goes back to a low value of 1.8 nm due to the substantially reduced proximity effect (as observed for the 16 nm half-pitch in Figure 4). This LWR_UB_ remains nearly constant until the 9 nm half-pitch. From the 8 nm half-pitch and below, the LWR_UB_ progressively worsens as the critical dimension of the lines (∼7 nm) starts to match the half-pitch, making them wiggle, merge, and/or collapse. Unsurprisingly, at 6 nm half-pitch, almost all the lines have either merged or collapsed. There are, however, pockets of isolated features where 6 nm half-pitch lines could be seen (circled areas in Figure 5). This work has demonstrated clearly delineated sub-10 nm half-pitch EBL over a large area. Earlier attempts demonstrated 7 nm half-pitch nested-L test structures on a very small area using a hydrogen silsesquioxane resist.^70^

To get a better understanding of the proximity effect imparted by the 100 kV electron beam, we performed EBL simulations using the embedded 2D e-beam module of the exposure simulation and pattern preparation software BEAMER (GenISys GmbH) using Monte Carlo simulated point spread function (PSF) (see Supplementary Discussion 13 and Figure S18 in the Supporting Information). The results of 2D e-beam simulations on 1:1 line/space patterns are depicted in Figure S20 (Supporting Information). A gradient in the EB dose at the exposure boundaries can be seen in all cases. As the half-pitch line width decreases from 16 to 11 nm, the exposure dose received at the spaces in-between the exposed regions can be seen to increase. This effect is more clearly seen in the cross-sectional relative energy plots depicted in Figure S21 (Supporting Information), where the EB dose fraction received by the space in between the exposed lines increases with a decrease in half-pitch. This leads to the formation of resist scum between the desired features and negatively affects the LWR_UB_. When the duty cycle of the exposures is modified by exposing single pass lines (Figures S22 and S23 in the Supporting Information), the relative dose received at the spaces between the lines is drastically reduced to a negligible amount at first. This helps in the formation of cleanly delineated lines from half-pitch 11 to 9 nm. However, a further decrease in the half-pitch once again starts imparting significant exposure dose in between the lines, and resist scum starts to reappear while also compromising LWR_UB_.

**Figure 6:**
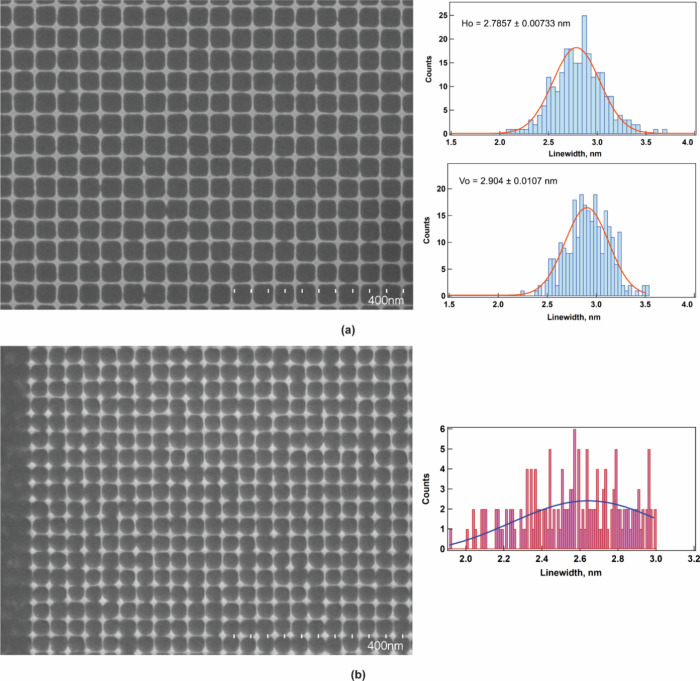
SEM image of EBL of sub-3 nm single pass lines written as a crossbar structure at an electron dose of (a) 47.9 mC/cm^2^ and (b) 38.0 mC/cm^2^. Their corresponding line-width analysis is shown on the right-hand side. *H*_o_ and *V*_o_ in panel a correspond to average line-widths of horizontal and vertical lines, respectively.

Because ZnMIP_2_ is a low-molecular-mass resist material, a worthwhile question to ask is what is the highest possible resolution achievable with this resist using EBL. To answer this question, we exposed single pass lines in the form of a crossbar structure at incremental doses. Crossbar structures provide rigidity to the lines, suppress their collapse, and enable understanding of the progression of line-widths with increasing dose. Figure 6a shows single pass lines exposed at a dose of 47.9 mC/cm^2^ at which they all were able to stand. The Multipeak Fit package in the Igor Pro 9 plotting software was used to analyze their critical dimensions and uniformity. From the SEM image, line profiles of the pattern were extracted, a Gaussian profile was fitted, and finally, the widths of the peaks at full width at half-maximum (fwhm) were obtained. The average line-widths of horizontal and vertical lines were found to be 2.8 and 2.9 nm, respectively, and both of them exhibit a narrow spread. Furthermore, there are plenty of lines that have feature widths 2.5 nm or below. From the collapsed lines, the measured aspect ratio was ∼8 (Figure S24 in the Supporting Information). Increasing the dose to 51.7 mC/cm^2^ barely increases the average line-widths in the horizontal and vertical direction (2.9 and 3.1 nm, respectively) with features below 2.5 nm still discernible (Figure S25 in the Supporting Information). However, when the dose is reduced to a value of 38.0 mC/cm^2^, the lines are just barely standing (Figure 6b); for analysis, the lines had to be handpicked due to the software not being able to detect them automatically. Here the average line width was reduced to ∼2.6 nm. On other hand, the minimum line-width that could be detected was 1.9–2.0 nm. These are the smallest lines ever fabricated by using a commercial EBL machine.

It is widely recognized that the smallest feature size that can be patterned with EBL is about the size of the beam itself.^71,72^ In our case, a (theoretical) probe size of 5 nm (Figure S26 in the Supporting Information) was used to pattern a line-width of 2.8 nm using a ZnMIP_2_ resist. This result goes against the established norms in EBL. Interestingly, this phenomenon was also observed with the NiMIP_2_ resist where the measured patterned size was consistently smaller than the designed width (Figure S27 in the Supporting Information). When the percentage reduction in designed width is plotted against the designed width, an exponential relationship was observed: at larger designed widths, the patterned width showed a smaller deviation, whereas at smaller designed widths, the patterned width could be close to 50% of the designed width. The reason for its abnormal behavior is not well understood.

Previous studies have conclusively shown that removing the lift-off is crucial to achieving sub-10 nm resolution in the direct patterning of functional materials using conventional EBL.^14,73−75^Combining this with a low-molecular-mass resist of high atom economy and a fine electron probe, the achievable resolution can be pushed further approaching angstrom scale.

### 2.5 EUV Lithography of the ZnMIP_2_ Resist

We demonstrated that the resist exposure mechanism is similar to energetic electrons and EUV photons. However, in EBL, the backscattered electrons due to their long travel lengths can be responsible for resist exposure far from beam incidence (i.e., proximity effect), as backscattered electrons can generate secondary electrons along its path to expose the resist. The proximity effect is mostly present in highly dense and fine patterns, which can be a limiting factor in high-resolution EBL (see Figure 4; from 14 to 11 nm half-pitch). On the other hand, in EUV lithography, the absorbed photons generate low-energy secondary electrons that expose the resist. From the point where an EUV photon is absorbed by the resist matrix, these secondary electrons travel a distance of about ≈2.5 nm (“secondary electron blur”).^76−78^ A recent study suggests that it is between 1 and 2 nm, which is equal to the inelastic mean free path of electrons in the 20–92 eV range.^79^ Therefore, in EUVL, resist exposure due to the proximity effect is hardly observed. This means that the developers that give low contrast in EBL can be used for EUVL without concern for scum being left behind. In our case, we used PGMEA and anisole for the development of the exposed patterns.

**Figure 7:**
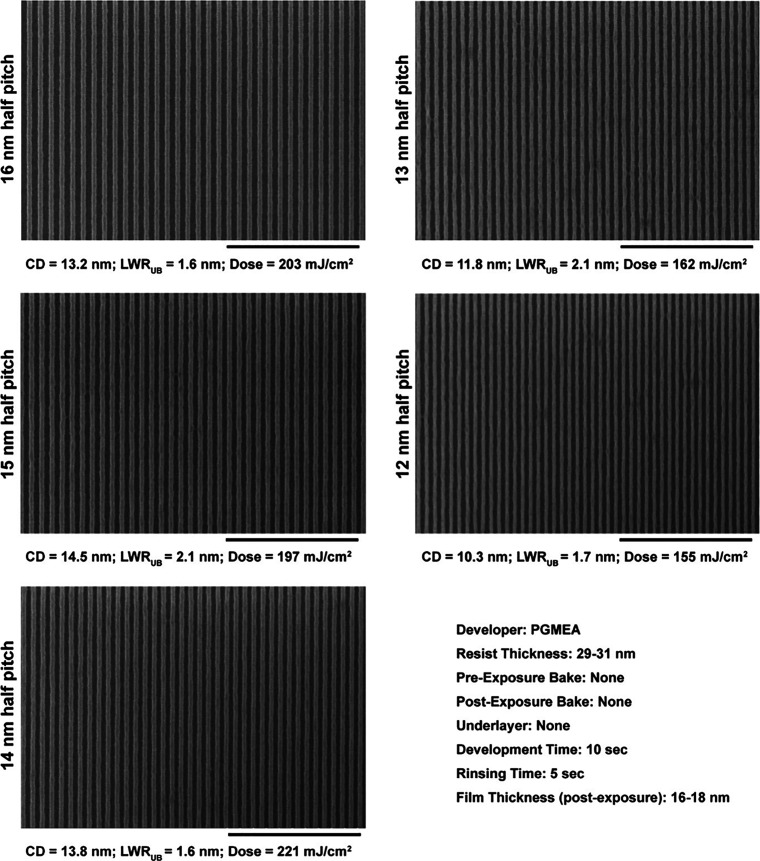
Composite SEM image of the ZnMIP_2_ resist patterned using EUV interference lithography showing half-pitches from 16 to 12 nm. Developed using PGMEA, the patterned features demonstrate not only high resolution but also very low unbiased line-width roughness, i.e., LWR_UB_ < 2 nm.

**Figure 8:**
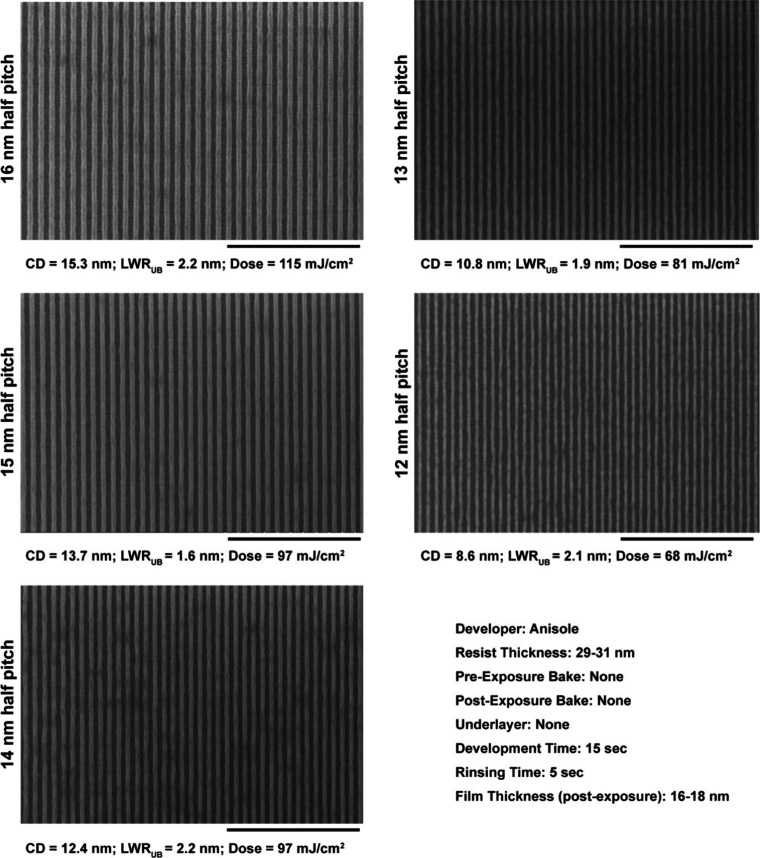
Composite SEM image of the ZnMIP_2_ resist patterned using EUV interference lithography showing half-pitches from 16 to 12 nm. Developed using anisole, the patterned features demonstrate not only high resolution but also a low LWR_UB_ closer to 2 nm.

Figure 7 shows the SEM images of the line/space patterns from 16 to 12 nm half-pitch developed using PGMEA, a high-contrast developer. The patterning was achieved on a bare silicon wafer without the addition of any underlayer and pre- or post-exposure bakes. Even under these bare minimum conditions of lithography, well-resolved line patterns were obtained with very low unbiased line-width roughness, i.e., LWR_UB_ < 2 nm. Unsurprisingly, the LWR_UB_ seen here corresponds very well with the observations made in EBL (Figures 4 and 5). Developing in a low-contrast solvent such as anisole lowers the dose-to-size requirement by approximately 50%, bringing it below 100 mJ/cm^2^, with a concomitant miniscule rise in LWR_UB_ to ≈2.0 nm as shown in Figure 8.

The LWR in EUVL comes from the collective contribution of the secondary electron blur, photon shot noise, deprotection blur, acid diffusion blur, resist swelling, and chemical contrast.^80−82^ For ZnMIP_2_, a monodisperse low-molecular-mass resist, the cumulative LWR contribution comes from the secondary electron blur, photon shot noise, and chemical contrast. During exposure of the ZnMIP_2_ resist, there is molecular fragmentation that leads to the removal of volatile nitrile, and the remaining species undergo intramolecular rearrangement that further reduces the molecular mass. This not only improves resolution but also contributes to surface minimization, leading to a smoother line edge and lower LWR_UB_. Furthermore, solvent contrast also affects the final LWR.

The *Z* factor is a performance trade-off metric that is used in EUVL to determine the efficacy of a resist. To stack up our results against the industry standard resist platforms, we calculated the *Z* factor based on the EUVL results obtained with both PGMEA and anisole developers (Figures 7 and 8) using the following expression:^83,84^
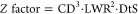
where CD is the critical dimension, LWR is the line-width roughness, and DtS is dose-to-size. The results are summarized in Table S13 in the Supporting Information.

**Figure 9:**
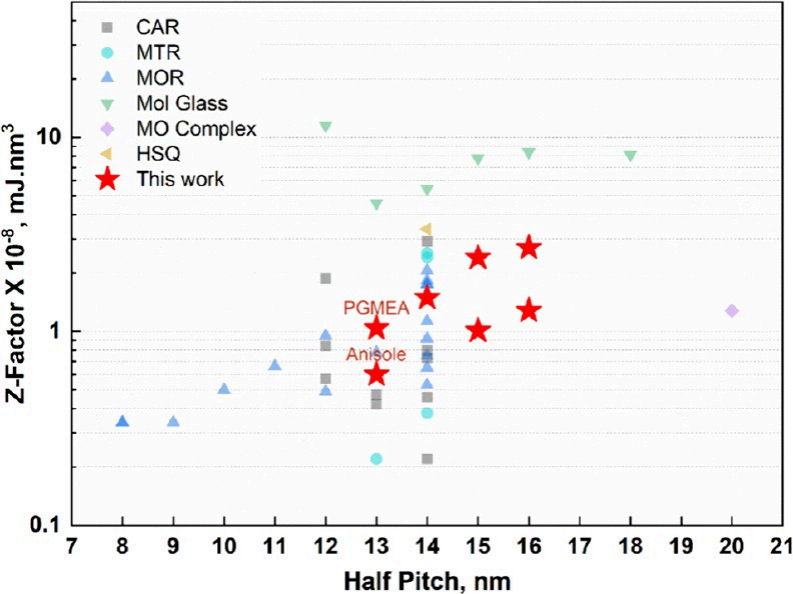
Benchmarking comparison of the ZnMIP_2_ resist with the state-of-the-art commercial and academic resist platforms depicting estimated *Z* factors against the corresponding reported resolution, classified by widely recognized resist categories. The detailed summary of the resist performance is provided in Tables S13 and S14 in the Supporting Information.^41,85−96^

Typically, lower *Z* factor values are desirable for a given resist to be suitable for high-volume manufacturing. To benchmark the ZnMIP_2_ resist compared with the state of the art, we have summarized recent reports on commercial and academic resist platforms in Table S14 in the Supporting Information, and the ZnMIP_2_ resist fairs very well. To easily visualize this comparison, we have illustrated estimated *Z* factors against the corresponding half-pitch resolution reported (Figure 9). Similar to our case, most mature resist platforms have reported resolution between 12 and 14 nm half-pitch, and our *Z* factor values fit right within the spread of reported values. Particularly, the *Z* factor of 0.6 × 10^–8^ mJ·nm^3^ for the 13 nm half-pitch developed with anisole is clearly comparable to chemically amplified resists (CARs) and metal-oxide resists (MORs) and inferior only to the multitriggered resist (MTR) platform (0.22 × 10^–8^ mJ·nm^3^). For the 12 nm half-pitch patterning using a ZnMIP_2_ resist, a patterned line-width smaller than 12 nm was obtained over the course of exposure doses used. Therefore, dose-to-size and *Z* factor could not be estimated. Note that the 11 nm half-pitch and below patterning was not tested for ZnMIP_2_ due to the instrument limitations.

As the microelectronics industry continues the extreme downscaling of semiconductor devices, the minimum patterned feature size is expected to reach 10 nm half-pitch by 2025 for 2.1 nm logic node (Intel’s 18 Å node), and even smaller feature sizes would be required going forward.^97^ Because of the inherent quantum nature of EUV photons, stochastic variations leading to defects and roughness are a persistent critical challenge. To overcome photon shot noise induced roughness, higher exposure doses become necessary, which in turn reduce throughput. Thus, resists exhibiting the required sub-10 nm half-pitch resolution with low LWR at low exposure doses remain to be the Holy Grail.^98,99^ To that end, our resist platform demonstrates EUVL results at par with state-of-the-art platforms with very low LWR values without requiring much process optimization. The evaluation of sub-12 nm half-pitch resolution was not possible due to limited experimental capabilities and remains the focus of future work. However, our EBL results fill in this gap by showing large-area patterning of sub-10 nm dense half-pitch lines and isolated features down to 2 nm, notably smaller than the electron beam size of 5 nm. With the beam size clearly being the resolution limiting factor here, increased access to advanced instrumentation possessing even smaller beam sizes is poised to exploit our resist platform for pushing the limits of lithography. Therefore, as the industry embarks into angstrom nodes and research in beyond EUVL at 6.5 nm wavelength picks up pace,^100^ we envisage our resist platform to be even capable of angstrom-scale pattern delineation as the instrument capabilities and access widen over the coming years.

## 3 Conclusions

In this work, we describe a low-molecular-mass metal-containing resist system (ΨMORE^2^) that is modular and versatile. This platform consists of a central metal atom bonded to a radical initiator. An oximate ligand serves as the radical initiator, the organic environment of which can be flexibly changed to improve characteristic requirements of a resist such as solubility in organic solvents, film formability, and sensitivity to energetic electrons and EUV photons. Because the principal aim is to keep the molecular size and mass as small as possible, the size of the organic environment here is restricted. We prepared and exposed various metal-containing oximate complexes (Zn, Ni, In, Al, Mg, and Sn) to energetic electrons and EUV photons and developed them using different developers. We found that there is a linear correlation between the EB dose and EUV dose needed to reach 100% normalized resist height (i.e., *D*_100_) when a single metal oximate is developed using different developers. This linear correlation also holds when a single developer was used to develop different metal complexes. This strongly suggests that there is a mechanistic unity between EBL and EUVL among the resists on the ΨMORE^2^ platform. Indeed, the micro-FTIR studies on ZnMIP_2_ resist show that the exposure mechanisms in both EBL and EUVL are almost identical, beginning with the loss of nitrile followed by intramolecular rearrangement. This results in the solubility switch of the ZnMIP_2_ resist giving it a negative tone behavior. We exploited this behavior to perform EBL and EUVL of the ZnMIP_2_ resist that yielded a very high resolution accompanied by very low unbiased line-width roughness (LWR_UB_ ≤ 2 nm). With EBL, dense single pass lines all the way down to 7 nm half-pitch over a large area were demonstrated. On the other hand, uniform isolated features as small as 2.8 nm were patterned. When underdosed, the average line width was reduced to ∼2.6 nm; the minimum patterned line-width detected was 1.9–2.0 nm. Currently, these are the smallest lines ever fabricated using a commercial EBL machine. Moreover, with EUVL, line/space patterns from 16 to 12 nm half-pitch were demonstrated with low *Z-*factor values comparable to mature commercial resist platforms. Here the dose-to-size requirement can be modulated by switching from a high-contrast developer to the one that shows slightly lower contrast.

Our study has conclusively demonstrated that low-molecular-mass metal-containing resists of high atom economy can achieve very high resolution and low LWR. Furthermore, for direct patterning of functional materials, metal-containing resists that do not need lift-off and etching steps are ideal candidates to achieve sub-10 nm resolution. With patterned features close to 1.9–2.0 nm and further room to improve the modular chemistry, the ΨMORE^2^ platform is well poised to reach angstrom-scale lithography with low-molecular-mass metal-containing resists.

## 4 Experimental Details

### 4.1 Resist Synthesis

The details of resists synthesized are given in the Supporting Information. For the sake of brevity, we describe here the synthesis of diaquabis[2-(methoxyimino)propanoato]zinc(II)) (ZnMIP_2_). It was synthesized using the known literature methods.^48,49,101^

Sodium pyruvate (3.30 g, 30 mM) was dissolved in 20 mL of deionized water. To this solution, methoxylamine hydrochloride (2.50 g, 30 mM) was added with vigorous stirring. The solution turned turbid. To this mixture, sodium bicarbonate (2.52 g, 30 mM) was added portionwise. The turbidity slowly disappeared with the continuing addition of sodium bicarbonate, and ultimately, the solution became clear. The reaction was allowed to continue until no visible gas evolution was seen. To the stirred solution was added zinc(II) nitrate hexahydrate (4.46 g, 15 mM). A white precipitate soon appeared. The stirring was continued for 18 h. Thereafter, it was filtered using a Büchner funnel, washed with a small amount of ice-cold water, and dried overnight in a vacuum oven. Yield = 2.4 g, 48%. Alkali metal-free synthesis is also possible by replacing sodium bicarbonate with ammonium bicarbonate and sodium pyruvate with pyruvic acid neutralized with ammonium bicarbonate.^102^

### 4.2 Characterization of Synthesized Resists

A Bruker Platinum ATR spectrometer was used to conduct Fourier transform infrared spectroscopy (FTIR) of all of the synthesized resists. Thermogravimetric analysis (TGA) was carried out with a Mettler Toledo TGA/SDTA 851e in the temperature range 25–600 °C (heating rate = 5 °C/min) in an argon atmosphere (10 mL/min). Concurrently, chemical analysis was carried out with Agilent 7700x inductively coupled plasma mass spectrometry (ICP-MS).

### 4.3 Preparation of Resists

Metal oximate complexes were dissolved in EGME or PGME to prepare their respective resists. They were filtered with a 0.1 μm pore PTFE filter before dispensing on a clean 4 in. silicon wafer. The conditions used for the preparation of resists and spin-coating are given below:

1. Dose test patterns: concentration = 0.025 g/mL; spin-coating speed = 1800 rpm.
2. Patterns for micro-FTIR studies: concentration = 0.025 g/mL; spin-coating speed = 1000 rpm.
3. High-resolution patterns: concentration = 0.0125 g/mL; spin-coating speed = 1800 rpm (EUVL); 2000 rpm (for 1:1 line/space features using EBL); 2500 rpm (for single-pass lines using EBL).
  Concentration = 0.025 g/mL; spin-coating speed = 1800 rpm (crossbar structures using EBL).

The resists were not baked before or after exposure to either electrons or photons.

### 4.4 Electron Beam Lithography (EBL)

Coated resists were exposed in a Vistec EBPG 5000Plus electron beam lithography tool operating at 100 kV. Following are the exposure conditions used for different studies:

1. Dose test patterns: size of the exposed pattern = 180 × 180 μm; beam current = 50 nA for doses from 19.2 to 122 μC/cm^2^; beam current = 100 nA for doses from 150 to 57,922 μC/cm^2^; beam step size = 25 nm.
2. Patterns for micro-FTIR studies: size of the exposed pattern = 180 × 180 μm; beam current = 100 nA for doses from 500 to 50,000 μC/cm^2^; beam step size = 25 nm.
3. High-resolution patterns: beam current = 1 nA; beam step size = 1 nm.

### 4.5 Extreme Ultraviolet Lithography (EUVL)

The EUV interference lithography (EUV-IL) setup commissioned at the XIL-II beamline of the Swiss Light Source synchrotron facility was used for this work. EUV-IL is a versatile tool for resist screening and provides flexibility in terms of outgassing and contamination. Because of its interferometric nature, the setup allows for nanopatterning of periodic features, such as lines/spaces (LS). More importantly, this tool provides a high-resolution and focus-independent aerial image with a pitch-independent contrast. Coherent synchrotron radiation of 13.5 nm wavelength was used in combination with diffraction gratings to form a high-resolution interference pattern that was used to pattern resists. For dose test measurements and micro-FTIR studies, an open-frame mask was used.

Monitoring of tool performance and metrology consistency was carried out routinely using a reference exposure. The principal sources of uncertainty include diffraction grating deterioration and beam-flux drift during exposure.

### 4.6 Characterization of Exposed Resists

A Veeco Dektak 8 stylus profilometer (vertical resolution close to 1 Å) was extensively used to measure the heights of samples exposed by using EBL and EUVL. For micro-FTIR measurements of samples exposed by using EBL and EUVL, a Bruker Hyperion 3000 FTIR microscope was used. Exposed patterns were inspected by using a scanning electron microscope (SEM, Hitachi Regulus 8230). The SEM parameters are based on industrial standard.^103^ Image analysis for measuring critical dimensions (CDs) and unbiased line width roughness (LWR_UB_) of patterns was carried out using SMILE, an in-house developed software.^104,105^

## Supporting Information Available

Chemicals; preparation of α-oximino acids; preparation of alkali metal salts of α-oximino acids; preparation of zinc salts of α-oximino acids; preparation of metals salts of α-oximino acids; EBL and EUVL contrast curves of different resists and their relationship; relationship between a developer and different metal-containing resists in EBL and EUVL; Hansen solubility parameters; relationship between EUV absorption cross section and EUV dose; patterning scheme adopted to demonstrate large area lithography; EBL and development of ZnMIP_2_ with anisole and *n*-butyl acetate; EBL and development of ZnMIP_2_ with PGMEA; simulation of the point spread function (PSF) in the ZnMIP_2_ resist; simulation of energy deposited on the resist during EBL; measurement of the height of lines and effect of increasing electron dose; simulated spot size of the electron beam in the Vistec EBPG 5000Plus EBL machine; designed width versus patterned width; *Z*-factor calculations; and benchmarking of resist performance (DOCX)
