# Supplementary material for: Approaching Angstrom-Scale Resolution in Lithography Using Low-Molecular-Mass Resists (<500 Da)
Source: ACS Nano. 2024 Aug 20;18(35):24076–94. doi: 10.1021/acsnano.4c03939 (PMC11375778; doi:10.1021/acsnano.4c03939)
Supplement: Supplementary file 1 — nn4c03939_si_001.docx [file nn4c03939_si_001.docx]

**SUPPORTING INFORMATION**

**Approaching Angstrom Scale Resolution in Lithography Using Low Molecular Mass Resists (<500 Da)**

Mohammad S. M. Saifullah,^1,2^^[[1]](#footnote-1)^* Anil Kumar Rajak,^1^ Kevin A. Hofhuis,^1^ Nikhil Tiwale,^3^ Zackaria Mahfoud,^4^ Andrea Testino,^1,5^ Prajith Karadan,^1^ Michaela Vockenhuber,^1^ Dimitrios Kazazis,^1^ Suresh Valiyaveettil,^6^ Yasin Ekinci^1*^

^1^ *Paul Scherrer Institut, Forschungsstrasse 111, 5232 Villigen PSI, Switzerland*

^2^ *PiBond Oy, Kutojantie 2B, 02630 Espoo, Finland*

^3^ *Center for Functional Nanomaterials, Brookhaven National Laboratory, Upton, NY 11973-5000, United States of America*

^4^ *Institute of Materials Research and Engineering, A*STAR (Agency for Science, Technology, and Research), 2 Fusionopolis Way, #08-03 Innovis, Singapore 138634, Republic of Singapore*

^5^ *École Polytechnique Fédérale de Lausanne, STI SMX-GE, CH 1015 Lausanne, Switzerland*

^6^ *Department of Chemistry, National University of Singapore, 3 Science Drive 3, Singapore 117543, Republic of Singapore*

**TABLE OF CONTENTS**

1. Chemicals
2. Preparation of α–Oximino Acids
3. Preparation of Alkali Metal Salts of α–Oximino Acids
4. Preparation of Zinc Salts of α–Oximino Acids
5. Preparation of Metals Salts of α–Oximino Acids
6. EBL and EUVL Contrast Curves of Different Resists and their Relationship
7. Relationship Between a Developer and Different Metal-containing Resists in EBL and EUVL
8. Hansen Solubility Parameters
9. Relationship Between EUV Absorption Cross-section and EUV Dose
10. Patterning Scheme Adopted to Demonstrate Large Area Lithography
11. EBL and Development of ZnMIP_2_ with Anisole and *n*-Butyl Acetate
12. EBL and Development of ZnMIP_2_ with PGMEA
13. Simulation of the Point Spread Function (PSF) in ZnMIP_2_ Resist
14. Simulation of Energy Deposited on the Resist During EBL
15. Measurement of Height of Lines and Effect of Increasing Electron Dose
16. Simulated Spot Size of Electron Beam in Vistec EBPG 5000Plus EBL Machine
17. Designed Width versus Patterned Width
18. Z–Factor Calculations
19. Benchmarking of Resist Performance

**1. Chemicals**

Following chemicals were purchased from the listed vendors. All the chemicals were used as received.

**Sigma Aldrich**: Methanol (99.8%); *iso*-Propanol (>99.5) Chloroform (>99%); Anisole (99%); *n*-Butyl acetate (>99.5%); Ethylene glycol monomethyl ether (EGME) or 2-methoxyethanol (>99.9%); Propylene glycol monomethyl ether (PGME) or 1-methoxy-2-propanol (>99.5%); Ethylene glycol butyl ether or 2-butoxyethanol (>99%); Ethylene glycol methyl ether acetate (EGMEA) or 2-methoxyethyl acetate (98%); Propylene glycol methyl ether acetate (PGMEA) (>99.5%); 3-Methoxy-1-butanol (99%).

Aluminum nitrate nonahydrate (99.99%); Magnesium sulfate (>97%); Indium(III) nitrate hydrate (>99.99%); Nickel acetate tetrahydrate (98%); Tin(II) chloride (98%); Zinc nitrate hexahydrate (98%); Sodium bicarbonate (>99.7%); Potassium carbonate (>98%).

Glyoxylic acid (98%); Pyruvic acid (98%); Sodium pyruvate (99%); Phenylglyoxylic acid (97%); Phenylpyruvic acid (98%); α–ketobutyric acid (97%), Sodium 3-methyl-2-oxobutyrate (95%); *O*–Methoxyamine hydrochloride (98%); *O*–Benzylhydroxylamine (99%); *O*–Allylhydroxylamine hydrochloride (>98%).

**TCI Chemicals Europe**: *O*–Ethylhydroxylamine hydrochloride (>98%); *O*–(*tert*-Butyl)hydroxylamine hydrochloride (>98%);

**Acros Organics**: Hydroxylamine hydrochloride (97%).

**2. Preparation of α–Oximino Acids**

These α–oximino acids were prepared according to literature methods or slight modifications of them.^1,2^

**Preparation of 2-(hydroxyimino)propanoic acid (HIPA), HONCCH_3_COOH, MW = 103.08**

Sodium pyruvate (6.6 g; 60 mM) was added to hydroxylamine hydrochloride (4.16 g; 60 mM) dissolved in 45 ml de-ionized water. The turbid solution formed was stirred for 1 hour. The mixture was acidified by dropwise addition of concentrated HCl to a pH value of 2. A white precipitate appeared. The mixture was spread on a petridish to evaporate the mixture to dryness. The dried powder was added to 50 ml acetone and was shaken for 2 minutes. The solution was filtered to remove insoluble impurities and the filtrate was evaporated to give the product. Yield = 5.95 g, 96%.

**Preparation of 2-(methoxyimino)propanoic acid (MIPA), CH_3_ONCCH_3_COOH, MW = 117.10**

Pyruvic acid (2.64 g or 2.08 ml; 30 mM) was added to methoxyamine hydrochloride (2.5 g; 30 mM) whilst stirring. The solution became translucent, and the slurry was cooled down to 5 °C in an ice bath. An aqueous solution of tetramethylammonium hydroxide (21.87 g of 25 wt% solution; 60 mM) was carefully added dropwise. A clear solution was obtained and allowed to stir for 2 hours. The mixture was acidified by dropwise addition of concentrated HCl to a pH value of 2. A white precipitate appeared. The mixture was cooled to 3 °C. The white precipitate was collected by Buchner funnel, washed with ice cold water, and dried in vacuum for 12 hours. Yield = 2.84 g, 81%.

**Preparation of 2-(ethylhydroxyimino)propanoic acid (EIPA), C_2_H_5_ONCCH_3_COOH, MW = 131.13**

Sodium pyruvate (3.3 g; 30 mM) was added to ethylhydroxylamine hydrochloride (2.92 g; 30 mM) dissolved in 20 ml de-ionized water whilst stirring. The solution turned turbid. Sodium bicarbonate (1.25 g; 15 mM) was added in small portions until the solution became clear and there was no visible gas evolution. The mixture was acidified by dropwise addition of concentrated HCl to a pH value of 2. A white precipitate appeared. The mixture was cooled to 3 °C. The white precipitate was collected by Buchner funnel, washed with ice cold water, and dried in vacuum for 12 hours. Yield = 3.18 g, 81.5%.

**FTIR Spectra of Prepared** **α–Oximino Acids**

Figure S1 shows the FTIR spectra of as prepared HIPA, MIPA, and EIPA. All the α–oximino acids show the presence of characteristics absorption bands associated with (C=N), (N–O), and ν(COO) that can be assigned unambiguously. These bands are tabulated in Table S1.


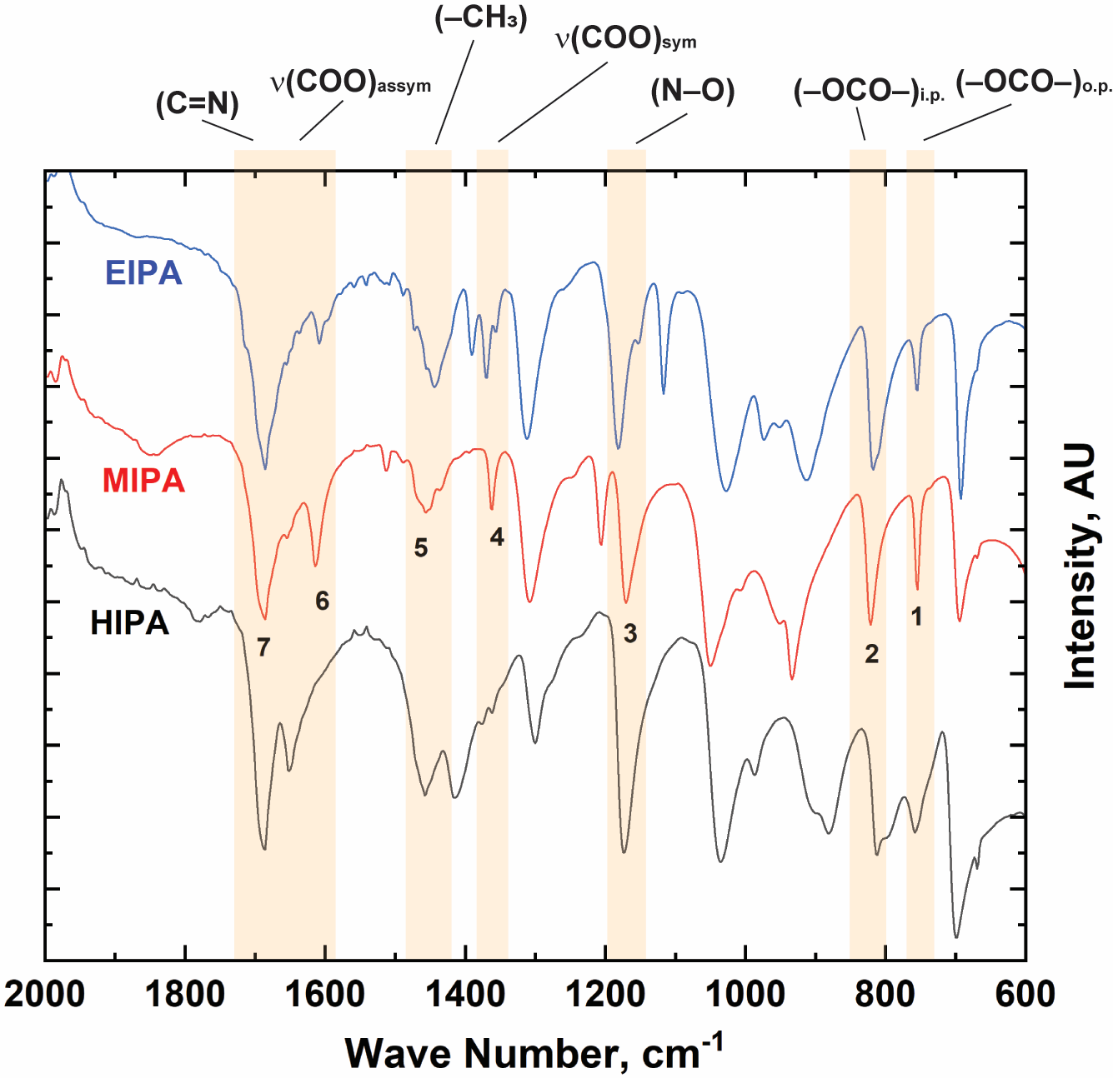


**Figure S1**: FTIR spectra of various α–oximino acids prepared in this study showing the characteristic absorption peaks associated with the oximate backbone (**R_2_**–C(=NO**R_1_**)COO). Assignment of peaks is shown in Table S1.

| **Number** | **Band, cm^-1^** | **HIPA** | **MIPA** | **EIPA** |
| --- | --- | --- | --- | --- |
| 1 | (–OCO–)_o.p._ | 757 | 754 | 755 |
| 2 | (–OCO–)_i.p._ | 812 | 820 | 818 |
| 3 | (N–O) | 1173 | 1170 | 1181 |
| 4 | ν(COO)_sym_ | 1414 | 1363 | 1370 |
| 5 | (–CH_3_) | 1457 | 1455 | 1444 |
| 6 | ν(COO)_assym_ | 1650 | 1613 | 1609 |
| 7 | (C=N) | 1687 | 1686 | 1686 |

**Table S1**: Characteristic absorption peaks of various α–oximino acids prepared in this study.

**3. Preparation of Alkali Metal Salts of α–Oximino Acids**

The alkali salts were prepared according to the literature methods or slight modifications of them.^2^

**Mono[(hydroxyimino)propanoato] Potassium(I) (KHIP), K(HONCCH_3_COO), MW = 141.17**

To a 50 ml stirred de-ionized water, 2-(hydroxyimino)propanoic acid (2 g; 19.4 mM) was dissolved. Potassium carbonate (1.34 g; 9.7 mM) was added to this solution leading to rapid gas evolution. Stirring was continued for 1 hr. The solution was spread inside a petridish to evaporate the solvent giving large clear crystals of the compound. Yield = 2.44 g, 89%.

**Mono[2-(methoxyimino) propanoato] Potassium(I) (KMIP), K(CH_3_ONCCH_3_COO), MW = 155.19**

To 100 ml acetone, 2-(methoxyimino)propanoic acid (1.89 g; 16.1 mM) was added whilst stirring. A clear solution was obtained after dissolution. To this, potassium carbonate (1.1 g; 8.0 mM) was added to the solution. Stirring was continued for 24 hrs. A copious white precipitate was formed which was filtered using a Buchner funnel, washed with acetone, and dried in vacuum. Yield = 2.19 g, 87.6%.

**Mono[2-(ethylhydroxyimino) propanoato] Potassium(I) (KEIP), K(C_2_H_5_ONCCH_3_COO), MW = 169.22**

To 100 ml acetone, 2-(ethylhydroxyimino)propanoic acid (1.74 g; 13.2 mM) was added whilst stirring. A clear solution was obtained after dissolution. To this, potassium carbonate (0.916 g; 6.6.0 mM) was added to the solution. Stirring was continued for 24 hrs. A copious white precipitate was formed which was filtered using a Buchner funnel, washed with acetone, and dried in vacuum. Yield = 2.24 g, 99%.

**FTIR Spectra of the Potassium Oximate Salts**

Figure S2 shows the FTIR spectra of as prepared KHIP, KMIP, and KEIP. All the potassium oximate salts show the presence of characteristics absorption bands associated with (C=N), (N–O), and ν(COO) that can be assigned unambiguously. These bands are tabulated in Table S2.


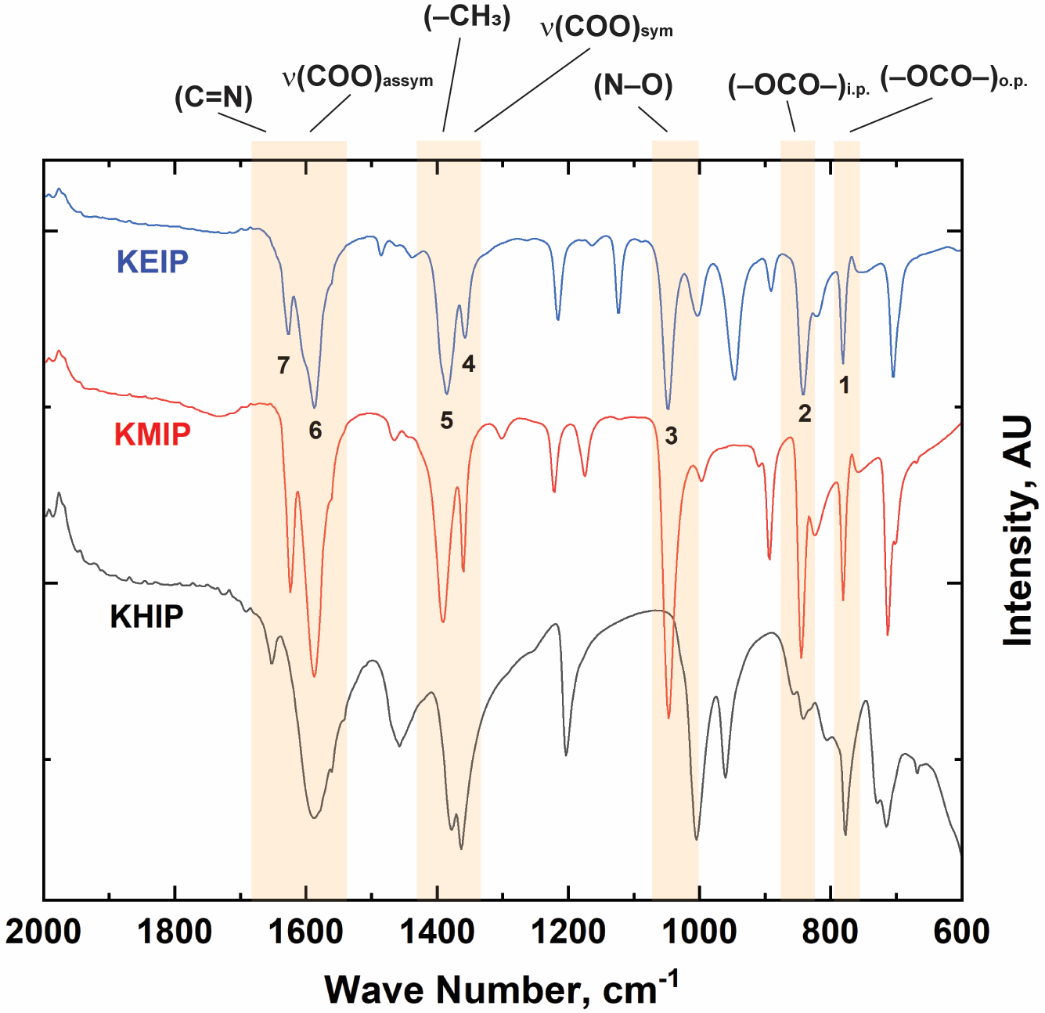


**Figure S2**: FTIR spectra of various potassium oximate salts prepared in this study showing the characteristic absorption peaks associated with the oximate backbone (**R_2_**–C(=NO**R_1_**)COO). Assignment of peaks is shown in Table S2.

| **Number** | **Band, cm^-1^** | **KHIP** | **KMIP** | **KEIP** |
| --- | --- | --- | --- | --- |
| 1 | (–OCO–)_o.p._ | 777 | 781 | 781 |
| 2 | (–OCO–)_i.p._ | 842 | 844 | 842 |
| 3 | (N–O) | 1005 | 1047 | 1048 |
| 4 | ν(COO)_sym_ | 1362 | 1360 | 1358 |
| 5 | (–CH_3_) | – | 1391 | 1384 |
| 6 | ν(COO)_assym_ | 1587 | 1587 | 1588 |
| 7 | (C=N) | 1652 | 1624 | 1627 |

**Table S2**: Characteristic absorption peaks of potassium oximates prepared in this study.

**4. Preparation of Zinc Salts of α–Oximino Acids**

The zinc salts below were prepared according to the literature methods or slight modifications of them.^3-5^

**Diaqua Bis[2-(hydroxyimino)acetato] Zinc(II) (ZnHIA_2_), Zn(HONCHCOO)_2_ . 2H_2_O, MW = 277.47**

Glyoxylic acid (2.76 g, 30 mM) was dissolved in 20 ml deionized water. To this solution, sodium bicarbonate (2.52 g, 30 mM) was added portionwise with vigorous stirring. When the gas evolution subsided, hydroxylamine hydrochloride (2.08 gm, 30 mM) was added. To this mixture, sodium bicarbonate (2.52 g, 30 mM) was added portionwise. The reaction was allowed to continue until no visible gas evolution was seen. To the stirred solution, zinc(II) nitrate hexahydrate (4.46 g, 15 mM) was added. A white precipitate soon appeared. The stirring was continued for 18 hrs. Thereafter it was filtered, washed with ice cold water, and dried overnight in a vacuum oven. Yield = 4.1 g, 98%.

**Diaqua Bis[2-(hydroxyimino)propanoato] Zinc(II) (ZnHIP_2_), Zn(HONCCH_3_COO)_2_ . 2H_2_O, MW = 305.53**

Sodium pyruvate (3.30 g, 30 mM) was dissolved in 24 ml deionized water. To this solution, hydroxylamine hydrochloride (2.08 g, 30 mM) was added with vigorous stirring. The solution turned turbid. To this mixture, sodium bicarbonate (2.52 g, 30 mM) was added portionwise. The turbidity slowly disappeared with the continuing addition of sodium bicarbonate and ultimately the solution became clear. The reaction was allowed to continue until no visible gas evolution was seen. To the stirred solution, zinc(II) nitrate hexahydrate (4.46 g, 15 mM) was added. A white precipitate soon appeared. The stirring was continued for 18 hrs. Thereafter it was filtered, washed with ice cold water, and dried overnight in a vacuum oven. Yield = 4.5 g, 98%.

**Diaqua Bis[2-(methoxyimino)acetato] Zinc(II) (ZnMIA_2_), Zn(CH_3_ONCHCOO)_2_. 2H_2_O, MW = 305.53**

Glyoxylic acid (2.76 g, 30 mM) was dissolved in 20 ml deionized water. To this solution, sodium bicarbonate (2.52 g, 30 mM) was added portionwise with vigorous stirring. When the gas evolution subsided, methoxylamine hydrochloride (2.50 g, 30 mM) was added. To this mixture, sodium bicarbonate (2.52 g, 30 mM) was added portionwise. The reaction was allowed to continue until no visible gas evolution was seen. To the stirred solution, zinc(II) nitrate hexahydrate (4.46 g, 15 mM) was added. A white precipitate soon appeared. The stirring was continued for 18 hrs. Thereafter it was filtered, washed with ice cold water, and dried overnight in a vacuum oven. Yield = 1.55 g, 33.8%.

**Diaqua Bis[2-(methoxyimino)propanoato] Zinc(II) (ZnMIP_2_), Zn(CH_3_ONCCH_3_COO)_2_ . 2H_2_O, MW = 333.61**

Sodium pyruvate (3.30 g, 30 mM) was dissolved in 20 ml deionized water. To this solution, methoxylamine hydrochloride (2.50 g, 30 mM) was added with vigorous stirring. The solution turned turbid. To this mixture, sodium bicarbonate (2.52 g, 30 mM) was added portionwise. The turbidity slowly disappeared with the continuing addition of sodium bicarbonate and ultimately the solution became clear. The reaction was allowed to continue until no visible gas evolution was seen. To the stirred solution, zinc(II) nitrate hexahydrate (4.46 g, 15 mM) was added. A white precipitate soon appeared. The stirring was continued for 18 hrs. Thereafter it was filtered, washed with ice cold water, and dried overnight in a vacuum oven. Yield = 2.4 g, 48%.

**Diaqua Bis[2-(ethyl hydroxyimino)propanoato] Zinc(II) (ZnEIP_2_), Zn(C_2_H_5_ONCCH_3_COO)_2_ . 2H_2_O, MW = 361.63**

Ethyl hydroxylamine hydrochloride (2.92 g, 30 mM) was dissolved in 35 ml deionized water. To this vigorously stirred solution, pyruvic acid (2.08 ml, 30 mM) was added. The solution became turbid. To this mixture, sodium bicarbonate (5.04 g, 60 mM) was added portionwise. The turbidity slowly disappeared with the continuing addition of sodium bicarbonate and ultimately the solution became clear. The reaction was allowed to continue until no visible gas evolution was seen. To the stirred solution, zinc(II) nitrate hexahydrate (4.46 g, 15 mM) was added portionwise. A white precipitate immediately. The stirring was continued for 2 hrs. Thereafter it was filtered, washed with ice cold water, and dried overnight in a vacuum oven. Yield = 3.8 g, 70%.

**FTIR Spectra of the Zinc Oximates**

Figure S3 shows the FTIR spectra of as prepared ZnHIA_2_, ZnHIP_2_, ZnMIA_2_, ZnMIP_2_, and ZnEIP_2_. All the zinc oximates show the presence of characteristics absorption bands associated with (C=N), (N–O), and ν(COO) that can be assigned unambiguously. These bands are tabulated in Table S3.


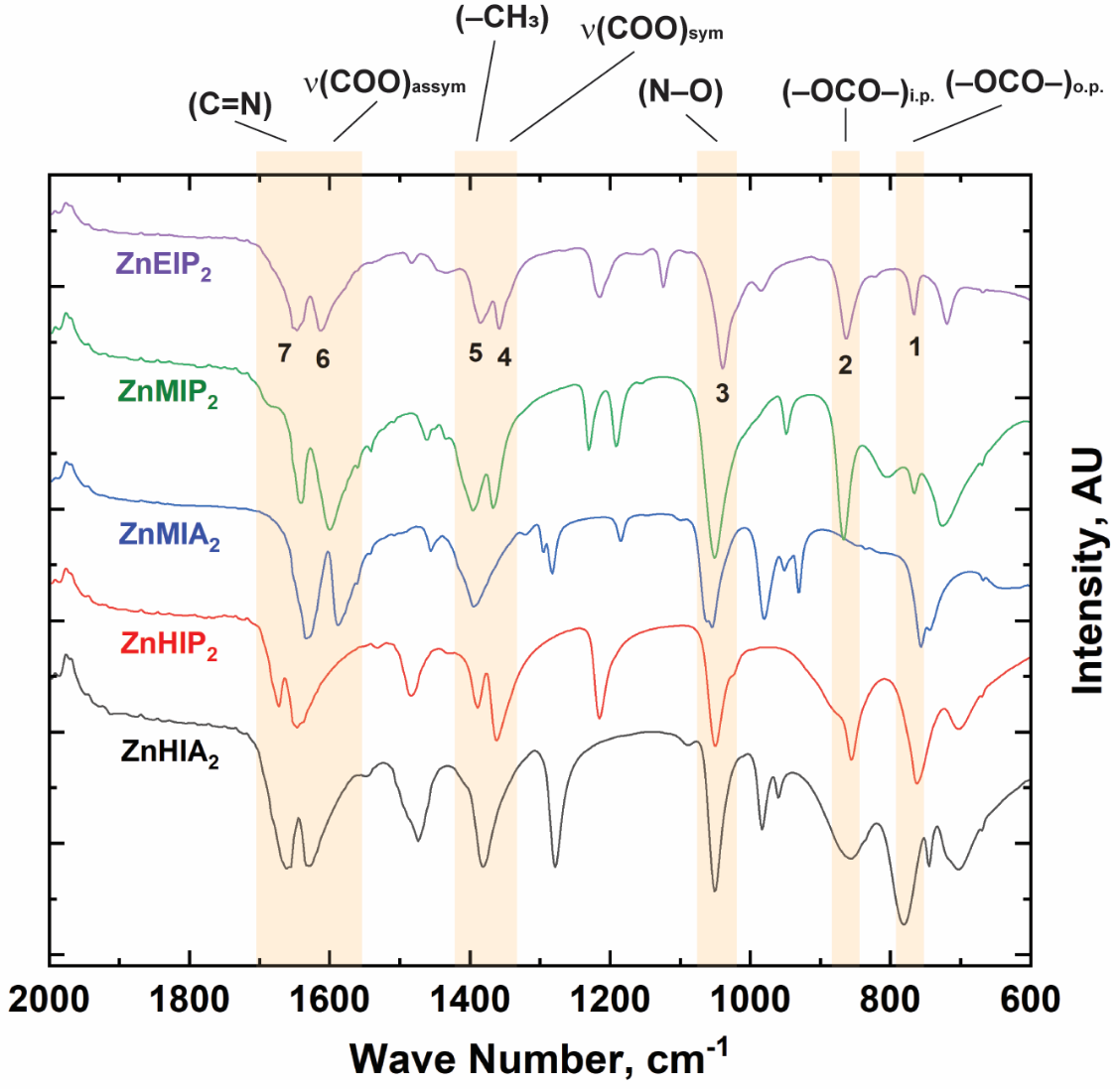


**Figure S3**: FTIR spectra of various zinc resists prepared in this study showing the characteristic absorption peaks associated with the oximate backbone (**R_2_**–C(=NO**R_1_**)COO). Assignment of peaks is shown in Table S3.

| **Number** | **Band, cm^-1^** | **ZnHIA_2_** | **ZnHIP_2_** | **ZnMIA_2_** | **ZnMIP_2_** | **ZnEIP_2_** |
| --- | --- | --- | --- | --- | --- | --- |
| 1 | (–OCO–)_o.p._ | 780 | 762 | 756 | 766 | 767 |
| 2 | (–OCO–)_i.p._ | 856 | 856 | – | 866 | 863 |
| 3 | (N–O) | 1050 | 1049 | 1054 | 1050 | 1039 |
| 4 | ν(COO)_sym_ | 1381 | 1361 | 1394 | 1366 | 1358 |
| 5 | (–CH_3_) | – | 1389 | – | 1395 | 1384 |
| 6 | ν(COO)_assym_ | 1630 | 1646 | 1588 | 1599 | 1613 |
| 7 | (C=N) | 1661 | 1672 | 1633 | 1641 | 1647 |

**Table S3**: Characteristic absorption peaks of various zinc resists prepared in this study.

**5. Preparation of Metals Salts of α–Oximino Acids**

The metal salts below were prepared according to the literature methods or slight modifications of them.^5-10^

**Bis[2-(methoxyimino)propanoato] Tin(II) (SnMIP_2_), Sn(CH_3_ONCCH_3_COO)_2_, MW = 350.9**

Potassium methoxyimino propanoate (1.24 g, 8 mM) was suspended in methanol (50 ml). To this, anhydrous tin(II) chloride (0.76 g, 4 mM) was added and stirred for 18 hours. The turbid solution was filtered and the solvent was evaporated. To the dried powder, dichloromethane (20 ml) was added. The precipitate that appeared was filter. The filtrate was added dropwise to 200 ml of n-hexane. It turned turbid with a white product coating the walls. After removal of n-hexane, the product was dried in a vacuum oven for 17 hrs. The product is an off-white solid. Yield: 0.44 g, 31%.

**Tris[2-(methoxyimino)propanoato] Aluminium(III) (AlMIP_3_), Al(CH_3_ONCCH_3_COO)_3_, MW = 375. 27**

Potassium methoxyimino propanoate (2.27 g, 14.6 mM) was suspended in methanol (60 ml). To this, aluminium nitrate nonahydrate (1.83 g, 4.87 mM) was added and stirred for 2 hours. The turbid solution was filtered and the solvent was evaporated. To the dried powder, dichloromethane (20 ml) was added. A precipitate appeared that was filtered. The filtrate was added dropwise to 300 ml of hexane. It turned turbid with a white product coating the walls of the beaker. After removal of hexane, the product was dried in a vacuum oven for 17 hrs. The product is a white solid. Yield: 1.06 g, 56%.

**Tris[2-(hydroxyimino)propanoato] Indium(III) (InHIP_3_), In(HONCCH_3_COO)_3_, MW = 421.02**

Hydroxylamine hydrochloride (2.08 g, 30 mM) was dissolved in 20 ml of deionized water. To this sodium bicarbonate (2.52 g, 30 mM) was added portionwise. When the gas evolution subsided, sodium pyruvate (3.30 g, 30 mM) was added. The reaction was allowed to continue until no visible gas evolution was seen. To the stirred solution, indium(III) nitrate (3.0 g, 10 mM) was added. A white precipitate soon appeared. The stirring was continued for 3 hrs. Thereafter it was filtered, washed with ice cold water and dried overnight in a vacuum oven. Yield = 3.41 g, 81%.

**Tris[2-(methoxyimino)propanoato] Indium(III) (InMIP_3_), In(CH_3_ONCCH_3_COO)_3_, MW = 463. 10**

Potassium methoxyimino propanoate (2.27 g, 14.6 mM) was suspended in methanol (50 ml). To this, anhydrous indium(III) chloride (1.08 g, 4.88 mM) was added and stirred for 2 hours. The turbid solution was filtered and the solvent was evaporated. To the dried powder, dichloromethane (20 ml) was added. A precipitate that appeared was filtered. The filtrate was added dropwise to 300 ml of n-hexane. It turned turbid with a transparent to white product coating the walls of the beaker. After removal of hexane, the product was dried in a vacuum oven for 20 hrs. The product is a white solid. Yield: 1.73 g, 76%.

**Bis[2-(methoxyimino)propanoato] Nickel(II) (NiMIP_2_), Ni(CH_3_ONCCH_3_COO)_2_, MW = 290.88**

Sodium pyruvate (3.30 g, 30 mM) was dissolved in 20 ml deionized water. To this solution, methoxylamine hydrochloride (2.50 g, 30 mM) was added with vigorous stirring. The solution turned turbid. To this mixture, sodium bicarbonate (2.52 gm, 30 mM) was added portionwise. The turbidity slowly disappeared with the continuing addition of sodium bicarbonate and ultimately the solution became clear. The reaction was allowed to continue until no visible gas evolution was seen. To the stirred solution, nickel(II) acetate tetrahydrate (3.73 g, 15 mM) was added. A light blue precipitate soon appeared. The stirring was continued for 3 hrs. Thereafter it was filtered, washed with ice cold water, and dried overnight in a vacuum oven. Yield = 3.5 g, 78%.

**Trisaqua Bis[2-(methoxyimino)propanoato] Magnesium(II) (MgMIP_2_), Mg(CH_3_ONCCH_3_COO)_2_ . 3H_2_O, MW = 310.50**

Sodium pyruvate (3.30 g, 30 mM) was dissolved in 20 ml deionized water. To this solution, methoxylamine hydrochloride (2.50 g, 30 mM) was added with vigorous stirring. The solution turned turbid. To this mixture, sodium bicarbonate (2.52 g, 30 mM) was added portionwise. The turbidity slowly disappeared with the continuing addition of sodium bicarbonate and ultimately the solution became clear. The reaction was allowed to continue until no visible gas evolution was seen. To the stirred solution, magnesium(II) sulfate (1.80 g, 15 mM) was added. The solution remained clear. The stirring was continued for 3 hrs. Thereafter the solution was allowed to slowly evaporate inside the fumehood. After 3 days, crystals of the product were obtained in the bottom of the solution. The mother liquor was discarded, the crystals were washed with ice cold water, and dried overnight in a vacuum oven. Yield = 1.68 g, 36%.

**FTIR Spectra of the Metal Oximates**

Figure S4 shows the FTIR spectra of as prepared SnMIP_2_, InMIP_3_, InHIP_3_, NiMIP_2_, ZnMIP_2_, MgMIP_2_, and AlMIP_3_. All the metal oximates show the presence of characteristics absorption bands associated with (C=N), (N–O), and ν(COO) that can be assigned unambiguously. These bands are tabulated in Table S4.


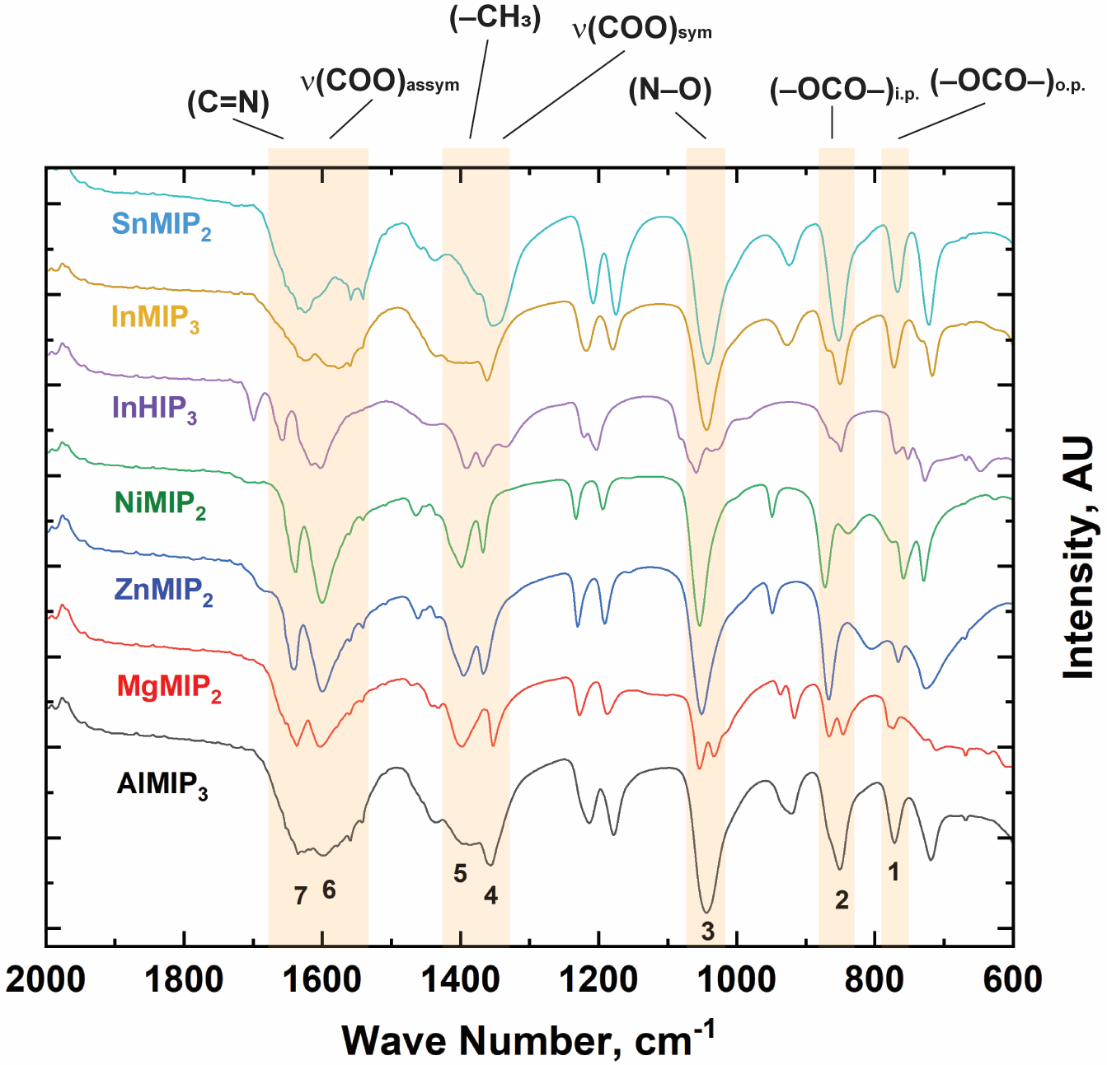


**Figure S4**: FTIR spectra of various metal-oximate resists prepared in this study showing the characteristic absorption peaks associated with the oximate backbone (**R_2_**–C(=NO**R_1_**)COO). Assignment of peaks is shown in Table S4.

| **Number** | **Band, cm^-1^** | **AlMIP_3_** | **MgMIP_2_** | **ZnMIP_2_** | **NiMIP_2_** | **InHIP_3_** | **InMIP_3_** | **SnMIP_2_** |
| --- | --- | --- | --- | --- | --- | --- | --- | --- |
| 1 | (–OCO–)_o.p._ | 772 | 773 | 766 | 758 | 752 | 772 | 767 |
| 2 | (–OCO–)_i.p._ | 851 | 866 | 866 | 872 | 849 | 850 | 852 |
| 3 | (N–O) | 1043 | 1053 | 1050 | 1054 | 1059 | 1043 | 1041 |
| 4 | ν(COO)_sym_ | 1356 | 1352 | 1366 | 1367 | 1367 | 1362 | 1353 |
| 5 | (–CH_3_) | 1392 | 1397 | 1395 | 1398 | 1391 | – | 1374 |
| 6 | ν(COO)_assym_ | 1599 | 1602 | 1599 | 1600 | 1601 | 1576 | 1558 |
| 7 | (C=N) | 1635 | 1637 | 1641 | 1634 | 1657 | 1624 | 1625 |

**Table S4**: Characteristic absorption peaks of various metal oximate resists prepared in this study.

**6. EBL and EUVL Contrast Curves of Different Resists and Their Relationship**

**A) Zinc Resist (ZnMIP_2_)**


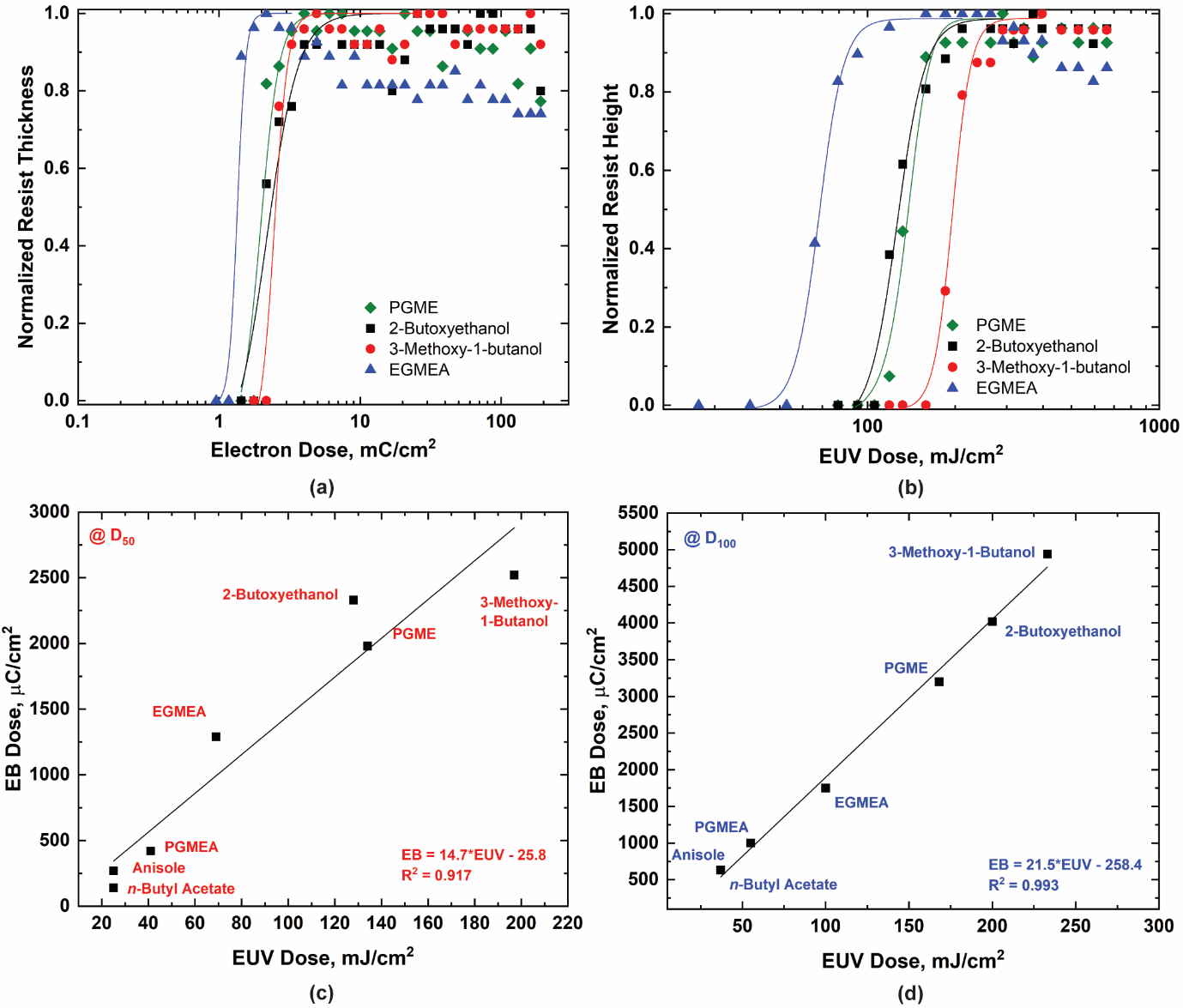


**Figure S5**: Contrast curves of ZnMIP_2_ resist derived from (a) EBL, and (b) EUVL for different developers. (c) For ZnMIP_2_ resist, relationship between EB and EUV doses at 50% normalized thickness, and (d) at 100% normalized resist thickness for different developers. Notice the correlation at 100% normalized resist thickness is far better than at 50% normalized thickness. Check Figure 2 and Table I in the manuscript for other developers.

| **Developer** | **EUVL, mJ/cm^2^** | | **EBL, mC/cm^2^** | |
| --- | --- | --- | --- | --- |
|  | **Sensitivity, D_50_** | **D_100_** | **Sensitivity, D_50_** | **D_100_** |
| 2-Butoxyethanol | 128 | 200 | 2.33 | 4.02 |
| 3-Methoxy-1-butanol | 197 | 233 | 2.52 | 4.94 |

**Table S5**: Sensitivity values for different developers obtained from (a) EBL and (b) EUVL of ZnMIP_2_ resist.

**B) Nickel Resist (NiMIP_2_)**


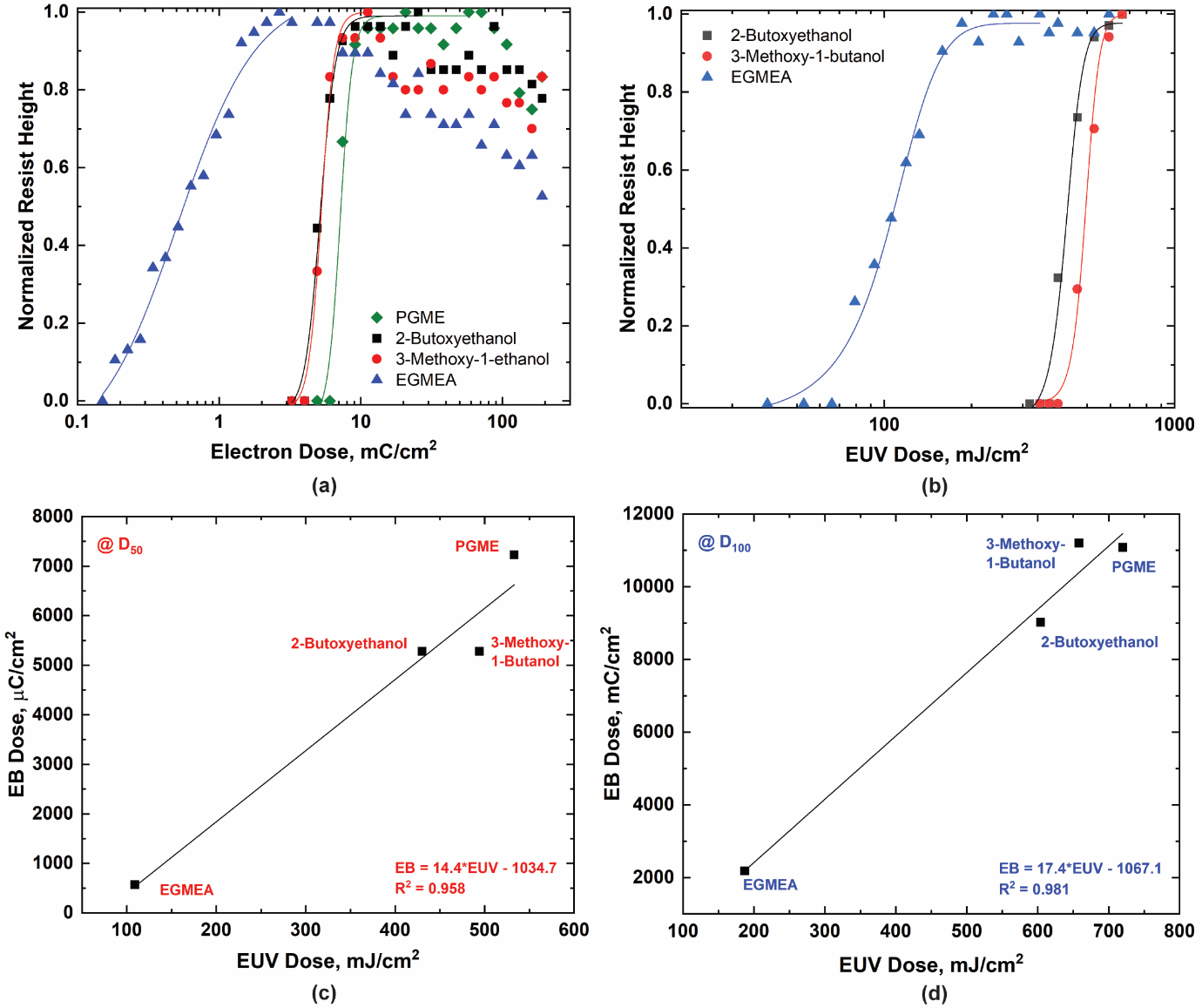


**Figure S6**: Contrast curves of NiMIP_2_ resist derived from (a) EBL, and (b) EUVL for different developers. (c) For NiMIP_2_ resist, relationship between EB and EUV doses at 50% normalized thickness, and (d) at 100% normalized resist thickness for different developers. Notice the correlation at 100% normalized resist thickness is far better than at 50% normalized thickness.

| **Developer** | **EUVL, mJ/cm^2^** | | **EBL, mC/cm^2^** | |
| --- | --- | --- | --- | --- |
|  | **Sensitivity, D_50_** | **D_100_** | **Sensitivity, D_50_** | **D_100_** |
| PGME | 533 | 720 | 7.23 | 11.08 |
| 2-Butoxyethanol | 430 | 604 | 5.28 | 9.02 |
| 3-Methoxy-1-butanol | 494 | 658 | 5.28 | 11.2 |
| EGMEA | 108 | 187 | 0.57 | 2.186 |

**Table S6**: Sensitivity values for different developers obtained from (a) EBL and (b) EUVL of NiMIP_2_ resist.

**C) Magnesium Resist (MgMIP_2_)**


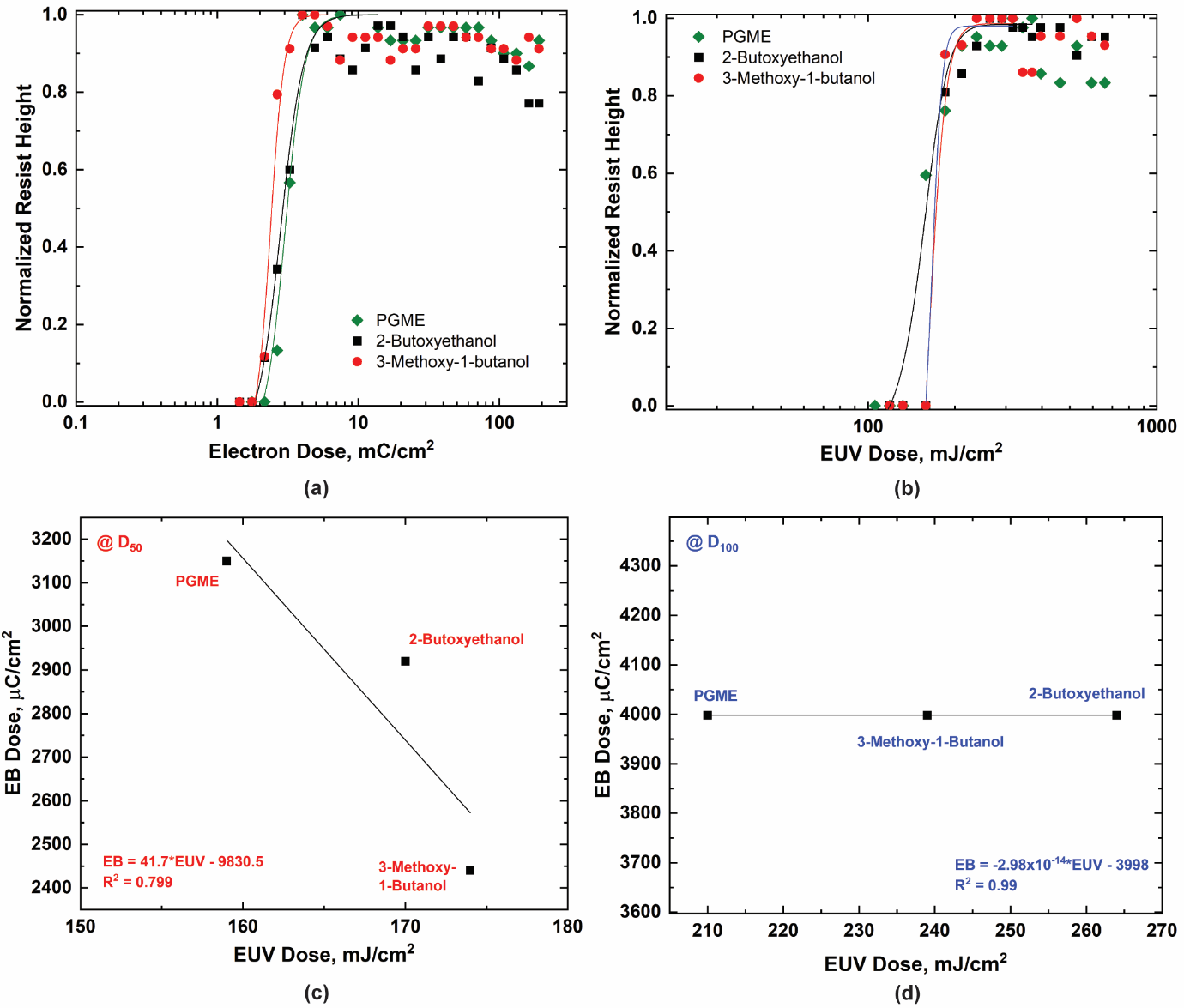


**Figure S7**: Contrast curves of MgMIP_2_ resist derived from (a) EBL, and (b) EUVL for different developers. (c) For MgMIP_2_ resist, relationship between EB and EUV doses at 50% normalized thickness, and (d) at 100% normalized resist thickness for different developers. Notice the correlation at 100% normalized resist thickness is far better than at 50% normalized thickness.

| **Developer** | **EUVL, mJ/cm^2^** | | **EBL, mC/cm^2^** | |
| --- | --- | --- | --- | --- |
|  | **Sensitivity, D_50_** | **D_100_** | **Sensitivity, D_50_** | **D_100_** |
| PGME | 159 | 210 | 3.15 | 3.99 |
| 2-Butoxyethanol | 170 | 264 | 2.92 | 4.01 |
| 3-Methoxy-1-butanol | 174 | 239 | 2.44 | 4.01 |

**Table S7**: Sensitivity values for different developers obtained from (a) EBL and (b) EUVL of MgMIP_2_ resist.

**D) Indium (A) Resist (InHIP_3_)**


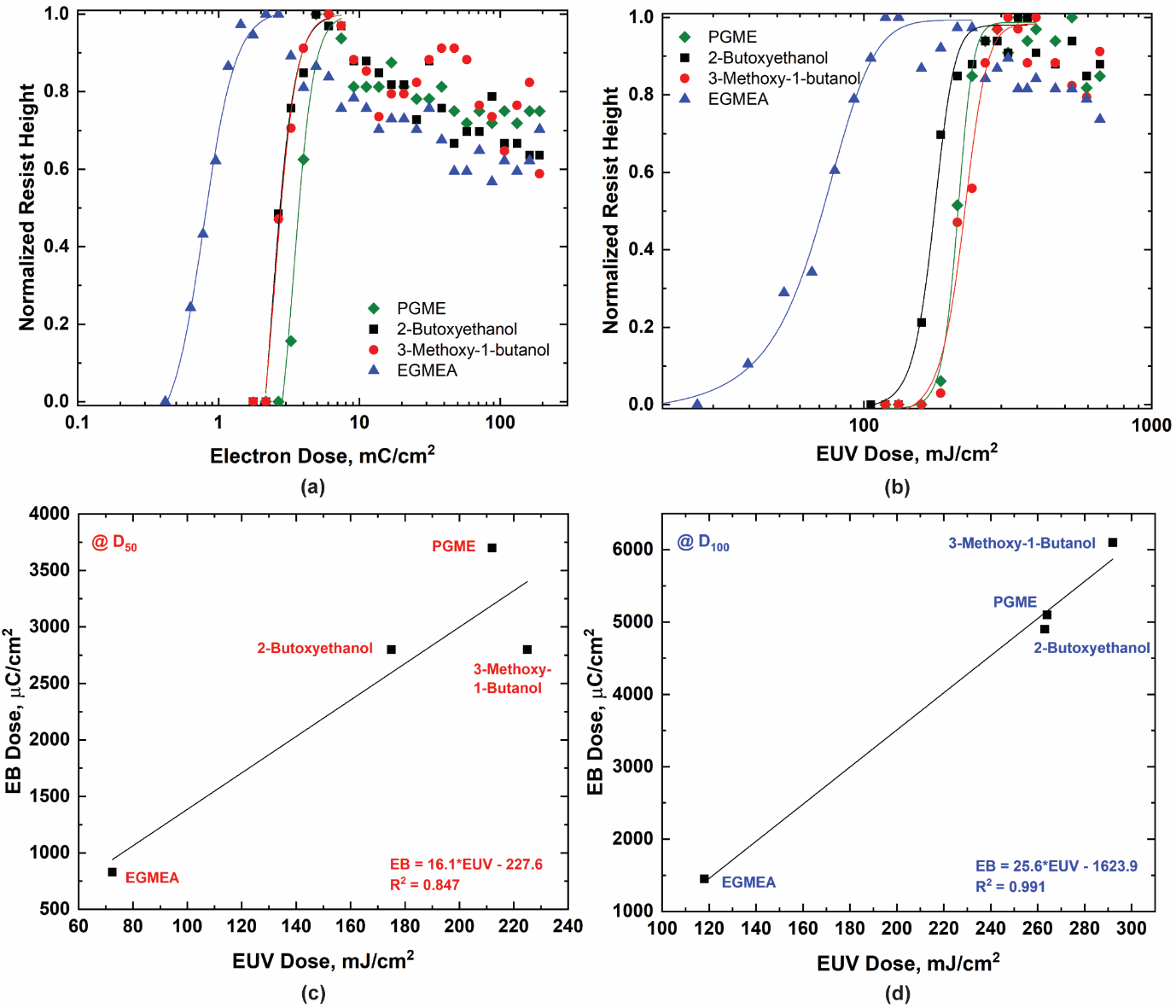


**Figure S8**: Contrast curves of InHIP_3_ resist derived from (a) EBL, and (b) EUVL for different developers. (c) For InHIP_3_ resist, relationship between EB and EUV doses at 50% normalized thickness, and (d) at 100% normalized resist thickness for different developers. Notice the correlation at 100% normalized resist thickness is far better than at 50% normalized thickness.

| **Developer** | **EUVL, mJ/cm^2^** | | **EBL, mC/cm^2^** | |
| --- | --- | --- | --- | --- |
|  | **Sensitivity, D_50_** | **D_100_** | **Sensitivity, D_50_** | **D_100_** |
| PGME | 212 | 264 | 3.7 | 5.1 |
| 2-Butoxyethanol | 175 | 263 | 2.8 | 4.9 |
| 3-Methoxy-1-butanol | 225 | 292 | 2.8 | 6.1 |
| EGMEA | 72 | 118 | 0.83 | 1.45 |

**Table S8**: Sensitivity values for different developers obtained from (a) EBL and (b) EUVL of InHIP_3_ resist.

**E) Indium (B) Resist (InMIP_3_)**


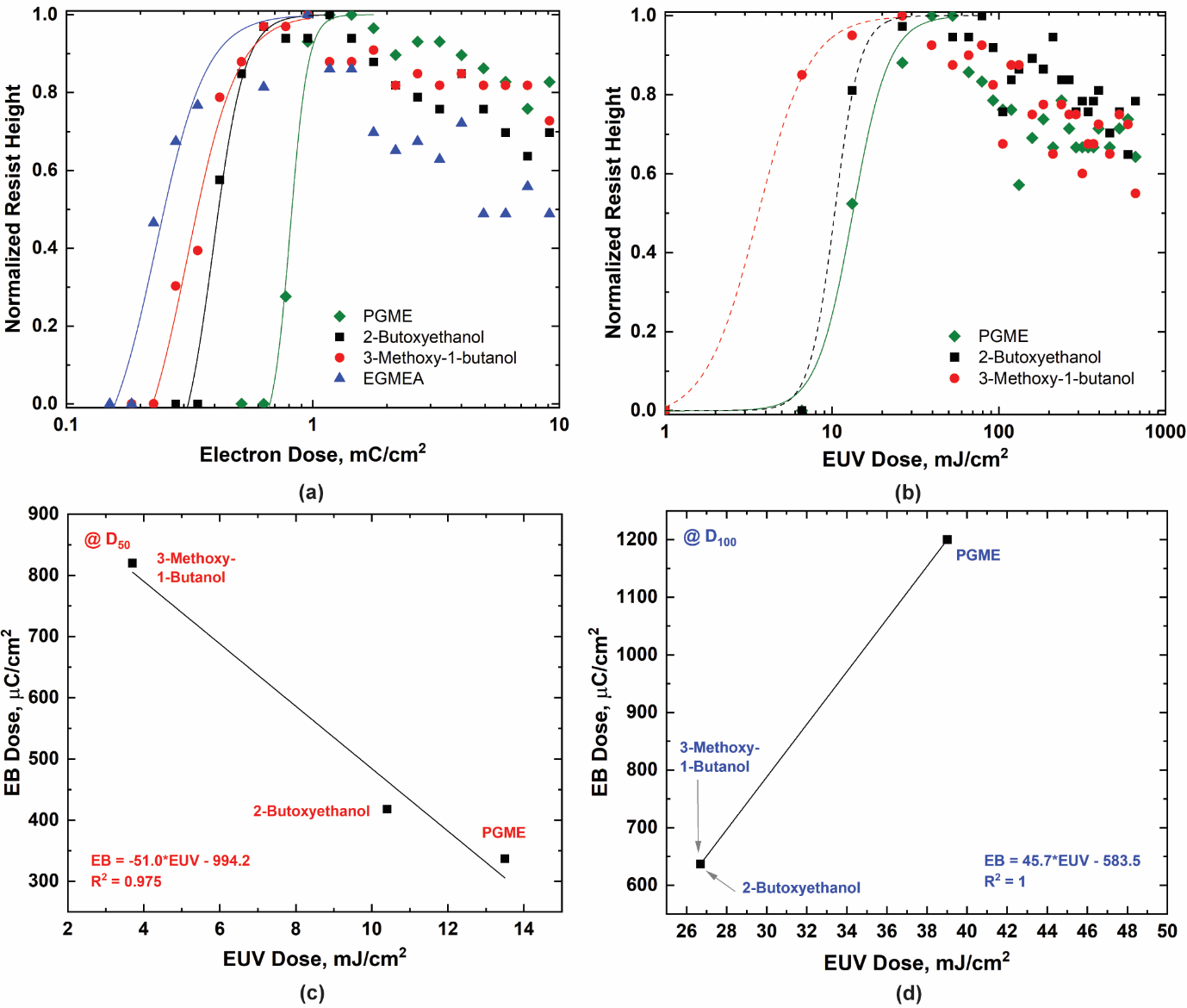


**Figure S9**: Contrast curves of InMIP_3_ resist derived from (a) EBL, and (b) EUVL for different developers. (c) For InMIP_3_ resist, relationship between EB and EUV doses at 50% normalized thickness, and (d) at 100% normalized resist thickness for different developers. Notice the correlation at 100% normalized resist thickness is far better than at 50% normalized thickness. The dotted line indicates uncertainty in measurement of patterned height due to chemical instability of the resist.

| **Developer** | **EUVL, mJ/cm^2^** | | **EBL, mC/cm^2^** | |
| --- | --- | --- | --- | --- |
|  | **Sensitivity, D_50_** | **D_100_** | **Sensitivity, D_50_** | **D_100_** |
| PGME | 13.5 | 39 | 0.82 | 1.2 |
| 2-Butoxyethanol | 10.4 | 26.7 | 0.418 | 0.637 |
| 3-Methoxy-1-butanol | 3.7 | 26.7 | 0.337 | 0.637 |
| EGMEA | – | – | 0.250 | 0.630 |

**Table S9**: Sensitivity values for different developers obtained from (a) EBL and (b) EUVL of InMIP_3_ resist.

**F) Aluminium Resist (AlMIP_3_)**


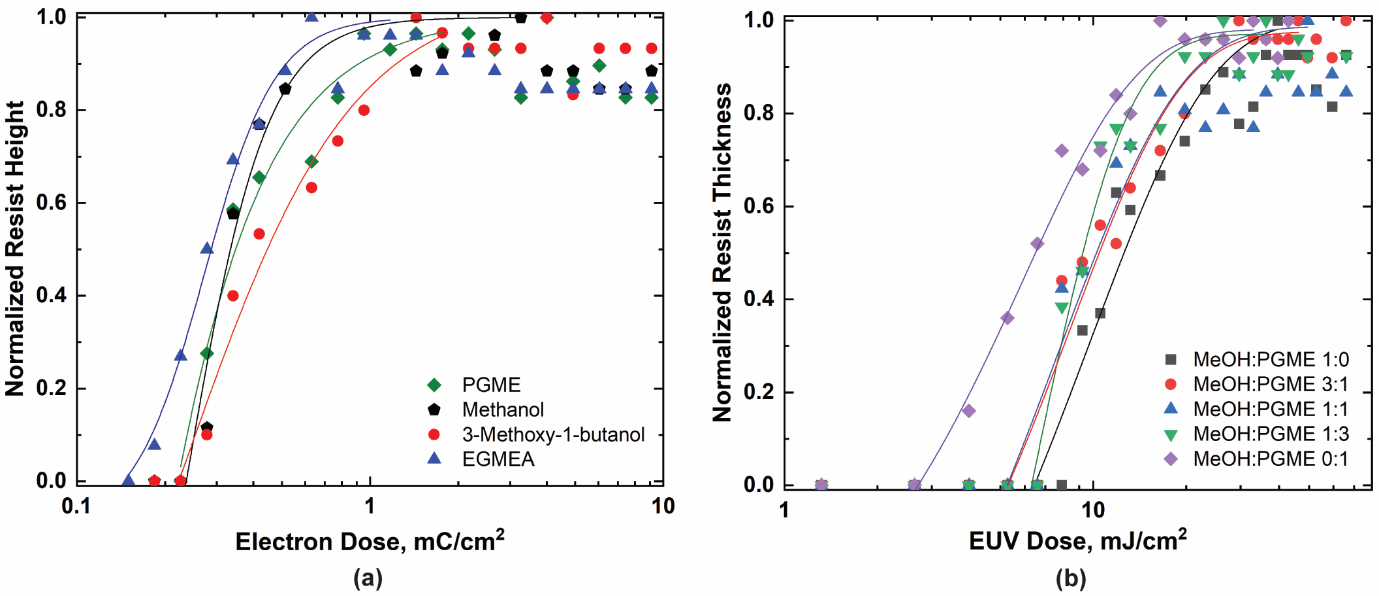


**Figure S10**: Contrast curves of AlMIP_3_ resist derived from (a) EBL, and (b) EUVL for different developers.

| **Developer** | **EUVL, mJ/cm^2^** | | **EBL, mC/cm^2^** | |
| --- | --- | --- | --- | --- |
|  | **Sensitivity, D_50_** | **D_100_** | **Sensitivity, D_50_** | **D_100_** |
| PGME | 6.5 | 16.6 | 0.347 | 0.946 |
| Methanol | 12.8 | 18.0 | 0.334 | 0.632 |
| 3-Methoxy-1-butanol | 8.7 | 38.8 | 0.446 | 1.44 |
| EGMEA | – | – | 0.286 | 0.624 |

**Table S10**: Sensitivity values for different developers obtained from (a) EBL and (b) EUVL of AlMIP_3_ resist.

**G) Tin(II) Resist (SnMIP_2_)**


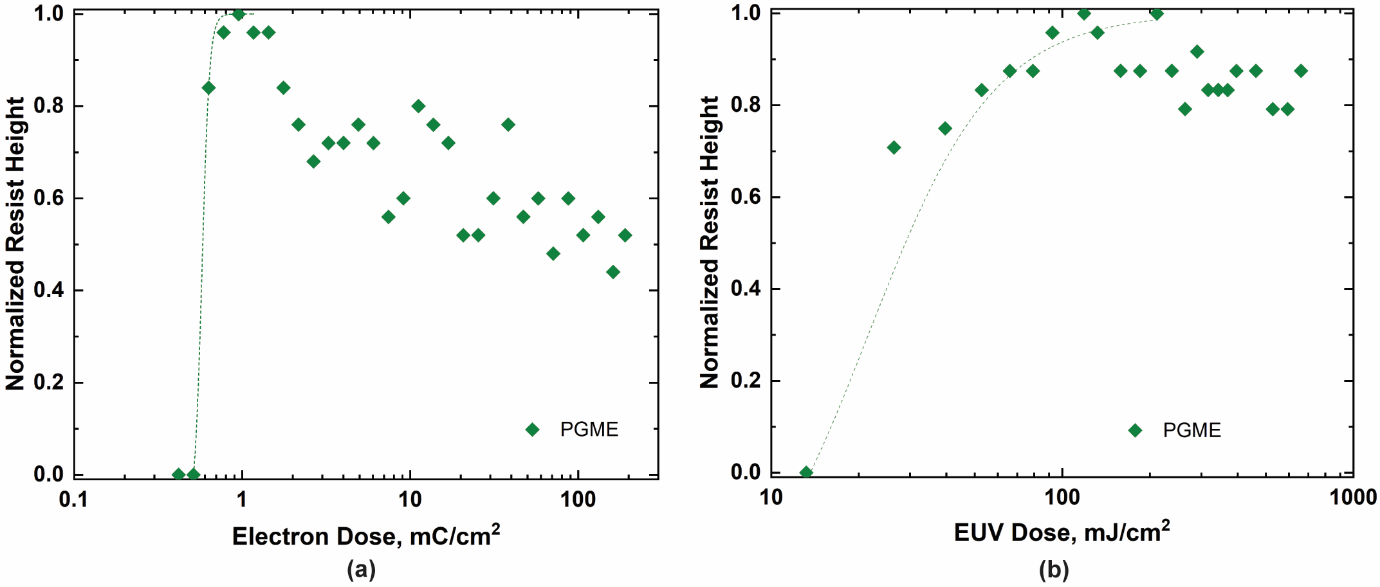


**Figure S11**: Contrast curves of SnMIP_2_ resist derived from (a) EBL, and (b) EUVL.The dotted line indicates uncertainty in measurement of patterned height due to chemical instability of the resist.

| **Developer** | **EUVL, mJ/cm^2^** | | **EBL, mC/cm^2^** | |
| --- | --- | --- | --- | --- |
|  | **Sensitivity, D_50_** | **D_100_** | **Sensitivity, D_50_** | **D_100_** |
| PGME | – | 93 | – | 0.793 |

**Table S11**: Sensitivity values for different developers obtained from (a) EBL and (b) EUVL of SnMIP_2_ resist.

**7. Relationship Between a Developer & Different Metal-Containing Resists in EBL and EUVL**

In Figure 2(d) we noticed that there is a linear correlation between electron beam and EUV doses for different metal-containing resists at 100% normalized thickness when PGME is used as a developer. Does it also hold true for other developers? Figure S12 shows it also holds true for 2-butoxyethanol and 3-methoxy-1-butanol developers. Due to the poor solubility of NiMIP_2_, MgMIP_2_, and InHIP_2_ resists in solvents such as PGMEA, anisole, and *n*-butyl acetate (they can’t be employed as developers), such curves could not be drawn for these developers.


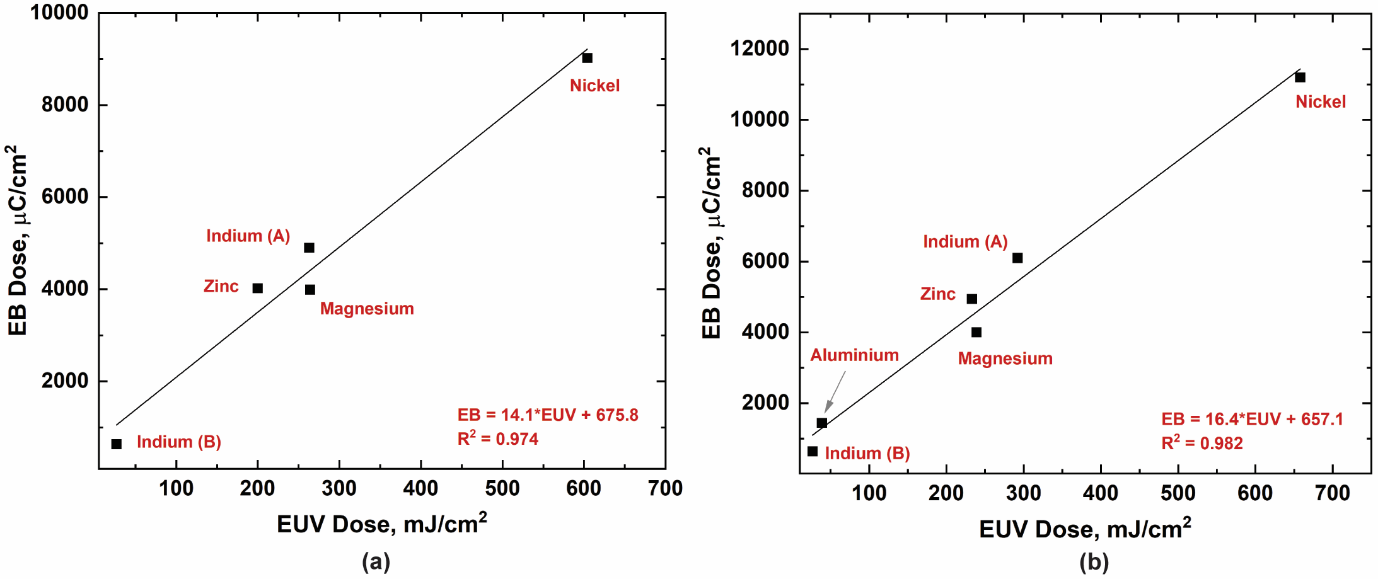


**Figure S12**: Relationship between electron beam and EUV doses (at 100% normalized thickness) for different metal-containing resists when (a) 2-butoxyethanol and (b) 3-methoxy-1-butanol are used as developers.

**8. Hansen Solubility Parameters**

The Hansen solubility parameters are an extension of the Hildebrand solubility parameters and is a way of predicting if one material will dissolve in another and form a solution.^11^ Grounded on the “like dissolves like” rule, the interactions between a solute and a solvent can be described using the Hansen Solubility Parameters, δD, δP, and δH. This parameter is characterized by the three intermolecular interactions, namely, nonpolar (dispersive) interaction (δD), polar interactions (δP), and hydrogen bonding interaction (δH).

| **Hansen Solubility Parameters** | | | |
| --- | --- | --- | --- |
| **Solvent** | **Dispersion, δD** | **Polarity, δP** | **Hydrogen Bonding, δH** |
| *n*-Butyl acetate | 15.8 | 3.7 | 6.3 |
| Anisole | 17.8 | 4.1 | 6.7 |
| PGMEA | 15.6 | 5.6 | 9.8 |
| EGMEA | 15.9 | 5.5 | 11.6 |
| PGME | 15.6 | 6.3 | 11.6 |
| EGME | 16.2 | 9.2 | 16.4 |
| 2-Butoxyethanol | 16.0 | 5.1 | 12.3 |
| 3-Methoxy-1-butanol | 15.3 | 5.4 | 13.6 |
| Methanol | 15.1 | 12.3 | 22.3 |

**Table S12**: The Hansen solubility parameters of various solvents used in this study.^12^ Notice that amongst the solvents chosen, δD does not seem to have much effect as compared to δP and δH.


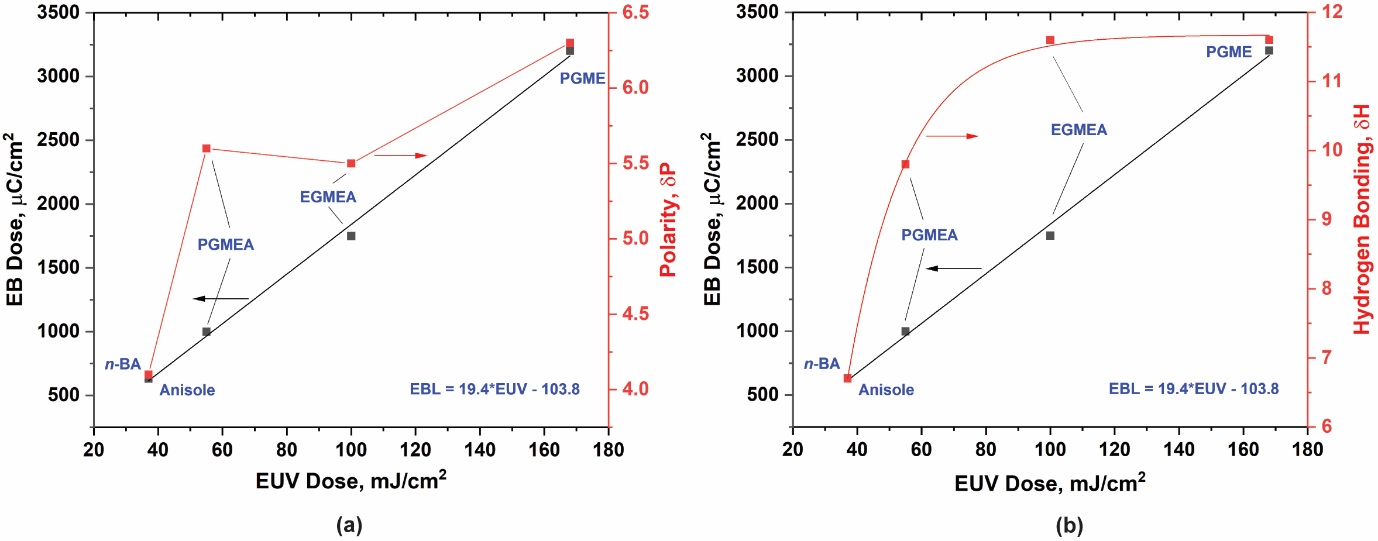


**Figure S13**: In ZnMIP_2_ resist, the dose required to achieve 100% normalized resist thickness for different developers using EBL and EUVL follows the trend in δP as well as δH of the solvents.

The Hansen solubility parameters provide an empirical and semi-quantitative method to choose developers for lithography (Figure S13). In our case, by tweaking the δP and δH of solvents, we were able to modify the sensitivity and contrast of the resists that we tested. This way scumming can also be either minimized or eliminated.

**9. Relationship between EUV Absorption Cross Section and EUV Dose**

Figure S14 shows the relationship between EUV dose needed to reach 100% normalized thickness for different metal-containing resists and the corresponding EUV absorption cross-section of the metal. The absence of relationship suggests that there are other mechanisms responsible for high sensitivity in resists.


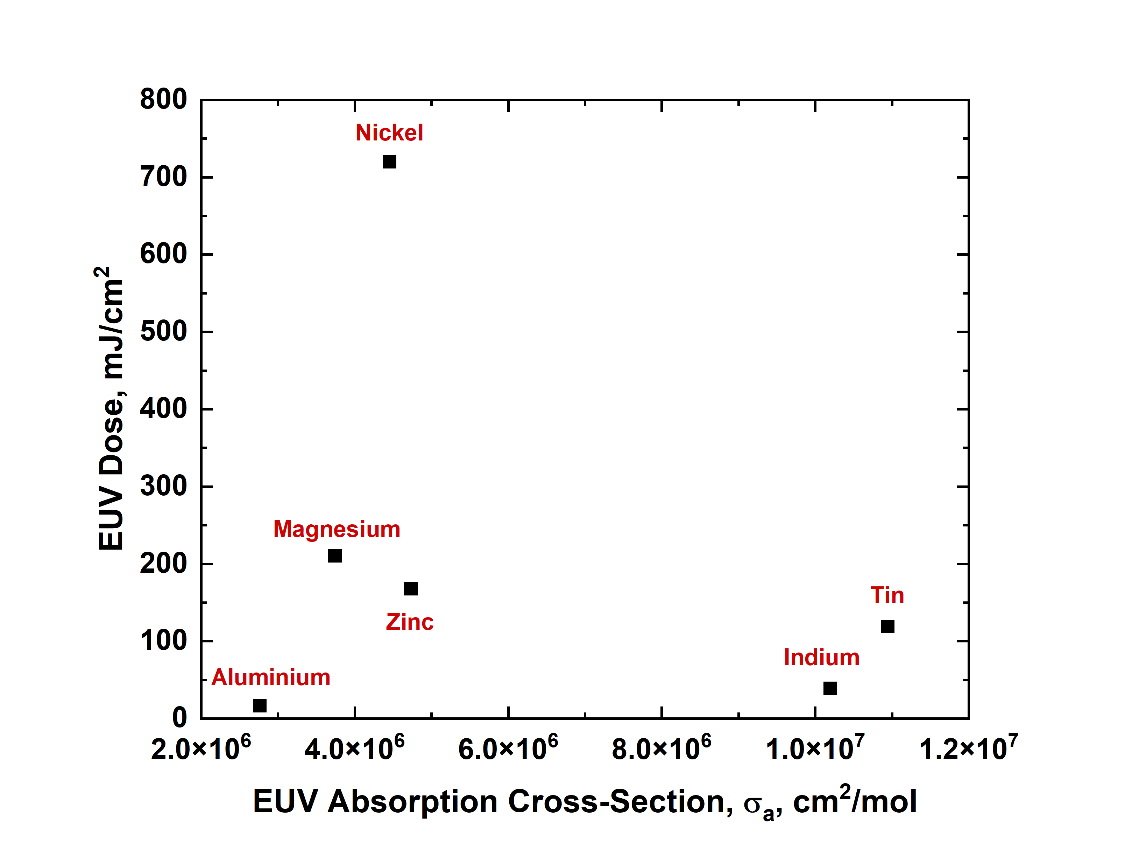


**Figure S14**: Relationship between the EUV dose and EUV absorption cross-section for different metal-containing resists. The dose indicated here is the dose needed to reach 100% normalized thickness (D_100_). The developer in question is PGME.

**10. Patterning Scheme Adopted to Demonstrate Large Area Lithography**


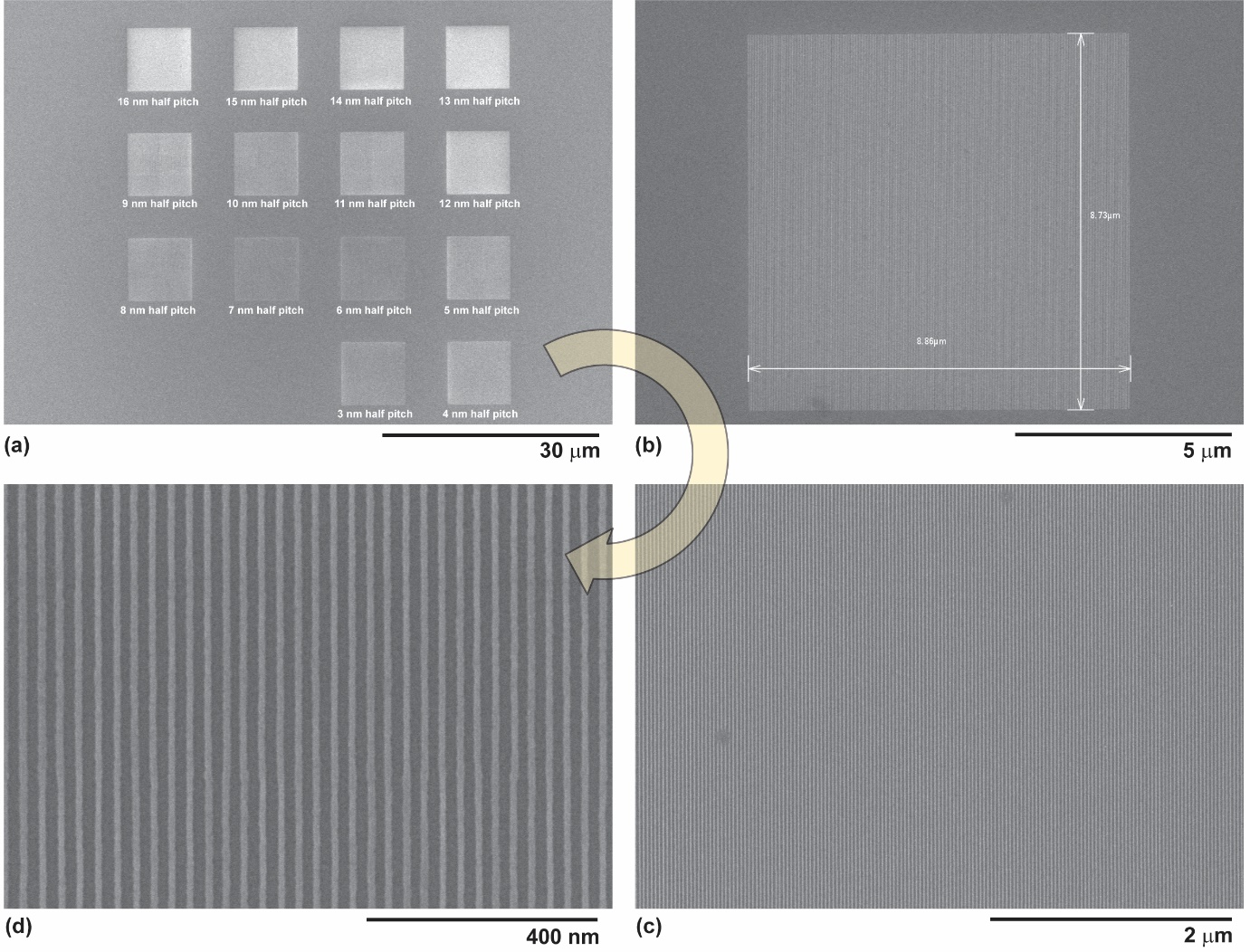


**Figure S15**: (a) SEM image showing the design of 1:1 line/space features from 16 nm half pitch to 3 nm half pitch, at a resolution of 1 nm. (b) Approximately 9 µm $\times$ 9 µm area for each half pitch was patterned. (c) and (d) showing clean patterning of lines over large areas. PGME is the developer.

**11. EBL and Development of ZnMIP_2_ with Anisole and n-Butyl Acetate**


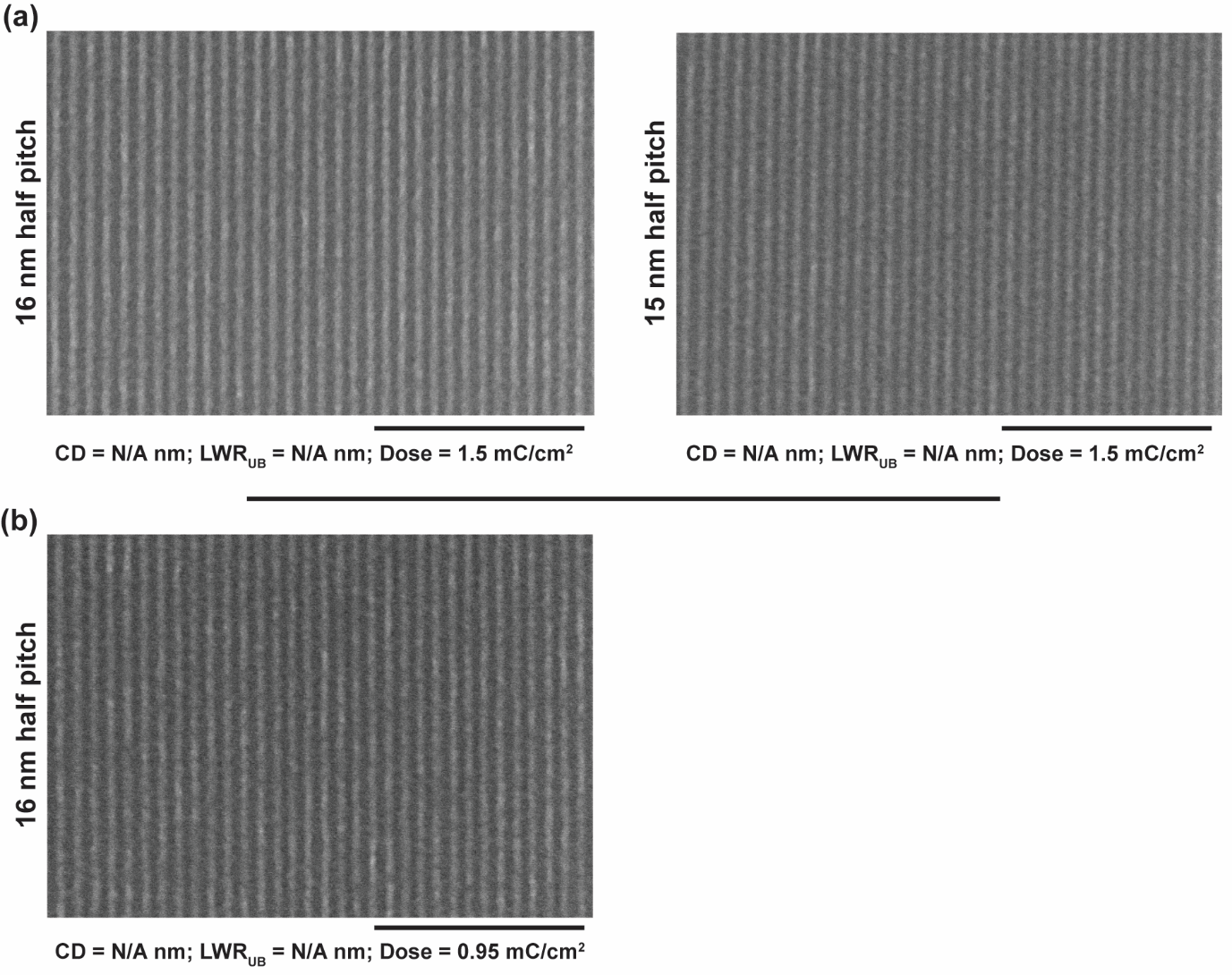


**Figure S16**: SEM images show the development of ZnMIP_2_ resist after EBL using (a) anisole and (b) *n*-butyl acetate as developers. Since both are low contrast developers, it is not surprising to see the presence of scum between the closely spaced lines (16 nm half pitch) even at low doses.

**12. EBL and Development of ZnMIP_2_ with PGMEA**


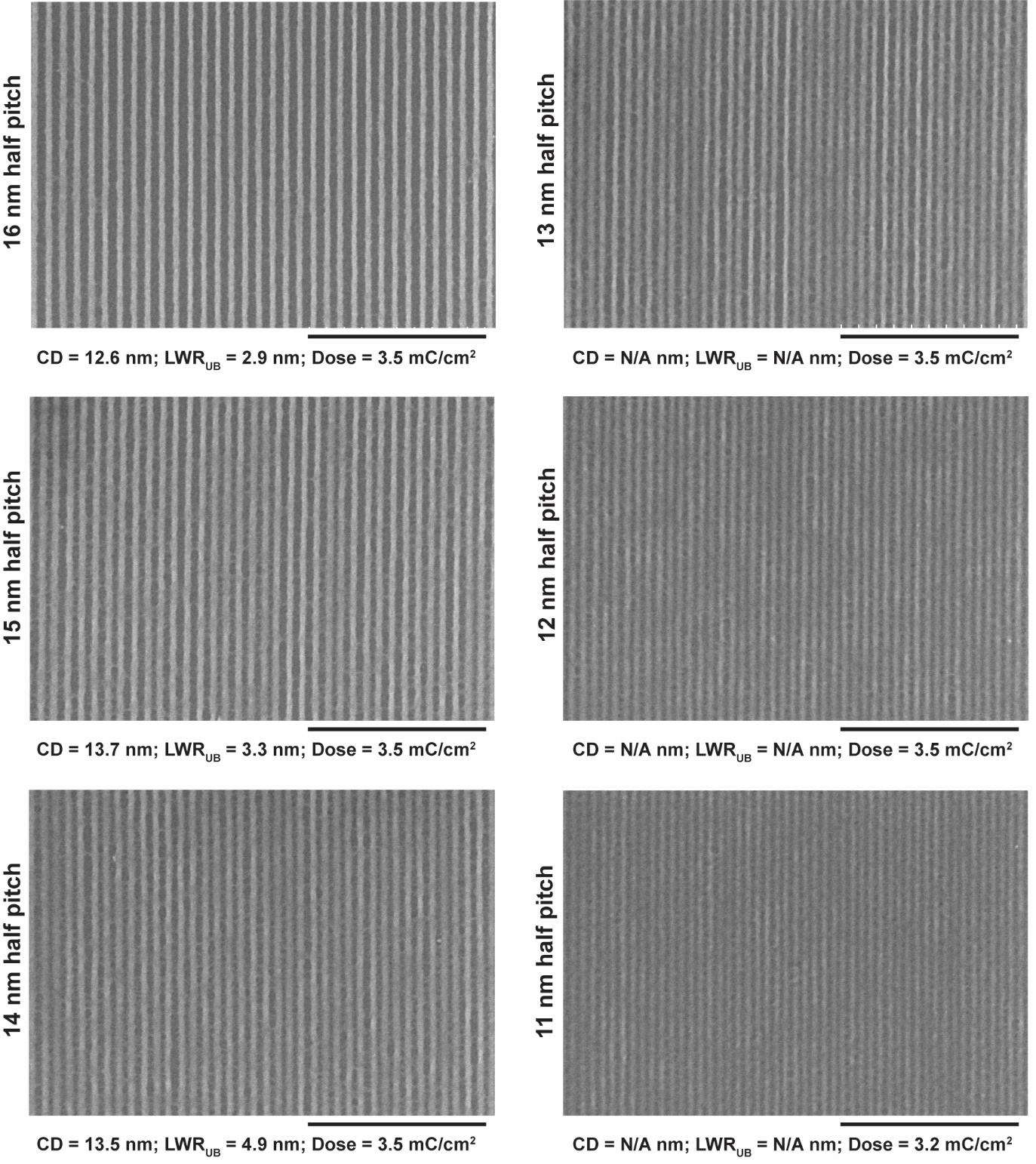


**Figure S17**: SEM images show the development of ZnMIP_2_ resist after EBL using PGMEA developer. Even though the required dose of exposure is low, sample developed using PGMEA starts to show the appearance of scum even at 16 nm half pitch. Unsurprisingly, the LWR_UB_ gets progressively worse.

**13. Simulation of the Point Spread Function (PSF) in ZnMIP_2_ Resist**

The PSF for ZnMIP_2_ resist was simulated by Monte-Carlo method using the TRACER software (GenISys GmbH). The film stack used for the simulation consisted of 30 nm ZnMIP_2_ resist on 500 µm Si substrate. The stoichiometry for ZnMIP_2_ resist was defined as H_12_C_8_N_2_O_6_Zn_1_. The incident beam energy of 100 keV and 92 eV was used for generating EBL and EUV-like PSF simulations, respectively. All simulations were performed over 1 million electrons.

At first, the 100 keV PSF was simulated for different resist densities (ρ) and as shown in Figure S18(a) only marginal change in the short-range regime (2-10 nm) was observed, However, when the density was fixed at 2 gm/cm^3^, and PSF was generated for different beam diameter Figure S18(b), substantial short-range proximity effect was observed. When a similar simulation was carried out for 92 eV incident electrons (EUV-like), an incremental increase in the short-range proximity effect can be seen with increasing beam diameter. However, as recent study suggests EUV generated low-energy electron spread between 1 and 2 nm,^13^ the effect on the patterned features is seemingly much smaller than that of 100 keV EBL.


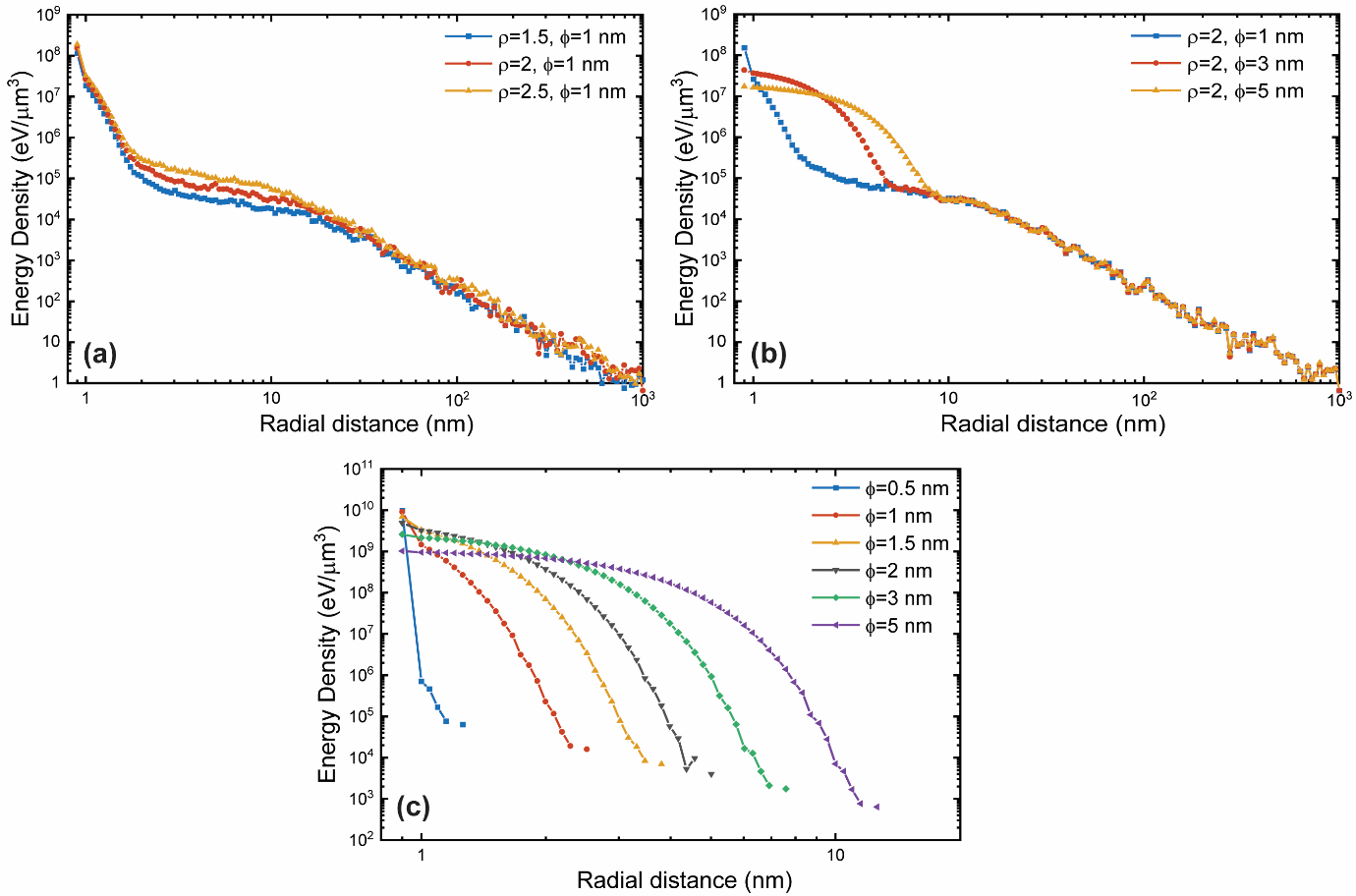


**Figure S18**: (a) PSF at 100 keV acceleration voltage for different resist densities (ρ), and (b) for different beam sizes (ϕ). (c) PSF for 92 eV incident electron energy (EUV-like) with different beam diameters.

**14. Simulation of Energy Deposited on The Resist During EBL**

Simulations were carried out to understand the energy deposited during EBL of 1:1 line/space features and single pass lines at different half pitches using 2D E-beam module of the BEAMER (GenISys GmbH) e-beam exposure simulation and pattern preparations software. Monte-Carlo simulated 100 keV PSF with 5 nm beam size and 2 gm/cm^3^ resist density [Figure S18(b)] was used for carrying out EBL simulations. The beam step size of 1 nm and simulation grid of 0.5 nm was used during all the simulations. After testing number of parameter combinations, 1 nm resist blur, development threshold of 0.5, relative energy and dose factor of 1.25 showed good agreement with experimental results of 1:1 line/space EBL patterns obtained using EGME developer (showed in Figure 4). Therefore, this condition was fixed for carrying out simulations on various 1:1 line/space and single-pass lines at different half pitches. The variation in dose factor and development threshold can be equivalently correlated to carrying out patterning dose test for with the use of developers with different contrast – lower the developer contrast, smaller the threshold for relative energy in the simulations.


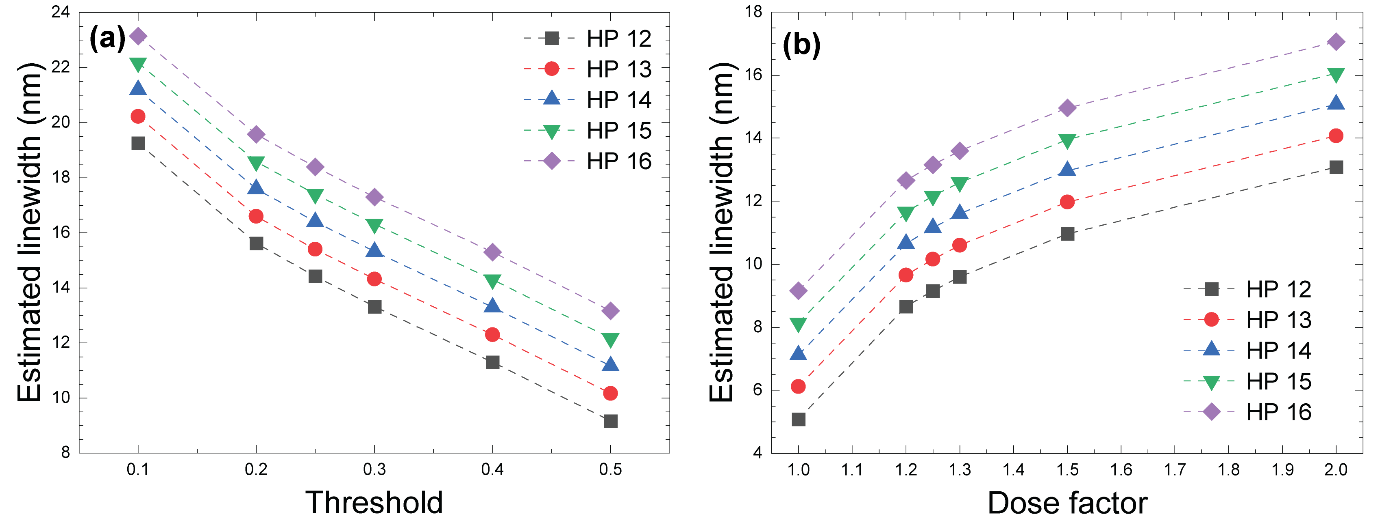


**Figure S19**: Estimated linewidths from e-beam simulations on ZnMIP_2_ resist with (a) different development thresholds at a dose factor of 1.25, and (b) different exposure dose factors at a development threshold of 0.5.


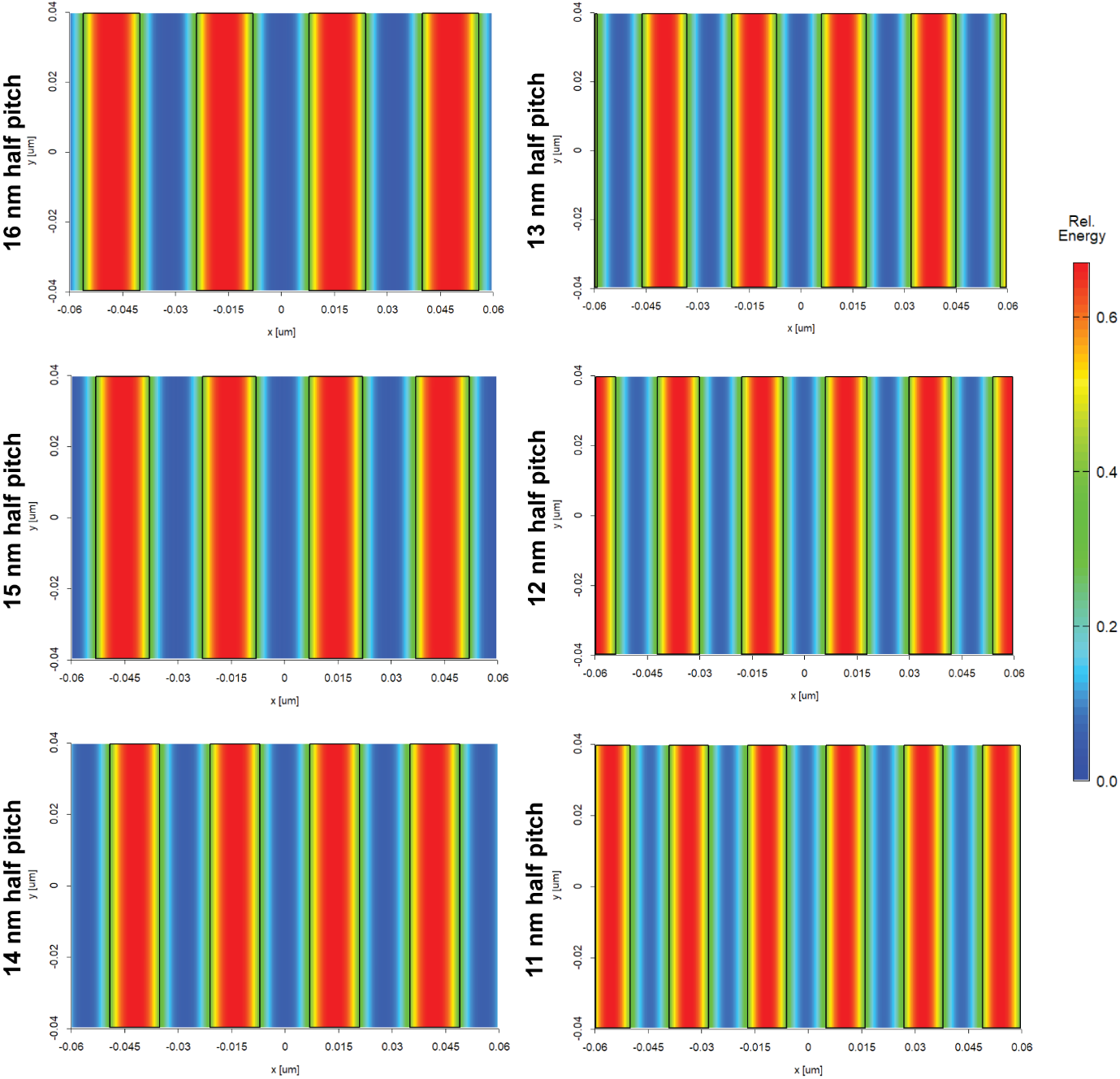


**Figure S20**: 2D e-beam simulation results showing energy deposited by 100 keV incident beam in different 1:1 line/space features. The black coloured overlaying boundary depicts the region exposed by the e-beam.


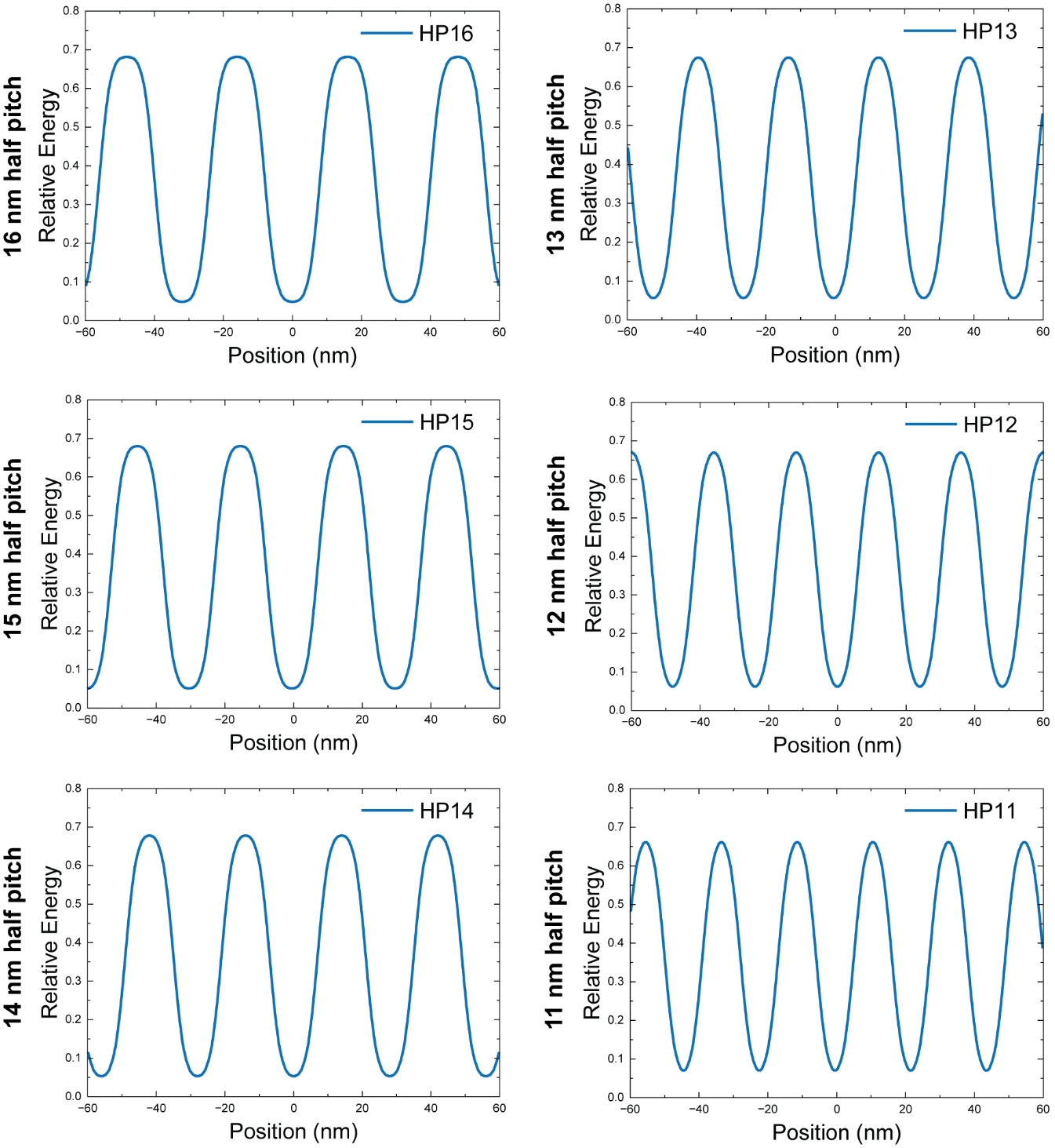


**Figure S21**: Cross-section profiles of 2D electron beam simulation results (shown in Figure S20) on 1:1 line/space features.


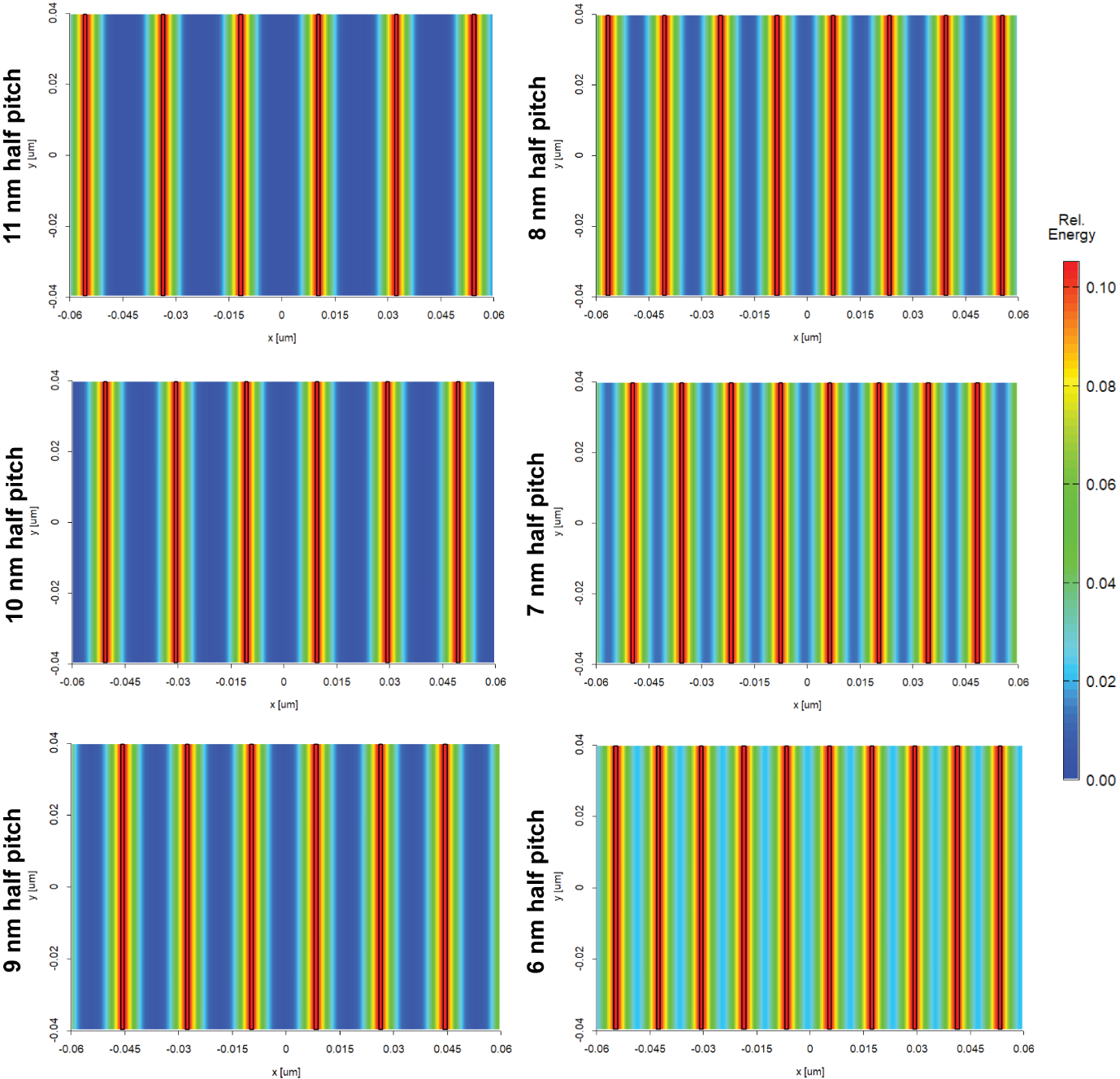


**Figure S22**: 2D electron beam simulation results showing energy deposited by 100 kV incident beam when single-pass lines are exposed at different half-pitches. The black coloured overlaying boundary depicts the region exposed by the electron beam.


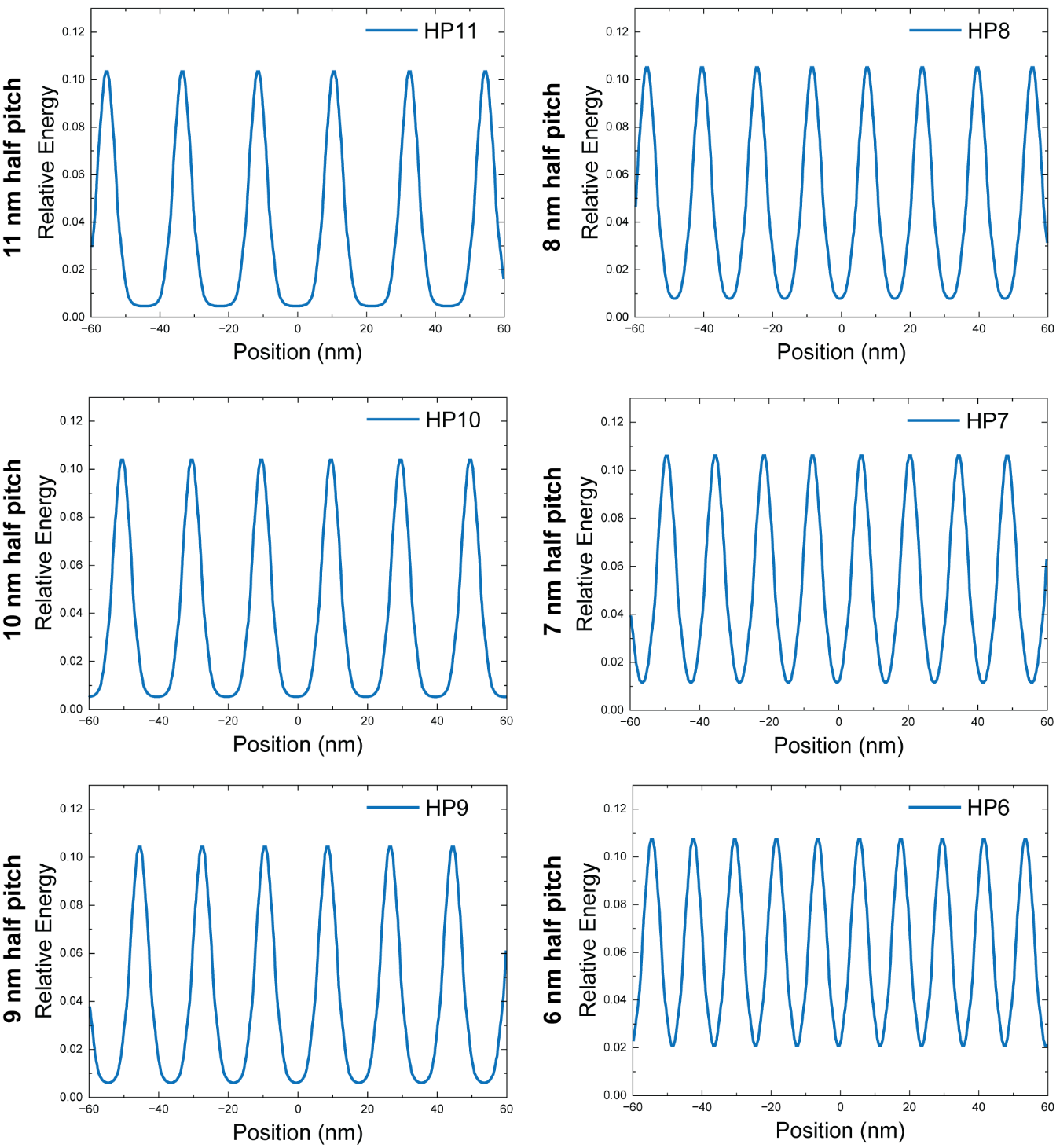


**Figure S23**: Cross-section profiles of 2D electron beam simulation results (shown in Figure S22) of single-pass lines patterned at different half-pitches.

**15.** **Measurement of Height of Lines and Effect of Increasing Electron Dose**


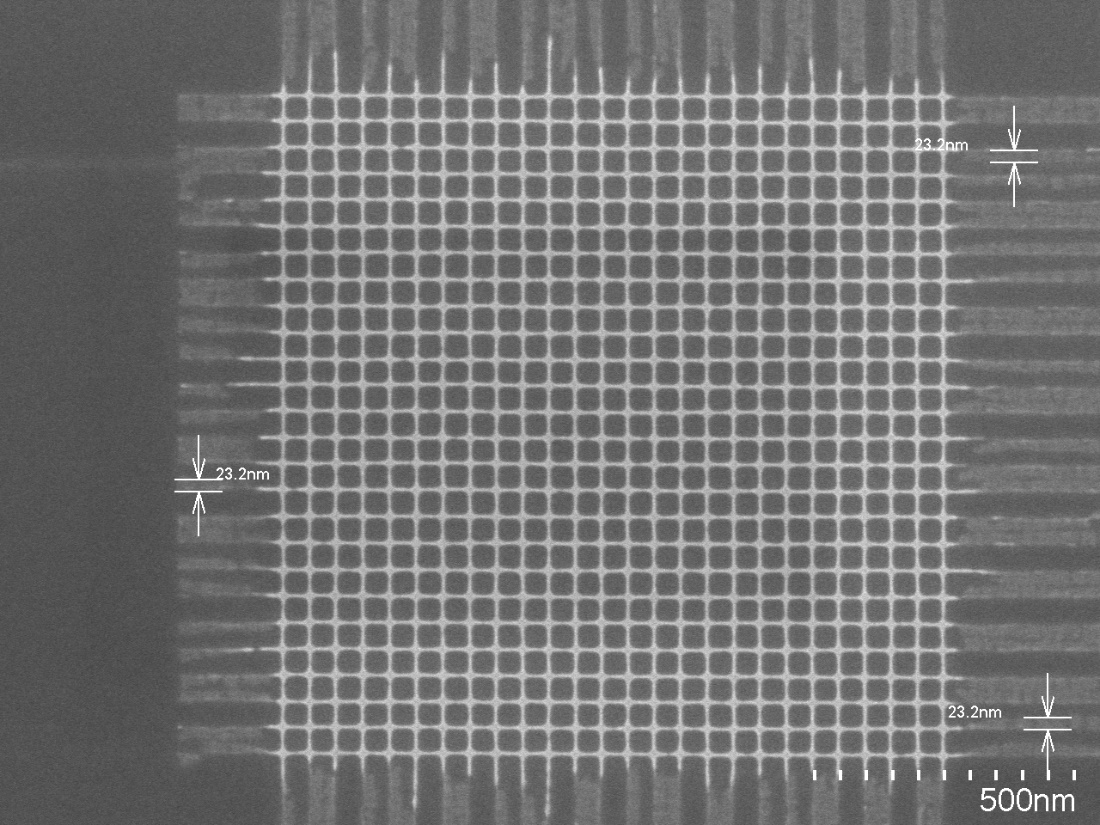


**Figure S24**: Height of the collapsed line measured using SEM. Patterning dose was 51.7 mC/cm^2^.


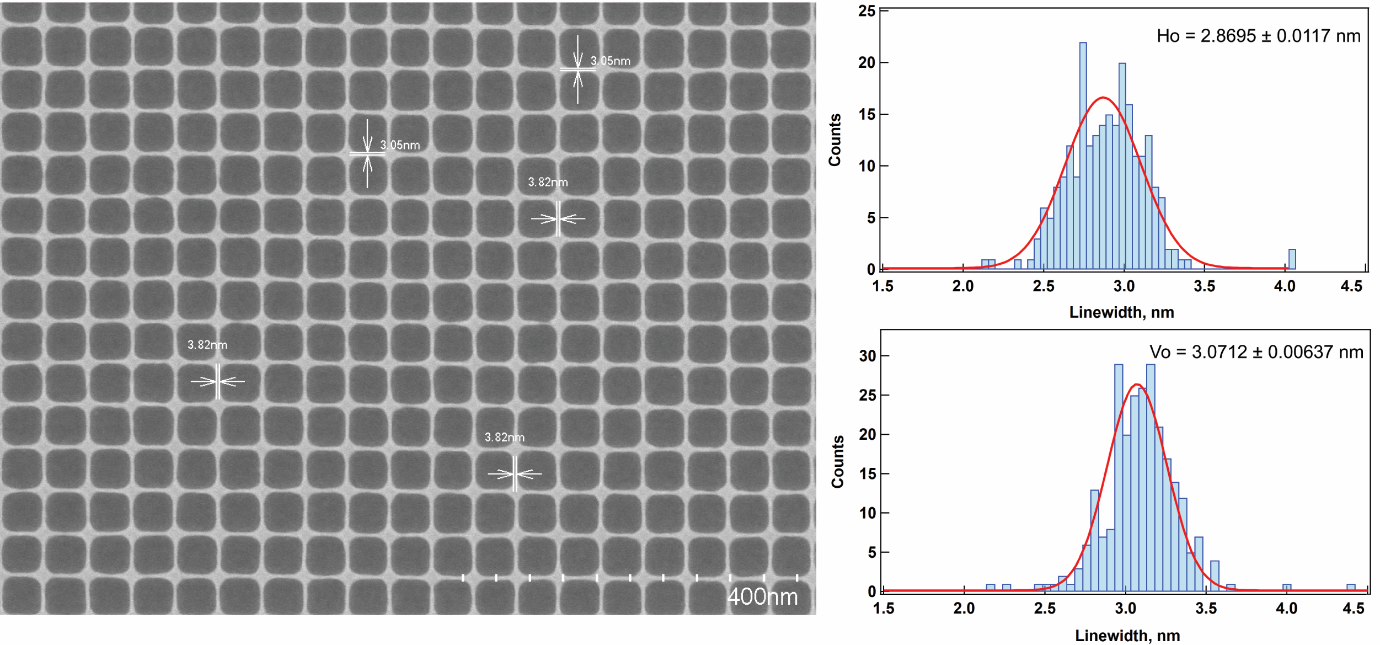


**Figure S25**: Electron beam lithography of single pass lines written as crossbar structure at an electron dose of 51.7 mC/cm^2^. Their corresponding line-width analysis is shown on the right-hand side. H_o_ and V_o_ in (a) corresponds to average line-widths of horizonal and vertical lines, respectively.

**16. Simulated Spot Size of Electron Beam in Vistec EBPG 5000Plus EBL Machine**

Figure S27 shows the relationship between beam current and beam diameter of Vistec EBPG 5000Plus electron beam writer. For our fine patterning studies, we used 300 mm aperture and 1 nA beam current. The theoretical spot size comes to ~5 nm.


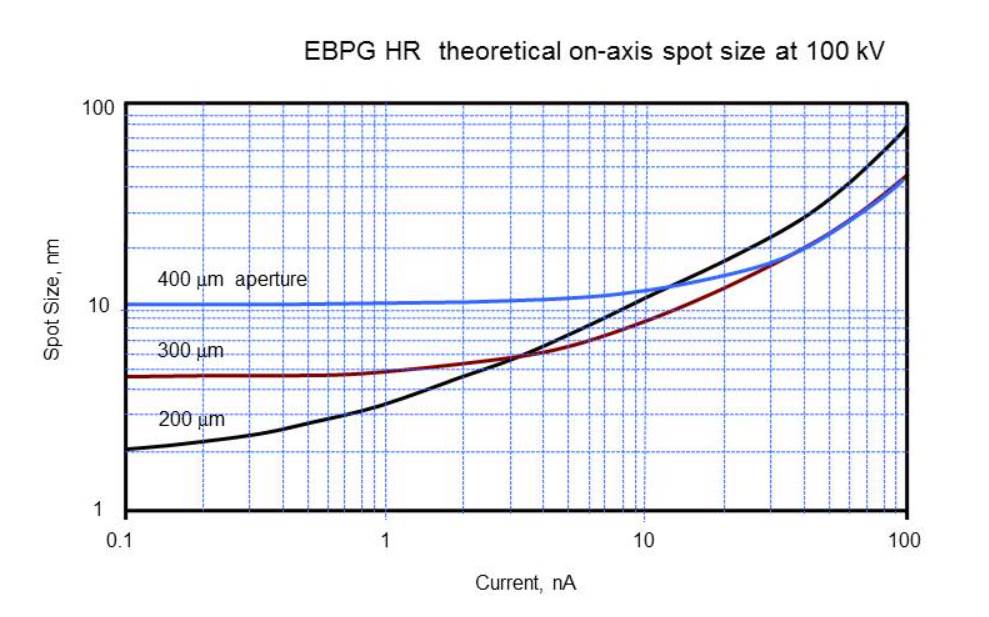


**Figure S26**: Relationship between beam current and theoretical beam diameter of Vistec EBPG 5000Plus electron beam writer operating at 100 kV.

**17. Designed Width Versus Patterned Width**

One of the interesting characteristics of metal oximate resists is that the designed dimensions and the ones observed in the patterns are off by ~30-40 nm depending upon the size of pattern to be written. When the percentage reduction in designed width is plotted against the designed width, an exponential relationship is seen (Figure S28). This suggests that at smaller designed widths, the patterned width could be close to 50% of the designed width. The reason for its strange behaviour is not understood well at the moment.


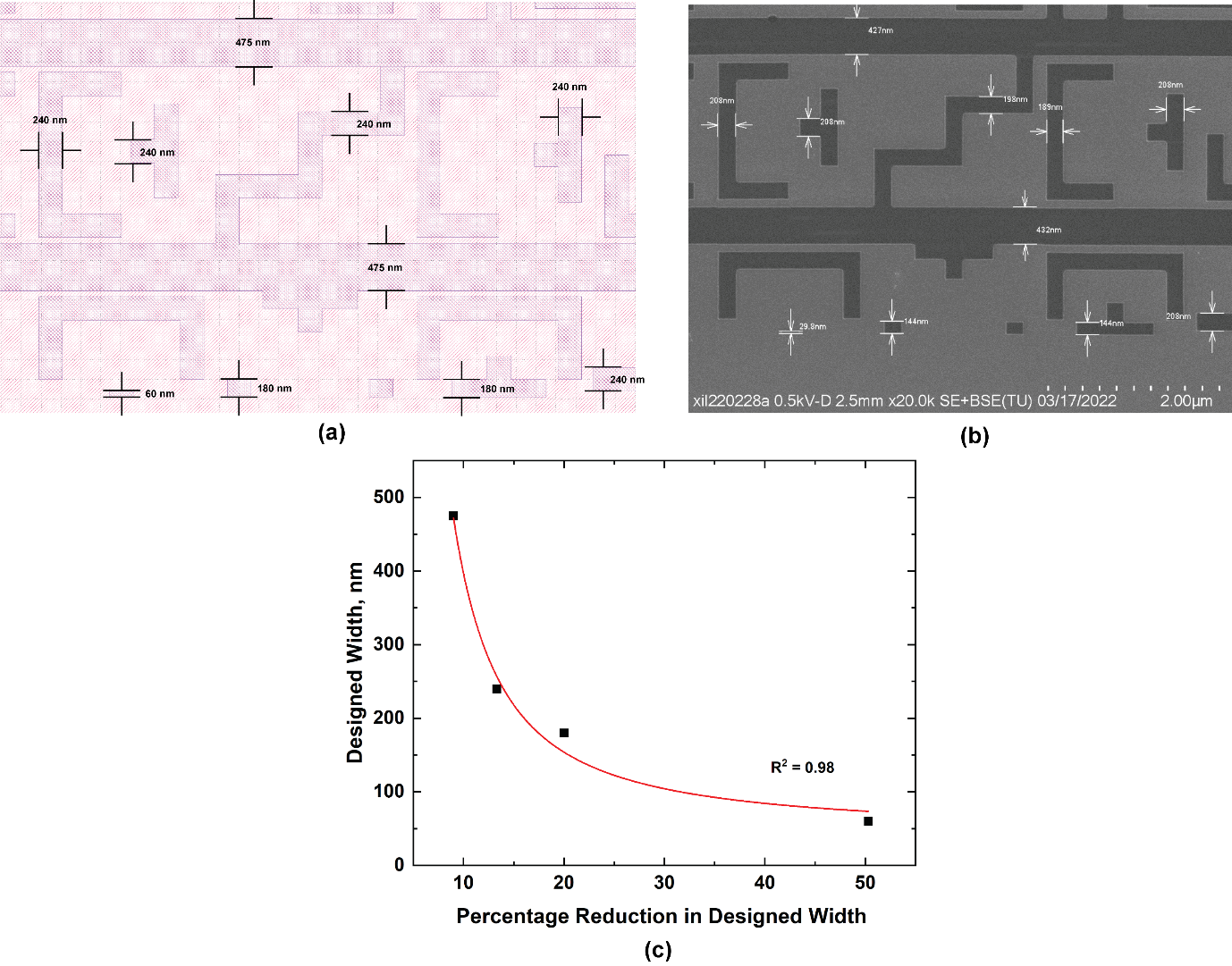


**Figure S27**: (a, b) Variation in designed width *versus* the patterned width in NiMIP_2_ resist. PGME was the developer. 10 sec development and 5 sec rinse in the same solvent. (c) The relationship between designed width and the percentage reduction in designed width after patterning.

**18. Z**–**Factor Calculations**

| **Half Pitch** | **DtS (mJ/cm^2^)** | **LWR (nm)** | **Z–factor (**$\times$**10^-8^) (mJ·nm^3^)** |
| --- | --- | --- | --- |
| 16 nm | 231 | 1.69 | 2.7 |
| 15 nm | 217 | 1.8 | 2.4 |
| 14 nm | 225 | 1.66 | 1.49 |
| 13 nm | 171.4 | 2.32 | 1.04 |

(a)

| **Half Pitch** | **DtS (mJ/cm^2^)** | **LWR (nm)** | **Z–factor (**$\times$**10^-8^) (mJ·nm^3^)** |
| --- | --- | --- | --- |
| 16 nm | 122 | 1.6 | 1.28 |
| 15 nm | 103.5 | 1.7 | 1.01 |
| 14 nm | – | – | – |
| 13 nm | 95 | 1.7 | 0.6 |

(b)

**Table S13:** Performance summary of EUVL patterns developed with (a) PGMEA and (b) anisole and their corresponding calculated Z–factors.

**19. Benchmarking of Resist Performance**

We have benchmarked our resist performance against the state-of-the-art reports from both academic and industry reports from past year and they are summarized in the following table.

| Resist Category | Half Pitch (nm) | LWR (nm) | Dose (mJ/cm^2^) | Z-factor $\boldsymbol{\times}$ 10^-8^ (mJ.nm^3^) | Tool | Ref |
| --- | --- | --- | --- | --- | --- | --- |
| CAR A | 14 | 2.64 | 38.1 | 0.73 | PSI | 14 |
| CAR A | 13 | 2.64 | 38.4 | 0.59 | PSI | 14 |
| CAR A | 12 | 2.81 | 42.2 | 0.57 | PSI | 14 |
| CAR B | 14 | 2.12 | 65.34 | 0.8 | PSI | 14 |
| CAR B | 13 | 1.90 | 52.8 | 0.42 | PSI | 14 |
| CAR B | 12 | 2.95 | 55.8 | 0.84 | PSI | 14 |
| CAR C | 14 | 1.50 | 36.7 | 0.22 | PSI | 14 |
| CAR C | 13 | 2.88 | 26 | 0.47 | PSI | 14 |
| MTR | 14 | 2.53 | 21.9 | 0.38 | PSI | 14 |
| MTR | 13 | 2.73 | 13.6 | 0.22 | PSI | 14 |
| MOR | 14 | 2.42 | 57.5 | 0.92 | PSI | 14 |
| MOR | 13 | 2.52 | 65.1 | 0.78 | PSI | 14 |
| MOR | 12 | 3.12 | 56.6 | 0.95 | PSI | 14 |
| MOR | 11 | 3.08 | 52.2 | 0.66 | PSI | 14 |
| MOR/ESPERT | 8 | 3.02 | 72 | 0.34 | MET5 | 15 |
| Molecular Glass | 18 | 2.70 | 191 | 8.12 | PSI | 16 |
| Molecular Glass | 16 | 3.11 | 212 | 8.40 | PSI | 16 |
| Molecular Glass | 15 | 3.54 | 185 | 7.80 | PSI | 16 |
| Molecular Glass | 14 | 3.11 | 205 | 5.44 | PSI | 16 |
| Molecular Glass | 13 | 3.54 | 167 | 4.58 | PSI | 16 |
| Molecular Glass | 12 | 6.22 | 172 | 11.50 | PSI | 16 |
| MO Complex | 20 | 1.50 | 132.3 | 1.28 | MET5 | 17 |
| MOR/ESPERT | 10 | 2.85 | 62 | 0.50 | MET5 | 18 |
| MOR/ESPERT | 9 | 2.63 | 67 | 0.34 | MET5 | 18 |
| MOR/ESPERT | 8 | 3.02 | 72 | 0.34 | MET5 | 18 |
| MOR | 14 | 3.02 | 45 | 1.13 | NXE | 19 |
| MOR | 12 | 2.68 | 39.5 | 0.49 | NXE | 19 |
| HSQ-Polymer | 14 | 3.20 | 120 | 3.37 | PSI | 20 |
| MTR | 14 | 3.80 | 64 | 2.54 | NXE 3400B | 21 |
| MTR | 14 | 3.57 | 69 | 2.41 | NXE 3400B | 21 |
| MOR/O-DDR | 14 | 2.25 | 47 | 0.65 | ? | 22 |
| MOR | 14 | 2.21 | 56 | 0.75 | NXE 3400B | 23 |
| MOR/O-DDR | 14 | 2.39 | 34 | 0.53 | NXE 3400B | 23 |
| CAR/UL | 14 | 1.7 | 58.2 | 0.46 | ? | 24 |
| CAR/MOR-UL | 14 | 4.04 | 65 | 2.91 | NXE 3400 | 25 |
| CAR/MOR-UL | 12 | 4.81 | 46.9 | 1.88 | NXE 3400 | 25 |
| MOR/UL | 14 | 3.30 | 68.9 | 2.06 | NXE 3400B | 26 |
| MOR/UL | 14 | 3.01 | 70.2 | 1.75 | NXE 3400B | 26 |
| MOR/UL | 14 | 3.11 | 68.9 | 1.83 | NXE 3400B | 26 |
| MOR/UL | 14 | 3.07 | 67.6 | 1.75 | NXE 3400B | 26 |

**Table S14:** Benchmarking of various categories of resists. CAR–Chemically Amplified Resist, MTR–Multi Trigger Resist, MOR–Metal Oxide Resist, UL–underlayer. In case the cited reference only reported LER values, we have converted them to LWR values using LWR = √2$\boldsymbol{\times}$LER.^27^

**References**

(1) Ahmad, A.; Spenser, I. D. The Conversion of α–Keto Acids and of α–Keto Acid Oximes to Nitriles in Aqueous Solution. *Can J. Chem.* **1961**, *39*, 1340–1359.

(2) Apblett, A. W.; Georgieva, G. D.; Mague, J. T. Synthesis and Spectroscopic and Thermal Decomposition Studies of Alkali Metal Salts of 2-Oximidopropionate. *Inorg. Chem.* **1997**, *36*, 2656–2661

(3) Hill, M. R.; Jones, A. W.; Russell, J. J.; Roberts, N. K.; Lamb, R. N. Towards New Precursors for ZnO Thin Films by Single Source CVD: The X-ray Structures and Precursor Properties of Zinc Ketoacidoximates, *Inorg. Chim. Acta* **2005**, *358*, 201–206.

(4) Schneider, J. J.; Hoffmann, R. C.; Engstler, J.; Dilfer, S.; Klyszcz, A.; Erdem, E.; Jakes, P.; Eichel, R. A. Zinc Oxide Derived from Single Source Precursor Chemistry Under *Chimie Douce* Conditions: Formation Pathway, Defect Chemistry and Possible Applications in Thin Film Printing. *J. Mater. Chem.* **2009**, *19*, 1449–1457.

(5) Georgieva, I.; Trendafilova, N.; Bauer, G. Spectroscopic and Theoretical Study of Cu(II), Zn(II), Ni(II), Co(II) and Cd(II) Complexes of Glyoxilic Acid Oxime. *Spectrochim. Acta A Mol. Biomol. Spectrosc.* **2006**, *63*, 403–415.

(6) Pashchanka, M.; Hoffmann, R. C.; Schneider, J. J. Controlled Synthesis and Characterisation of MgO Nanoparticles, Thin Films and Polycrystalline Nanorods Derived from a Mg(II) Single Source Precursor. *J. Mater. Chem.* **2010**, *20*, 957–963.

(7) Pashchanka, M.; Hoffmann, R. C.; Gurlo, A.; Schneider, J. J. Molecular Based, Chimie Douce Approach to 0D and 1D Indium Oxide Nanostructures. Evaluation of Their Sensing Properties Towards CO and H_2_. *J. Mater. Chem.* **2010**, *20*, 8311–8319.

(8) Hoffmann, R. C.; Dilfer, S.; Schneider, J. J. Transparent Indium Tin Oxide as Inkjet-Printed Thin Film Electrodes for Organic Field-Effect Transistors. *Phys. Status Solidi A* **2011**, *208*, 2920–2925.

(9) Khanderi, J.; Davaasuren, B.; Alshankiti, B. A.; Rothenberger, A. Tin(II) Ketoacidoximates: Synthesis, X-ray Structures and Processing to Tin(II) Oxide. *Dalton Trans.* **2015**, *44*, 19820–19828.

(10) Sanctis, S.; Koslowski, N.; Hoffmann, R.; Guhl, C.; Erdem, E.; Weber, S.; Schneider, J. J. Toward an Understanding of Thin-Film Transistor Performance in Solution-Processed Amorphous Zinc Tin Oxide (ZTO) Thin Films. *ACS Appl. Mater. Interfaces* **2017**, *9*, 21328−21337.

(11) Hansen, C. M.; Chapter 38: Solubility Parameters. In *Paint and Coating Testing Manual: 15th Edition of the Gardner-Sward Handbook*; Koleske, J. V., Ed.; ASTM International: West Conshohocken, PA; 2012, pp. 470−494.

(12) *Hansen Solubility Parameters: A User’s Handbook (2^nd^ Edition)*; Hansen, C. M., Ed.; CRC Press: Boca Raton, FL; 2007, Appendix A.

(13) Fallica, R.; Mahne, N.; Conard, T.; Vanleenhove, A.; de Simone, D.; Nannarone, S. Mean Free Path of Electrons in Organic Photoresists for Extreme Ultraviolet Lithography in the Kinetic Energy Range 20−450 eV. *ACS Appl. Mater. Interfaces* **2023**, *15*, 35483−35494.

(14) Develioglu, A.; Vockenhuber, M.; van Lent-Protasova, L.; Mochi, I.; Ekinci, Y.; Kazazis, D. Advancements in EUV Photoresists for High-NA Lithography. *Proc. SPIE* **2023**, *12750*, 1275008.

(15) Dinh, C. Q.; Nagahara, S.; Kuwahara, Y.; Dauendorffer, A.; Okada, S.; Fujimoto, S.; Kawakami, S.; Shimura, S.; Muramatsu, M.; Cho, K.; Liu, X.; Nafus, K.; Carcasi, M. A.; Agarwal, A.; Somervell, M. H.; Huli, L.; Kato, K.; Kocsis, M.; De Schepper, P.; Meyers, S. T.; McQuade, L.; Kasahara, K.; Garcia Santaclara, J.; Hoefnagels, R.; Anderson, C.; Naulleau, P. Advanced Development Methods for High-NA EUV Lithography. *Proc. SPIE* **2023**, *12498*, 1249806.

(16) Wang, Y.; Chen, J.; Zeng, Y.; Yu, T.; Wang, S.; Guo, X.; Hu, R.; Tian, P.; Vockenhuber, M.; Kazazis, D.; Ekinci, Y.; Wu, Y.; Yang, S.; Zhao, J.; Yang, G.; Li, Y. Nonchemically Amplified Molecular Resists Based on Sulfonium-Functionalized Sulfone Derivatives for Sub-13 Nm Nanolithography. *ACS Appl. Nano Mater.* **2023**, *6*, 18480–18490.

(17) Lim, G.; Lee, K.; Koh, C.; Nishi, T.; Yoon, H. J. Multinuclear Tin-Based Macrocyclic Organometallic Resist for EUV Photolithography. *ACS Mater. Au* **2024**.

(18) Dinh, C. Q.; Nagahara, S.; Cho, K.; Tomori, H.; Kuwahara, Y.; Onitsuka, T.; Okada, S.; Kawakami, S.; Hara, A.; Fujimoto, S.; Muramatsu, M.; Tsuzuki, R.; Liu, X.; Thiam, A.; Feurprier, Y.; Nafus, K.; Carcasi, M. A.; Huli, L.; Kato, K.; Krawicz, A.; Kocsis, M.; De Schepper, P.; McQuade, L.; Kasahara, K.; Garcia Santaclara, J. G.; Hoefnagels, R.; La Fontaine, B.; Miyakawa, R. H.; Anderson, C. N.; Naulleau, P. P. Advanced Processes in Metal-Oxide Resists for High-NA EUV Lithography. *Proc. SPIE* **2024**, *12957*, 1295705.

(19) Castellanos, S.; De Schepper, P.; Bhattacharya, M.; Doise, J.; Wouters, J.; Narasimhan, A. K.; Cardineau, B.; McQuade, L.; Needham, C. D.; Kocsis, M.; Kasahara, K.; Meyers, S. T. EUV Metal Oxide Resists: Impact of the Environment Composition on CD during Post-Exposure Delay. *Proc. SPIE* **2024**, *12957*, 1295707.

(20) Dang, L. N.; Tseng, L.-T.; Rajak, A.; Gädda, T.; Laukkanen, M.; Salunke, J.; Moosakhani, S.; Paulasaari, J.; Rantala, J.; Vockenhuber, M.; Kazazis, D.; Ekinci, Y. Designing EUV Negative Tone Resist and Underlayer Approaches Exhibiting 14nm Half-Pitch Resolution. *Proc. SPIE* **2024**, *12957*, 129570I.

(21) Popescu, C.; O’Callaghan, G.; McClelland, A.; Storey, C.; Roth, J.; Jackson, E. A.; Robinson, A. P. G. EUV Lithography Patterning Using Multi-Trigger Resist. *Proc. SPIE* **2024**, *12957*, 129570R.

(22) Heo, S.; Baek, S.; Gupta, M.; Suh, H. S.; Kato, K.; Takeda, S.; Shibayama, W.; Sakamoto, R. Organic Dry Development Rinse (O-DDR) Process for Spin-on MOR to Prevent Pattern Collapse. *Proc. SPIE* **2024**, *12957*, 1295710.

(23) Takeda, S.; Shibayama, W.; Sakamoto, R.; Shigaki, S.; Furukawa, Y.; Saijo, T.; Kato, K.; Heo, S.; Suh, H. S. Organic Dry Development Rinse (O-DDR) Process for MOR Patterning toward High-NA EUV. *Proc. SPIE* **2024**, *12957*, 1295702.

(24) Fallica, R.; Verstraete, L.; Huang, W.; Gupta, M.; De Simone, D.; Li, S.; Sweat, D.; Guerrero, D. J.; Kato, K. Functional Underlayers, Surface Priming, and Multilayer Stacks to Improve Dose and Adhesion of EUV Photoresists. *Proc. SPIE* **2024**, *12957*, 1295712.

(25) Li, Z.; Kampitakis, V.; Her, Y.; Antonio, C.; Kudo, T.; Mullen, S.; Muthuswamy, E.; Polishchuk, O.; Ware, A.; Wolfer, E.; Yang, D.; Cho, J.-Y.; Hishida, A.; Sekito, T. Spin-on Metal-Oxide Underlayer for EUV Patterning. *Proc. SPIE* **2024**, *12957*, 1295713.

(26) Li, S.; Lu, X.; Grannemann, S.; Sweat, D.; Brakensiek, K. E.; Lu, P.; Lowes, J. A.; Krishnamurthy, V.; Van Driessche, V.; Guerrero, D. Lithography Performance Improvement of MOR by Underlayers. *Proc. SPIE* **2024**, *12957*, 1295715.

(27) Brunner, T. A.; Chen, X.; Gabor, A.; Higgins, C.; Sun, L.; Mack, C. A. Line-Edge Roughness Performance Targets for EUV Lithography. *Proc. SPIE* **2017**, *10143*, 101430E.

1. * Corresponding authors’ email addresses: M.S.M.S.: [msm.saifullah@psi.ch](mailto:msm.saifullah@psi.ch) ; Y.E.: [yasin.ekinci@psi.ch](mailto:yasin.ekinci@psi.ch) [↑](#footnote-ref-1)
